# Putting the Puzzle Together To Get the Whole Picture: Molecular Basis of the Affinity of Two Steroid Derivatives to Acetylcholinesterase

**DOI:** 10.1021/acsomega.3c03749

**Published:** 2023-07-06

**Authors:** Victoria Richmond, Bruno N. Falcone, Marta S. Maier, Pau Arroyo Máñez

**Affiliations:** † Facultad de Ciencias Exactas y Naturales, Departamento de Química Orgánica, Universidad de Buenos Aires, Buenos Aires C1428EGA, Argentina; ‡ Unidad de Microanálisis y Métodos Físicos aplicados a la Química Orgánica (UMYMFOR), CONICET-Universidad de Buenos Aires, Pabellón 2 de Ciudad Universitaria, Buenos Aires C1428EGA, Argentina; § Instituto Interuniversitario de Investigación de Reconocimiento Molecular y Desarrollo Tecnológico (IDM), Universitat Politècnica de València, Universitat de València, Doctor Moliner 50, Burjassot, Valencia 46100, Spain; ∥ Departamento de Química Orgánica, Universitat de València, Doctor Moliner 50, Burjassot, Valencia 46100, Spain

## Abstract

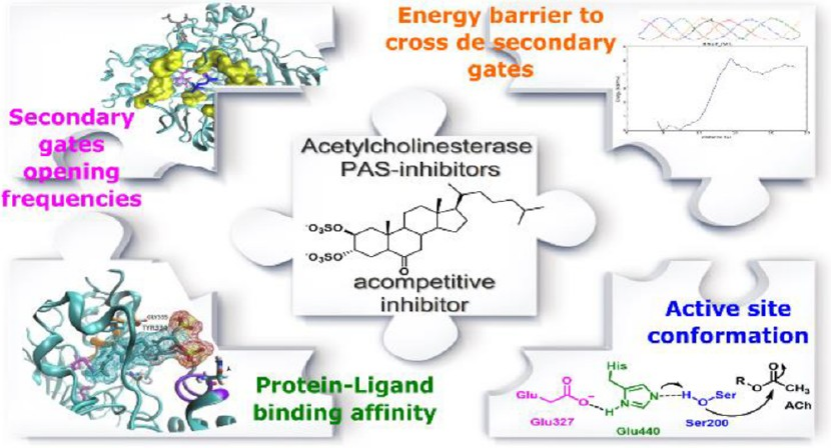

Alzheimer’s disease (AD) is a progressive neurodegenerative disorder that has no cure because its etiology is still unknown, and its main treatment is the administration of acetylcholinesterase (AChE) inhibitors. The study of the mechanism of action of this family of compounds is critical for the design of new more potent and specific inhibitors. In this work, we study the molecular basis of an uncompetitive inhibitor (compound **1**, 2β, 3α-dihydroxy-5α-cholestan-6-one disulfate), which we have proved to be a peripheral anionic site (PAS)-binding AChE inhibitor. The pipeline designed in this work is key to the development of other PAS inhibitors that not only inhibit the esterase action of the enzyme but could also modulate the non-cholinergic functions of AChE linked to the process of amylogenesis. Our studies showed that **1** inhibits the enzyme not simply by blocking the main gate but by an allosteric mechanism. A detailed and careful analysis of the ligand binding position and the protein dynamics, particularly regarding their secondary gates and active site, was necessary to conclude this. The same analysis was executed with an inactive analogue (compound **2**, 2β, 3α-dihydroxy-5α-cholestan-6-one). Our first computational results showed no differences in affinity to AChE between both steroids, making further analysis necessary. This work highlights the variables to be considered and develops a refined methodology, for the successful design of new potent dual-action drugs for AD, particularly PAS inhibitors, an attractive strategy to combat AD.

## Introduction

Alzheimer’s disease (AD) is a neurodegenerative brain disorder characterized by a progressive memory loss, a decline in language skills, and other cognitive dysfunctions.^1^ According to the AD International, over 55 million people live with dementia, AD being the most common form. The World Health Organization has stated that about 139 million people would be affected with AD by the year 2050 due to the increase in life expectancy. This goes hand in hand with the increasing mortality and morbidity rate of AD.^2^

The etiology of this disorder remains unclear but there are different hypotheses regarding its causes such as the cholinergic hypothesis, amyloid hypothesis, tau propagation hypothesis, mitochondrial cascade hypothesis, among others. However, as the understanding of this disease improves, more potential targets are known for AD therapy—different receptors, proteins (APP, Aβ, tau), enzymes (lipoxygenase, acetylcholinesterase, etc.), oxidative imbalance, and RNA interference.^3^

Among all the physiopathologies in AD, lower levels of the neurotransmitter acetylcholine (ACh) in the brains of patients are observed. ACh regulates the memory and learning process and the cognitive performance.^4^ The selective and irreversible deficiency of cholinergic functions results in the memory impairment in AD, and this is what the cholinergic hypothesis proposes.^5^ Enhancement of ACh concentration in the brain, which compensates the cholinergic neurotransmitter deficit by inhibition of its hydrolytic enzymes (i.e., the cholinesterases), has provided the first generation of drugs for the treatment of AD. Nowadays, three of the four drugs administered for AD approved by the U.S. Food and Drug Administration are acetylcholinesterase inhibitors (AChEIs).^6^ Enhancing the cholinergic transmission produces modest but statistically significant improvements in the cognitive and global functions in mild to moderate AD.^7^

Moreover, another significant pathology observed during the disease is the abnormal plaque formation in the brain. These plaques are soluble β-amyloid oligomers or insoluble amyloid fibrils deposited in the area of brain parenchyma and cerebral blood vessel walls.^8^ It is reported that AD patients using cholinesterase inhibitors showed a significant decrease of plaque formation^9−12^ and as a consequence, AChEIs have shown more promising results in the treatment of AD than any other strategy explored.^13−16^

The three FDA-approved AChEI have problems and limitations, such as pharmacokinetic disadvantages and side effects due to the high doses needed to be administrated.^17^ Moreover, since the latest anti-AD drug approved in 2003, more than 100 anti-AD drug candidates have been rejected, many of them in advanced phases, due to efficacy or safety issues. This fact highlights that the AD drug development has one of the highest attrition rates in all therapeutic areas.^18,19^ For this reason, the discovery and development of new drugs to treat this disease are highly of the paramount picture.

There is a broad spectrum of AChEI obtained from natural products or by synthesis. Among the natural ones, these are mostly alkaloids isolated from plants, fungi, or marine organisms. Regarding the synthetic inhibitors, these are mainly synthetic derivatives or inspired by the FDA commercial drugs and tacrine, or strong hybrid inhibitors.^20^ Among steroidal AChEI, most are alkaloids. For example, five potent steroidal alkaloids were isolated from the bulbs of *Fritillaria walujewii* and kinetic studies revealed that these are mixed-type inhibitors.^21^ A research group obtained three new steroidal alkaloids from the bark of *Holarrhena pubescens* with strong AChE inhibiting activity and IC_50_ values ranging from 1.44 to 23.22 μM.^22^ Recently Liu and co-workers isolated a novel sterol with an unprecedented polycyclic ring system from the mushroom of *Tricholoma matsutake* with a moderate inhibitory activity (IC_50_ = 20.9 μM).^23^

**Figure 1:**
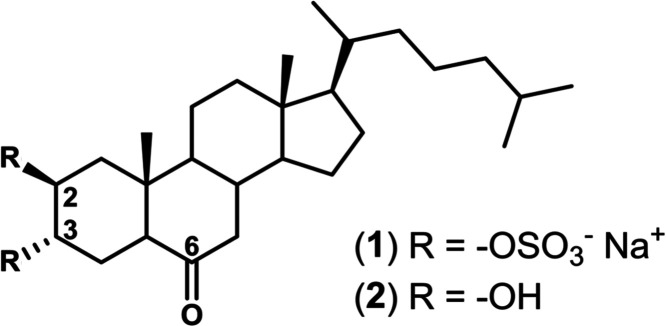
Chemical structure of compounds **1** and **2**.

In the search for AChE inhibitors and following this line of work, different steroids with sulfate groups at the C-2 and C-3 positions were synthesized,^24,25^ including the compound 2β, 3α-dihydroxy-5α-cholestan-6-one disulfate (**1**, Figure 1), the most active of the set (IC_50_ = 14.59 ± 0.88 μM). On the other hand, the desulfated analogue, **2** (2β,3α-dihydroxy-5α-cholestan-6-one, Figure 1) did not show inhibitory activity (IC_50_ > 200 μM). To gain insight into the mechanism of action of **1**, the active steroid **1** and inactive analogue **2** were studied by computational methods. Molecular docking studies were performed with the AChE crystal structure, complexed with ACh based on previous kinetic evidence,^24^ which revealed that **1** is an uncompetitive inhibitor of the enzyme, i.e., the compound binds reversibly to the enzyme–substrate complex (AChE–ACh), resulting in a loss of activity. The docking results indicate that both steroids bind to the enzyme into the peripheral anionic site (PAS, a region rich in aromatic amino acids located at the entrance of the enzyme pocket), penetrating the gorge with the aliphatic side chain. Therefore, the question arises as to why **1** is active while **2** is not. In this work, we present computer simulations that allow us to understand the molecular mechanism of AChE inhibition by these compounds. These simulations not only help to rationalize the mechanism of inhibition of these compounds, but they could be applied to other uncompetitive inhibitors that bind to the PAS or other allosteric inhibitors.

## Results and Discussions

Prior to molecular dynamics (MD) simulations of the acetylcholinesterase-steroid complexes (AChE-**1** and AChE-**2** complexes), a first simulation was achieved with the AChE–ACh complex to verify protein stability and to determine if the enzyme undergoes structural rearrangements. Protein backbone root-mean-square deviation (RMSD) from a 500 ns MD simulation revealed AChE stability from 15 ns onward, with an average RMSD value of around 2.0 Å with respect to the X-ray structure (Figure S1, Supplementary Material). The alternating PAS closing and opening processes were observed, according to the “breathing motion” described for the protein, which is necessary for a fast movement in/out of the active site by substrates and products.^26^ The distance between carbon CZ of Phe330 and the phenol oxygen of Tyr120, both residues in the bottleneck of the enzyme, evidenced this movement, and it is represented in Figure S2 (Supplementary Materials). The closed form is the favored conformation.^27,28^

### Binding Mode and Stability of the AChE-1 Complex

The docking studies allowed us to establish the binding mode of the uncompetitive inhibitor **1** to the AChE–ACh complex.^24^ The conformation of the most populated docking cluster was taken as the starting point to perform two 500 ns MD simulations, to check the enzyme–inhibitor complex stability. In this conformation, the steroid is located at the PAS, leaving steroidal ring A with one sulfate moiety exposed to the solvent and the other sulfate moiety interacting with the protein.

**Figure 2:**
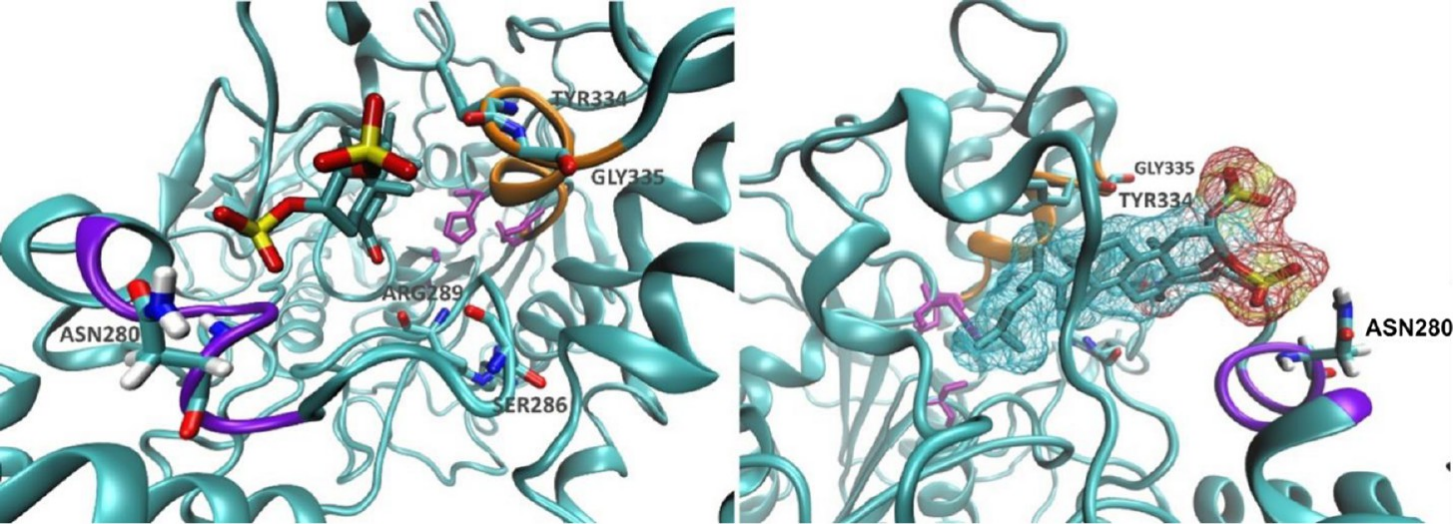
Two different perspectives of **1** inside AChE during the simulation (MD1). Helix 13 is represented in violet; helix 15, in orange; and the catalytic triad residues are in purple licorice representation.

During this MD simulation (MD1), the steroid fitted into the gorge, within the enzyme PAS, and the anionic sulfated substituent located on C-3 interacts with the backbone amide groups of helix 13 (Val277 to Leu282), which causes the steroid to adopt its conformation within the binding site (Figure 2). The sulfated group on C-2 was exposed to the solvent most of the time but was near helix 15 (Gly328 to Ala336) and interacts with these residues water-mediated. Compound **1** exposed its β face to helix 15 and remained at the same conformation during the simulation, allowing the carbonyl group to interact, directly or water-mediated, with Phe284, Ser286, Phe288, or Arg289 amides (Figure S3, Supplementary Materials). The stability of the complex was monitored by RMSD of the protein backbone (1.6 ± 0.3 Å) and RMSD of heavy atoms in **1** (5.5 ± 0.9 Å from ∼44 ns onwards, Figure S4, Supplementary Materials).

**Figure 3:**
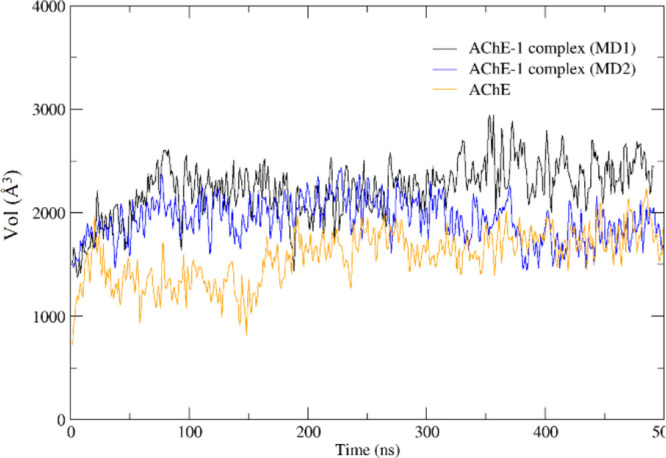
AChE pocket volume in the apoenzyme (orange), and in the AChE-1 complex during MD1 (black) and MD2 (blue).

Hydrophobic interactions are the main ones that anchor the steroid within the enzyme and are denoted by the lesser RMSD of carbon atoms of rings C and D and side chain (3.4 ± 0.6 Å from ∼44 ns onward) than the RMSD of ring A heavy atoms (including its substituents) (RMSD 7.5 ± 0.9 Å from ∼44 ns onwards, Figure S5, Supplementary Materials). This A-ring mobility is explained by the increased volume of the pocket due to the presence of the steroid (Figure 3); PAS flexibility^29^ allows ligands to expand it.^30^ This, along with the fact that the sulfate groups are located at the entrance of the pocket and exposed to the solvent, causes dynamic H-bonds for these groups. The steroid has enough space to present mobility, and H-bonds were able to break and re-form rapidly (in the picosecond to nanosecond timescale) with water molecules and protein residues.

**Figure 4:**
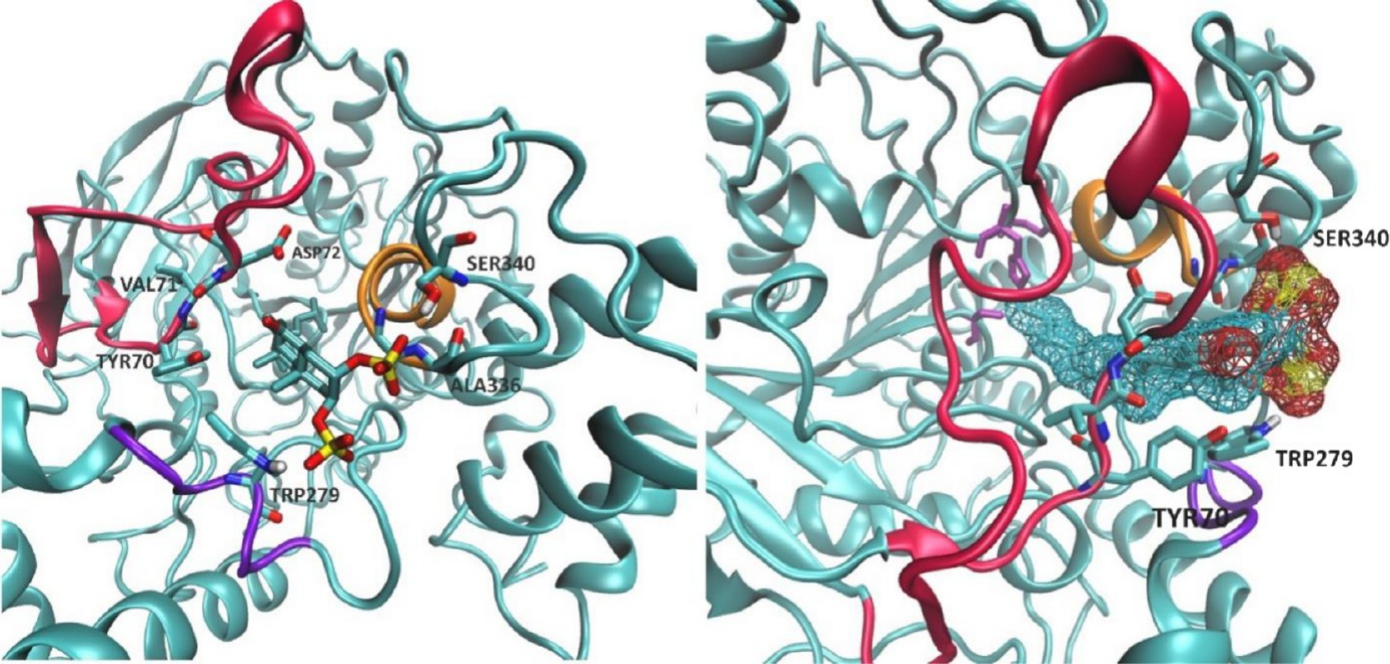
Conformation adopted by compound **1** inside AChE from 400 ns onwards, in MD2. The Ω-loop is represented in red; helix 13, in violet; and helix 15, in orange.

Another 500 ns MD simulation (MD2) was performed in order to increase sampling simulation time, starting from the docking conformation. MD2 revealed that at 400 ns, compound **1** rotated 180° around its longitudinal axis with respect to the conformation adopted in MD1 (Figure 4). MD2 also showed a PAS expansion (Figure 3) that may allow this steroid rotation, fitting the steroid better into the gorge and, then, reducing the cavity volume to values like those of the enzyme without a ligand. However, given the (2β,3α) configuration, the sulfated groups are also able to interact simultaneously with helixes 13 and 15. In this case, the sulfated group at C-3 is near helix 15, while the sulfated group at C-2 is near helix 13. This conformation allowed the carbonyl group (C-6) to interact with the hydroxyl group of Tyr70 or the NH_2_ of the amide group of Gln74, both residues belonging to the Ω-loop (Cys69 to Cys96), by water-mediated hydrogen bonding. This rotation raised the RMSD of the ligand up to 6.9 ± 0.4 Å at 400 ns and remained stable until the end of the simulation (Figure S6, Supplementary Materials). Protein backbone RMSD was stable along the simulation (1.8 ± 0.2 Å, Figure S6, Supplementary Materials). Once again, no particular H-bond between the sulfate groups and the enzyme was observed.

**Figure 5:**
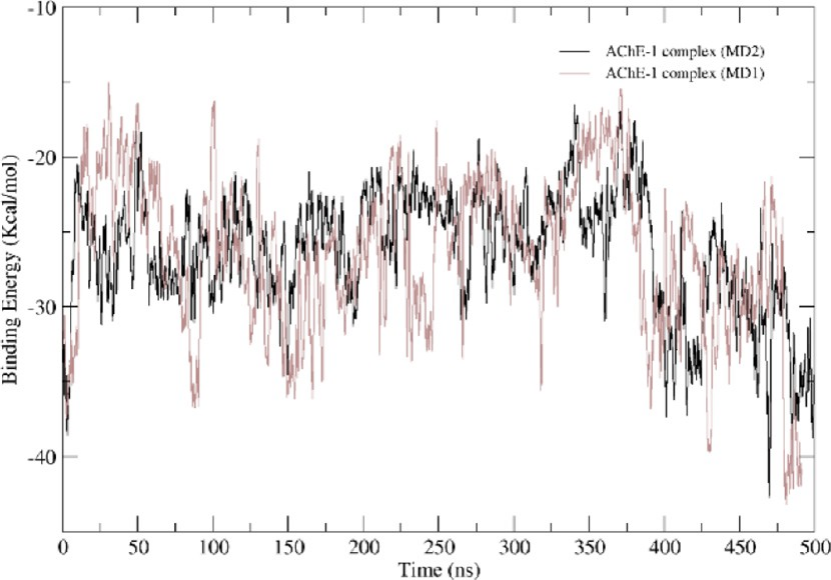
MM-GBSA free energy graph for the AChE-**1** complex. MD1 is represented in red and MD2, in black.

Although the same hydrogen bonds are not observed during MD1 and MD2, they are very similar as verified by the free binding energy of the ligands to the enzyme. The average values over the entire simulation were 26.4 ± 5.4 and 26.4 ± 6.4 kcal/mol, respectively, for MD1 and MD2 (Figure 5). During MD1, an extra stabilization of ∼5 kcal/mol is observed when the steroid penetrated deeper into the gorge and placed the carbonyl group near Phe284 (2.06 Å, 158°) at about 350 ns. Likewise, the complex AChE-**1** in MD2 also presents a conformation of 5 kcal/mol more stabilized when the steroid rotates 180° around its longitudinal axes and places the carbonyl group near the Ω-loop.

### Binding Mode and Stability of the AChE-2 Complex

Compound **2** was docked into AChE and a similar conformation to the one adopted by **1** was observed, even though **1** inhibits the protein but **2**, does not. Two 500 ns MD simulations of this complex were performed starting from the docking conformation to check the stability of the enzyme–inhibitor complex. Both simulations showed that the AChE-**2** complex was stable with a protein backbone RMSD of 2.4 ± 0.3 Å (MD3) and 2.1 ± 0.3 Å (MD4) and compound **2** RMSD of 4.5 ± 0.6 Å (MD3) and 2.8 ± 0.5 Å (MD4) (Figures S7 and S8, respectively). This result, at first, was not expected according to the experimental results.

**Figure 6:**
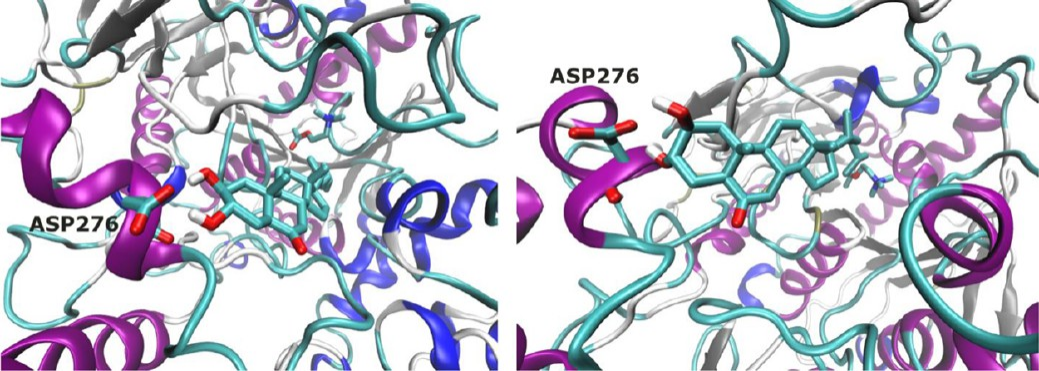
Conformation compound **2** adopted inside AChE in MD3. Twist conformation (left) and chair conformation (right). Cartoon backbone protein representation colored by the secondary structure.

Most of the time in MD3, the steroid exposed both hydroxyl groups to helix 13 but only the C-3 hydroxyl group and the carboxylate group of Asp276 were involved in a hydrogen bond (76% occupancy, 1.63 Å, 165.6°, Figure S9, Supplementary Materials), while the C-2 hydroxyl group was exposed to the solvent (Figure 6, right). Unlike compound **1**, compound **2** is not able to interact with both helixes 15 and 13 simultaneously due to the smaller size of the hydroxyl groups at ring A. However, during a brief time (from 475 ns onwards), ring A of **2** adopted a twist boat conformation that allows both hydroxyl groups to interact simultaneously with the carboxylate group of Asp276 (Figure 6, left). Both poses leave the carbonyl group of the steroid exposed to water.

**Figure 7:**
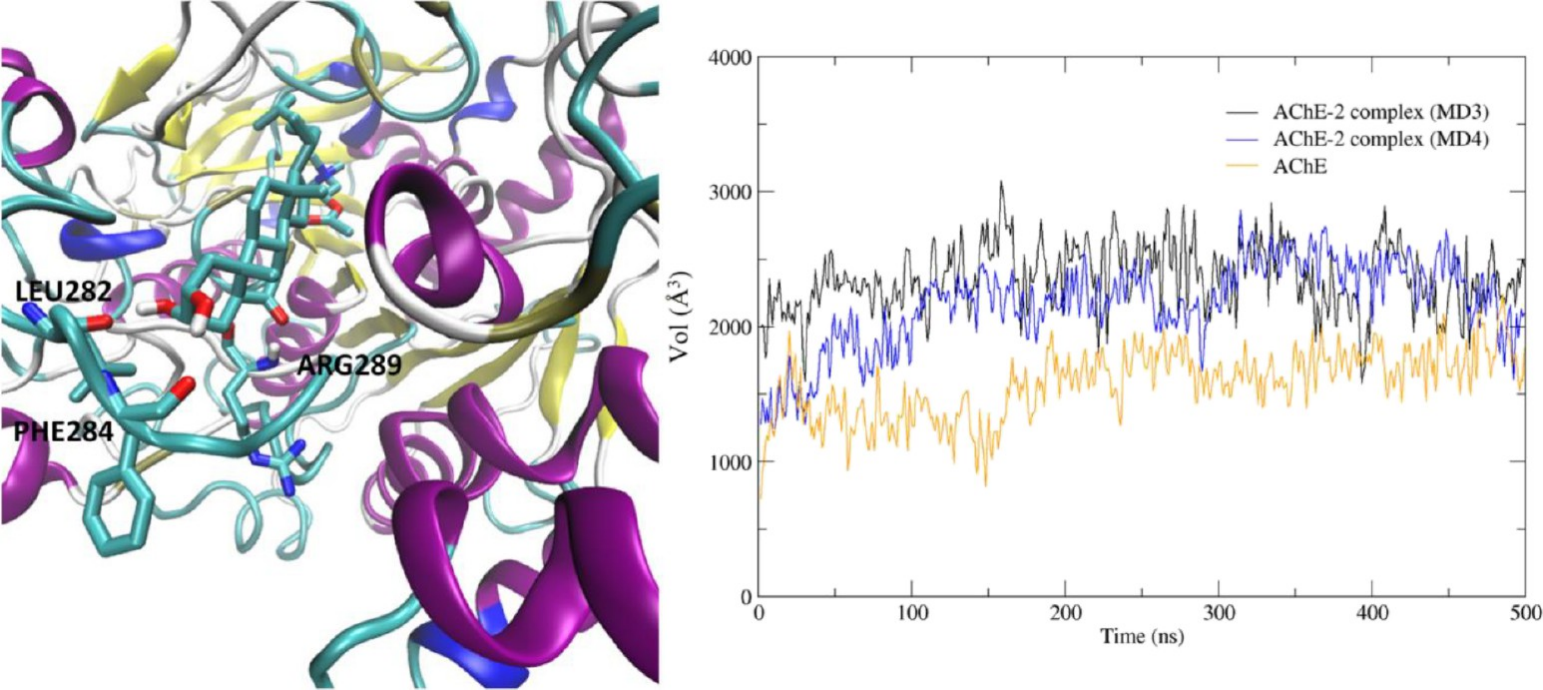
Conformation compound **2** adopted during MD4. Cartoon backbone protein representation colored by the secondary structure (left). AChE pocket volume in the apoenzyme (orange), and in the AChE-2 complex during MD3 (black) and MD4 (blue) (right).

In the MD4 simulation, the steroid fitted into the gorge in a similar manner and remains in a conformation that allowed hydrogen bonding interactions between the hydroxyl group on the C-3 carbon atom and the carbonyl group of Leu282 (65% occupancy, 1.70 Å, 164.6°) or Phe284 (17% occupancy, 1.80 Å, 161.0°) (Figure 7, left and Figure S10, Supplementary Materials). In addition, the hydroxyl group on the C-2 atom of **2** was observed to interact with these residues directly (Phe284 carbonyl 27% occupancy, 1.76 Å, 163.0° or Leu282 carbonyl 20% occupancy, 1.71 Å, 161.6°, Figure S11, Supplementary Materials) or water-mediated. Consequently, the carbonyl group at the C-6 atom was able to interact with the NH in the amide group of Arg289 (62% occupancy, 2.13 Å, 164.1°, Figure S12, Supplementary Materials).

Although **2** also spans the gorge (Figure 7, right), its interactions are strong and stable because it is buried deeper into the PAS between the Ω-loop and helix 13. Moreover, below it will be shown that **2** blocks efficiently the PAS so no tunnel connects the bulk with the active site (Tunnel Analysis section).

Both MD simulations of AChE-**2** showed similar binding free energies along both simulations (−35.20 ± 3.9 kcal/mol for MD3 and −37.66 ± 3.9 kcal/mol for MD4) (Figure S13, Supplementary Materials). It is important to highlight that MM/GBSA binding free energies for compounds **1** and **2** are not comparable since they have different charges.^31,32^

### Tunnel Analysis

#### Tunnel Analysis for Inactive Compound **2** in the AChE-**2** Complex (MD3 and MD4 Simulations)

**Figure 8:**
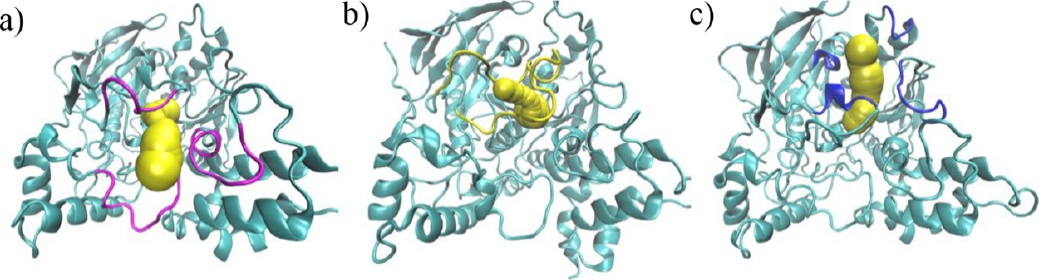
Acetylcholinesterase cartoon representation showing a tunnel connecting the active site with the bulk through (a) main door (PAS); (b) side door (SD); and (c) back door (BD).

Since the inactive dihydroxylated steroid **2** had a stable conformation into the PAS, our first hypothesis was that the enzyme may have its alternative doors opened, explaining the observed activity of the enzyme.^33^ AChE has secondary gates that allow the substrates/products to enter/leave the active site from the solvent bulk, explaining the high catalytic rate of AChE. Two of these gates are the back door (BD) and the side door (SD), their names referring to their position from the main gorge (Figure 8).^34,35^ Long-time MD simulations, 500 ns in our study, allowed us to detect longer time scale protein motions, such as transient doors opening, despite the remarkable protein backbone stability throughout.

**Figure 9:**
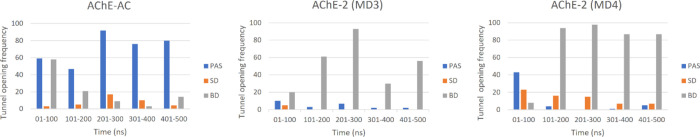
Tunnel opening frequencies of the different doors of the enzyme for AChE–ACh; AChE-**2** (MD3) and AChE-**2** (MD4) determined by Caver. Blue bars correspond to PAS; orange, SD; and gray, BD.

Transient doors opening were confirmed by tunnels detection during the MD trajectory. In order to detect these gates, two computational methods were used: MD Pocket^36^ and Caver^37^ to establish the presence of these transient tunnels that connect the active site with the bulk of the solvent. The Caver program searched for tunnels that connect the active site with the bulk with a radius of, at least, 1.4 Å. This probe size was selected for being the minimal radius of the narrowest region of the pocket, i.e., the bottleneck.^38^Figure 9 reveals the opening frequencies of the three gates in both AChE-**2** simulations compared with that of the AChE–ACh complex. The calculation of the opening frequencies was determined independently for each of the three gates according to the presence/absence of any tunnel per snapshot. In other words, the existence of a tunnel denotes an open channel. The bottleneck average radius distribution found is available in the Supplementary Material (Figures S14–S18).

The main differences observed in Figure 9 between AChE and the AChE-**2** complex are related to the presence of the steroid within the PAS, blocking the main entrance, most of the time. Furthermore, the presence of compound **2** seemed to significantly modify the opening frequency of the back door (BD). Caver analysis showed there is an alternative pathway to the active site through the BD when compound **2** is inside the enzyme, allowing the ACh to move back and forth into the active site. This might explain the activity of the enzyme in the presence of inactive compound **2**.

**Figure 10:**
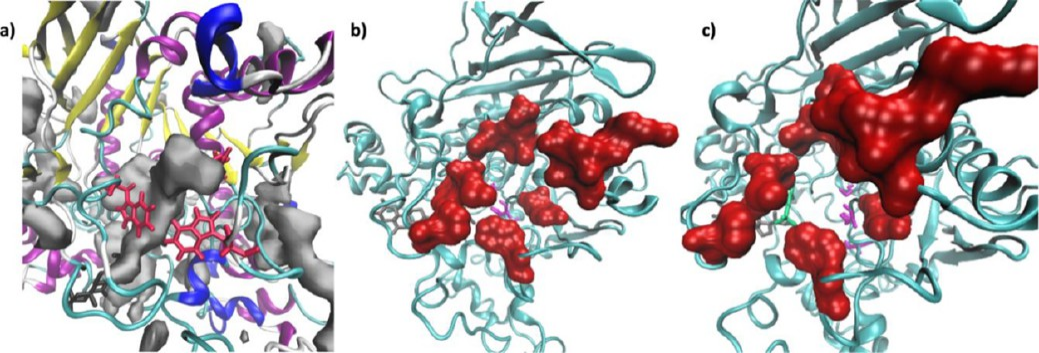
(a) Resultant tunnels (gray surface) observed during AChE-**2** (MD4). Residues Trp84 and Trp114 are represented in red and **2**, in black. Cartoon backbone protein representation is colored by the secondary structure. (b, c) AchE-**2** (MD4), different views. Residues surrounding the BD are represented in red surface; catalytic triad residues, in pink; and ACh is represented in green.

**Figure 11:**
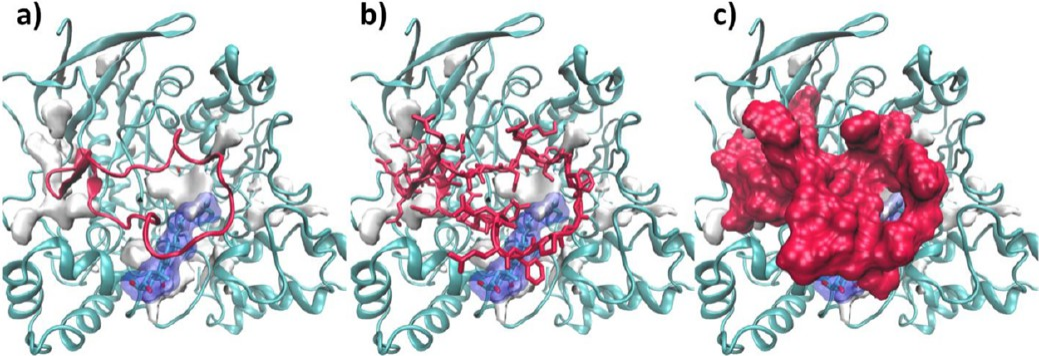
Pocket detections with MDpocket of MD3 simulation of AChE-**2** into the representative conformation of the complex. Compound **2** represented as a blue surface and the side door represented in red in (a) cartoon representation; (b) licorice representation of the residues, and (c) surface representation of the SD.

An MDpocket analysis also supports the occurrence of this transient door at the AChE-**2** complex; Figure 10a clearly shows the tunnel morphology connecting the active site with the exterior even when inactive **2** is located at the PAS (MD4). Indeed, the catalytic triad residues can be observed from the BD entrance, as depicted in Figure 10b,c. For the opening of the BD, a pronounced movement is observed among certain residues of the omega loop, particularly residues 76 to 81 (Figure S19, Supplementary Materials). The molecular process underlying the opening involves the breaking of hydrogen bonds between residues in the omega loop and residues between 427 and 432. The synchrony between these events correlates with the moment when the BD opening is observed in Figure 9. Specifically, the breaking is observed between the indole NH of Trp84 and either the hydroxyl group of Tyr442 or the C=O backbone of Gly80 (Figure S20, Supplementary Materials). Additionally, the interaction between Gly80 (C=O) and Trp432 (indole NH) is also disrupted (Figure S21, Supplementary Materials). This rearrangement leads to Trp84 establishing a new hydrogen bond with the C=O backbone of Pro76 (Figure S20, Supplementary Materials), and Trp432 rearrangement allowed it to continue the hydrogen bond with the hydroxyl group of Tyr442 (Figure S21, Supplementary Materials). This stabilizes the new conformation of the protein with its BD open. These results are in accordance with those reported by Bui et al.^39^ that showed an access to the active site by Ω-loop movement in the presence of the PAS inhibitor Fasciculin-2. Regarding the SD, the blockage of this gate is observed due to the position of the side chain of the steroid **2** during MD3 and MD4 (Figure 11a–c).

#### Tunnel Analysis for Active Compound **1** in the AChE-**1** Complex (MD1 and MD2 Simulations)

**Figure 12:**
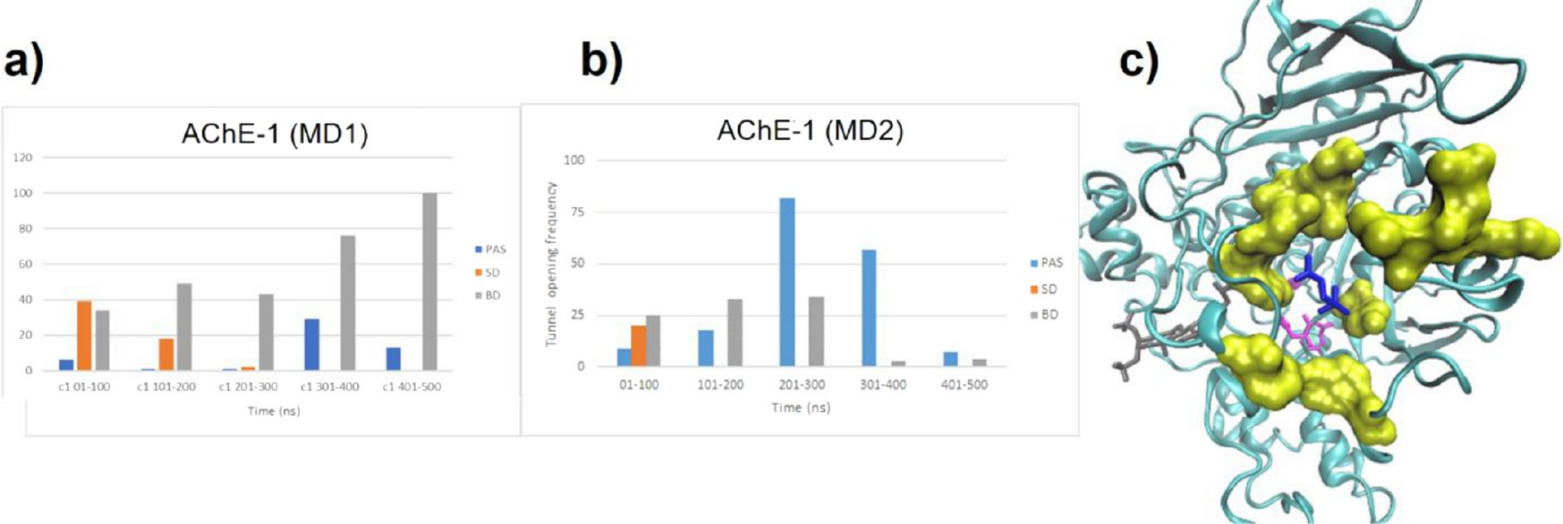
Tunnel opening frequencies of the different doors of the enzyme for (a) AChE-**1** (MD1) and (b) AChE-**1** (MD2) determined by Caver. Blue bars correspond to PAS; orange, SD; and gray, BD. (c) MD snapshot showing the BD opened in the AChE-**1** complex (MD1). Residues surrounding the BD are represented in yellow surface; catalytic triad residues are represented in pink; compound **1**, in gray; and ACh, in blue licorice.

However, these transient doors were also observed in the inhibited AChE-**1** complex (MD1). A clear opening of the SD was observed several times but the side chain of compound **1**, near the Ω-loop, blocked it. In accordance, nearly no tunnels connected the side door with the active site, as shown by the orange bars in Figure 12a,b.

**Figure 13:**
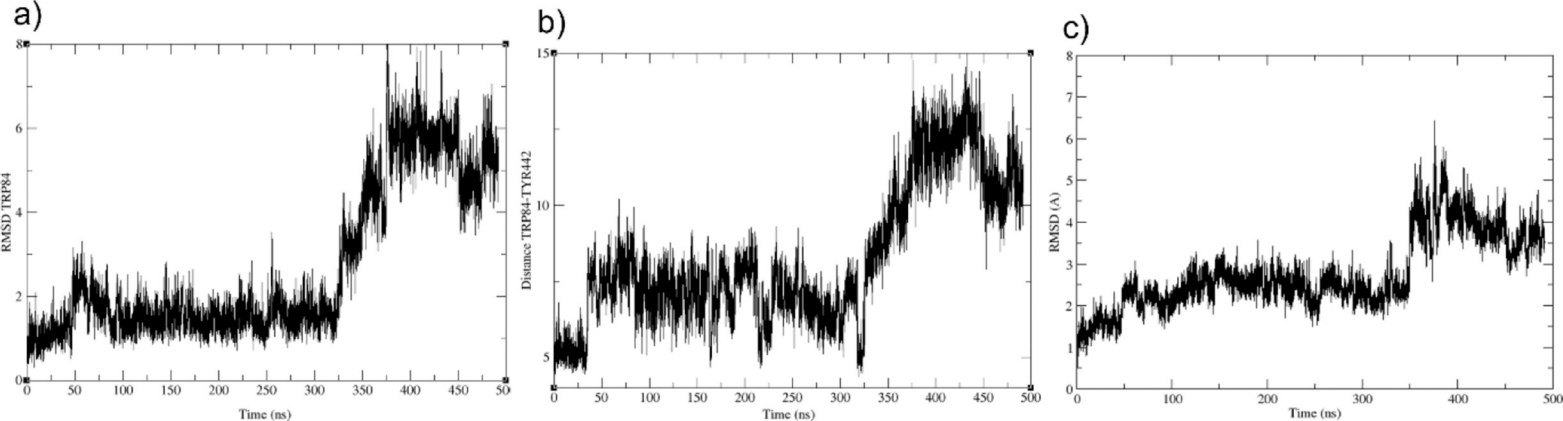
Some relevant RMSD and distance calculations during AChE-**1** (MD1) (a) RMSD representation of the residue Trp84; (b) distance between NH indole of Trp84 and Tyr442 (−OH) and (c) RMSD Ω-loop backbone.

As shown in Figure 12a, the opening of the BD seems to be more significative at longer simulation times during MD1. Furthermore, the MD simulation revealed that, around 350 ns, the ACh began to move out of the active site through the BD (Figure 12c), which had a high opening frequency, according to the Caver results. The BD opening involves the formation of a transient hydrogen bond between the carbonyl group of Asn429 with the hydroxyl group of the Tyr442, which causes a shift of the latter, inducing the rupture of the hydrogen bond between Tyr442 hydroxyl group and Trp84 NH indole (Figure 13). Finally, at 325 ns, Trp84 shifts from Tyr442 allowing the ACh to leave the active site and escape from the AChE assisted by a conformational change in the Ω-loop (Figure 13).^34,38^ This finding demonstrates the possibility of ACh to easily exit through the secondary doors.

**Figure 14:**
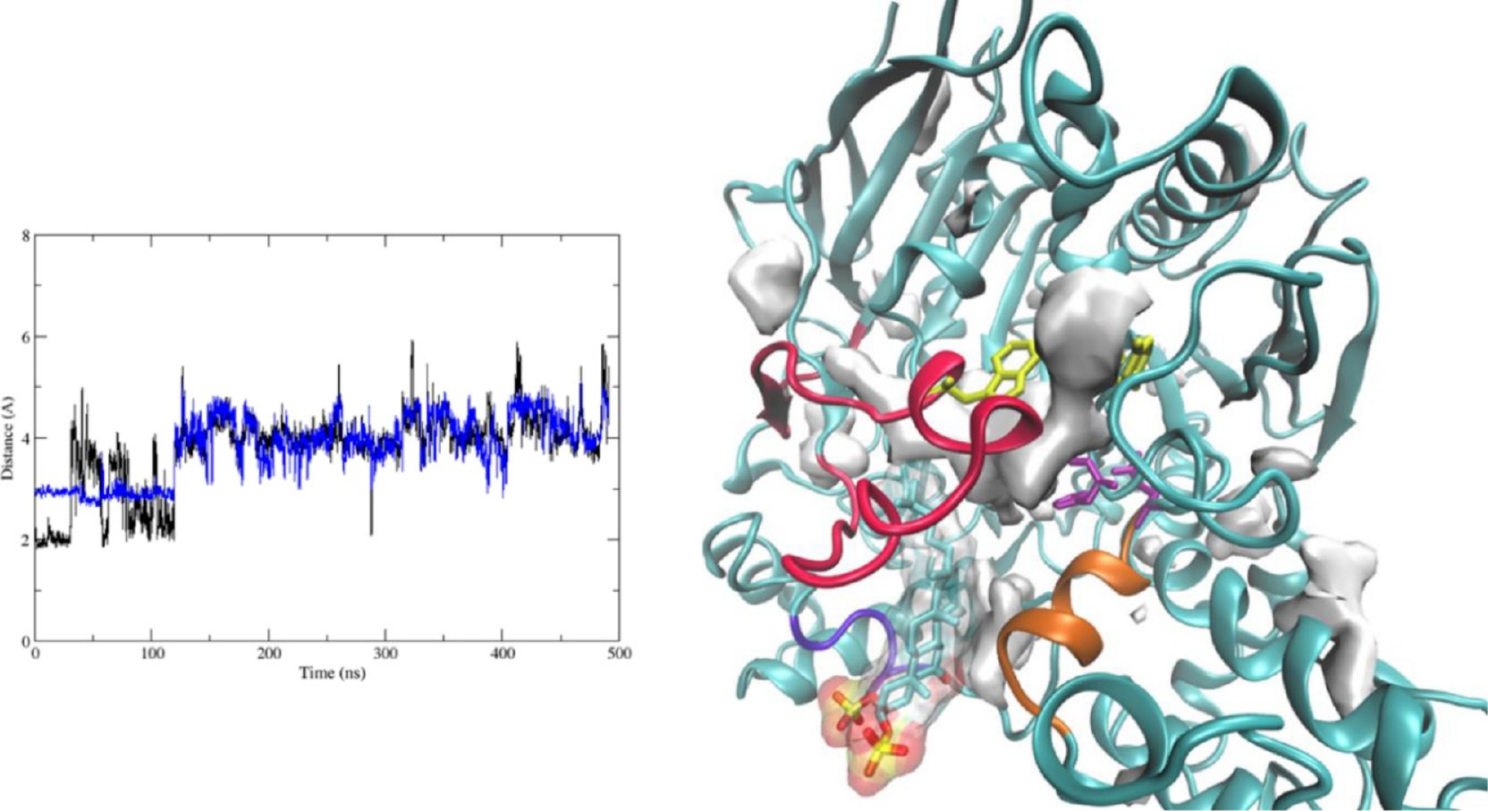
Active site hydrogen bonds during MD1 between −OH of Ser200 and the Nε of His440, in black and carboxylate group (Cδ atom) of Glu327 and the NδH of His440, in blue (left). MDpocket results for the MD1 AChE-**1** complex (right).

The MDpocket tool also supports the occurrence of this transient door (BD) at the AChE-**1** complex (MD1). Figure 14 (right) shows the tunnel morphology (gray surface) that connected the active site with the exterior through the BD.

Although the active site is accessible from the bulk, the catalytic triad was not in a suitable conformation for effective base catalysis (Figure 14, right) as will be explained in Active Site Conformation Analysis section. This simulation shows that simple opening of the secondary gates does not guarantee the activity of the enzyme, but it is also worth checking the conformation of the catalytic triad, linked to allosteric inhibitors.

Regarding MD2, both secondary doors remained mainly closed during the simulation (Figure 12b). However, even in the presence of steroid **1** at the PAS, there is enough space to connect the bulk with the active site through the PAS. This reflected the PAS expansion observed in Figure 3 due to the presence of the steroid into the gorge. This change in the volume of the cavity could explain the high PAS opening frequency observed in the bar graph between the nanosecond 200 to the 400 of the simulation (Figure 12b).

### Umbrella Sampling Calculations

The Caver analysis found tunnels that connect the active site with the bulk with a minimal radius of, at least, 1.4 Å^38,40^ but the ACh radius equals to 2.4 Å,^28^ larger than most average radii observed in the bottleneck radius distribution (Figures S14–S18, Supplementary Materials). In addition, it must be noted that the absence of a certain tunnel does not necessarily point that the ACh cannot cross these gates. That is, it is the enzyme’s flexibility that allows it to adapt to the presence of organic molecules larger than the probe used in Caver’s study. In other words, the tunnel does not need to be fully formed for the ligand to exit the protein. However, it is necessary to calculate the energy related to the escape from the enzyme. Free energy profiles for the exit of the trimethylammonium (TMA), as a ligand model, were obtained using umbrella sampling simulations (energy profiles in Figures S22–S27, Supplementary Materials). Profiles were obtained for the three exit pathways PAS, SD, and BD in the presence of compounds **1** and **2** (Table 1).

**Table 1:** Energy Barrier Obtained for TMA through the Three Exit Pathways (PAS, SD, and BD)

	AChE-**1** (active)	AChE-**2** (inactive)
PAS	5 kcal/mol	2 kcal/mol
SD	5 kcal/mol	1.4 kcal/mol
BD	1.5 kcal/mol	0.8 kcal/mol

The TMA escape-free energies associated to the protein complexed with inactive compound **2** are rather low compared to the values obtained for the AChE-**1** complex. These values show that tunneling is not necessary for the ligand to escape. Even considering that the side chain of **2** effectively blocks the SD and PAS (Figure 11 and Figure 12a,b), a low activation barrier is observed and easy crossing is possible, as the steroid side chain interacts with the protein only through hydrophobic interactions. This is not surprising since compound **2** does not inhibit the enzyme. These results are consistent with the barrier reported for TMA exit when leaving the active site through the PAS in the AChE–ACh complex (between 1.91 and 2.40 kcal/mol) and in agreement with the catalytic rate of the AChE.^27^

Surprisingly, the pathway through BD in the presence of active compound **1** shows a low barrier, consistent with ACh leaving the AChE through the BD in MD1. This observation led us to hypothesize that activity of compound **1** is not based on the blockage of the pathways to the active site but inducing a change in the active site conformation, rendering the enzyme inactive. This suggests that compound **1** would be an allosteric inhibitor. To test this hypothesis, a comprehensive analysis of the conformation of the active site was performed throughout MD simulations.

### Active Site Conformation Analysis

To analyze the allosterism, the hydrogen bond interactions between Glu327 (−COO^–^) and His440 (NHδ) and between Ser200 (−OH) and His440 (Nε) were evaluated. The hydrogen bond between the carboxylate group of Glu327 and the NδH of His440 would enhance the Nε histidine basicity. As a consequence, hydrogen bonding between the Nε of His440 and the hydroxyl group of Ser200 leads to deprotonation of the catalytic serine, enhancing its nucleophilicity.^41^ For this reason, 16 MD simulations of 50 ns each were propagated for each complex (i.e., AChE–ACh, AChE-**1**, and AChE-**2**), starting from the conformations of compounds **1** and **2** obtained from the molecular docking analysis. The results are summarized in Table 2. It is remarkable that, during the MD simulations of the AChE-**1** complex, the active site conformation is changed in almost 70% of the simulations, i.e., ∼30 and 25% more than that observed for AChE–ACh and AChE-**2**, respectively. This study allows us to verify that the active-site conformation is more sensitive to the presence of compound **1** than compound **2**. Both AChE–ACh and AChE-**2** complexes have a similar behavior regarding the active site conformation and the active site tended to conserve the catalytic triad conformation more than in the presence of compound **1**.

**Table 2:** Ratio of MD Simulations with an Inappropriate Active Site Conformation for the Four Complexes^a^^,^^b^

complex	AChE–ACh	AChE–ACh-**1**	AChE–ACh-**2**	AChE-**1**
N*/*N*_tot_	6/16 (37.5%)	11/16 (68.8%)	7/16 (43.7%)	4/16 (25%)

a In parentheses, the ratio is expressed as a percentage.

b N* is the number of simulations with an inappropriate active site conformation. *N*_tot_ is the total number of 50 ns MD simulations.

Since compound **1** is an uncompetitive inhibitor, we studied whether ACh affects the allosterism generated by the steroid. For this purpose, an analysis of the active site conformation was also performed when the disulfated compound **1** was bound to AChE in the absence of the ACh. A 100 ns simulation, starting from the docking conformation previously used, revealed that the complex was stable and that the active site of the enzyme maintained its active conformation during the 100 ns of simulation. The study of cavities indicates that when **1** is inside the PAS of the AChE, the BD presented tunnels that connected it with the active site in 98% of the trajectory. Additional 16 MD simulations of 50 ns each were performed for the AChE-**1** complex in the absence of ACh to verify the active site conformation, starting from the same initial conformation previously used. In this case, 13 of the 16 simulations (≈80%) maintained the optimal arrangement of the catalytic triad residues (Table 2). This result suggests that the presence of the neurotransmitter is necessary for the deformation of the active site and thus for the allosteric action of compound **1**.

## Conclusions

This work reveals that the mechanism of inhibition activity observed for compound **1** must involve allosteric modulation and not a simple blocking of the PAS.^42^ This means that, even though the ligand may promote the opening of secondary gates (as seen in MD1 of the AChE-**1** complex) or there are low barriers to cross them (Table 1), the catalytic triad of the enzyme does not have the active conformation needed to catalyze the hydrolysis reaction. The active site analysis with no ACh at the active site suggests although **1** has affinity for the apoenzyme, it only generates allosteric inhibition when ACh is at the active site. In the absence of the neurotransmitter, such allosterism is not observed, allowing the enzyme to remain functional through its BD. This may explain the uncompetitive mechanism experimentally observed for compound **1** in previous work.^24^

We propose that this allosterism is the key feature that distinguishes the inhibition activity of compound **1** from compound **2**, even though **2** has affinity for the enzyme but not inhibitory activity on AChE. This is not surprising given that the structural differences between both steroids lie in the C-1 and C-2 substitution, which is important for the interaction with PAS entry residues or the solvent, while the identical hydrophobic steroidal skeleton accommodates deeply into the PAS. These two steroids are similarly positioned into the AChE as other sets of steroidal alkaloids^43^ (PAS inhibitors) where the high hydrophobicity of these compounds stabilized the inhibitor–enzyme complexes, highlighting the hydrophobic interactions observed at the peripheral site.

These results suggest that the mere presence of ligands that bind and block the PAS does not guarantee that they inhibit the AChE catalytic activity. There is evidence of ligands that exhibit affinity for AChE and occupy the PAS but without affecting its catalytic activity. For example, Barak et al. reported that a PAS inhibitor lost its inhibitory activity but with no loss of affinity when bound to the mutant enzymes W86A and Y133A.^42^ Another example showed that the AChE enzyme in the presence of the peptide ligand Fab410 at the PAS modulated the opening of a secondary gate connecting the outside of the enzyme to the active site, explaining the observed remaining activity.^44^ It was also shown that the AChE–Fasciculin complex presents a pathway to the active site, by movement of the Ω-loop, which would explain the remaining activity observed in the enzyme.^39^

This work not only brings to light the molecular basis of the mechanism by which compound **1** inhibits but also provides a methodology when screening compounds located at the PAS. Molecular docking is a helpful and a widely used tool in searching chemical libraries. It is frequent that the most promising compounds after a virtual screening (including molecular docking, pharmacophore-based screening, among others) are prone to synthesize or purchase with no further molecular modeling analysis.^45,46^ This study highlights the importance of long MD simulations of enzyme–ligand complexes following molecular docking calculations, for those potential PAS inhibitors. Although the molecular docking indicates affinity toward the enzyme, it is essential to analyze not only the complex stability but also the conformation of the active site, in order to verify allosterism. Moreover, in those cases where the active site has the proper conformation and it is suspected that the ligand inhibits by obstruction of the PAS, analysis of the secondary doors opening plays a relevant role (open/close frequency and the energetic cost to cross them). Our case evidences this problem: since every AChE-**2** simulation showed complex stability, along with other known polyhydroxylated AChE inhibitors,^47−49^ this could lead to the erroneous conclusion that this compound may inhibit the enzyme. However, the careful analysis revealed that inactive compound **2** seems to encourage secondary doors of access to the enzyme’s active site. This may not be a problem for catalytic anionic site (CAS) inhibitors or dual-site inhibitors since the active site is occupied, but PAS-ligands should be carefully examined before synthesis or purchase. Particular attention has been given to PAS inhibitors since AChE catalyzes the conformational change of the amyloid peptide (α-helix) to β-sheets through the interaction of the peptide with part of the AChE PAS, accelerating the β-amyloid plaque formation.^50−54^ This leads to the design and development of dual-acting ligands.^55^ This is the main advantage of PAS inhibitors: they may modulate this non-cholinergic function of AChE linked to the process of amylogenesis by interfering in the interaction with amyloid β-peptide.^56^ Therefore, these inhibitors would not only reduce the cognitive symptoms but would also reduce the formation of β-amyloid plaques linked to synaptic dysfunction, inflammation, neuronal death, and, eventually, dementia.^57^

## Materials and Methods

### Molecular Dynamics Simulations

MD simulations were performed starting from the crystal structure of *Torpedo californica* AChE complexed with Xe, solved at 2.3 Å resolution (PDB entry: 3M3D(58)). The xenon atom was removed and a molecule of ACh was placed at the active site by superimposing the apo-3M3D and 2ACE proteins active sites. The protein–ligand systems starting structures were obtained from the molecular docking results obtained in a previous work.^24^

All the MD simulations were performed with AMBER 16 software package.^59^ The systems were immersed in an octahedral box of TIP3P water molecules^60^ using the tleap module. The minimum distance between protein and the boundary of the box was 10 Å. All systems were simulated employing periodic boundary conditions and Ewald sums for treating long-range electrostatic interactions.^61^ SHAKE was used to keep bonds involving hydrogen atoms at their equilibrium length.^62^ This allowed us to employ a 2 fs time step for the integration of Newton’s equations. The Amber 99 force field, General AMBER force field, and TIP3P implemented in AMBER were used to describe the protein, ligands, and water, respectively.^63^ The temperature was regulated with the Berendsen thermostat, and pressure with the barostat, as implemented in AMBER. All systems were first optimized to minimize any possible structural clashes. Subsequently, the systems were heated slowly from 0 to 300 K using a time step of 0.002 ps, under constant volume conditions. Finally, a short simulation at a constant temperature of 300 K and constant pressure of 1 bar was performed using a time step of 0.002 ps, to allow the systems to reach a stable density. These equilibrated structures were the starting point for production MD simulations.

During the MD analysis, hydrogen bond parameters were calculated with the auxiliar program CPPTRAJ^64^ (AmberTools18 package). The threshold values (distances and angles) used for H-bond interactions were a maximum distance of 2.5 Å from the hydrogen atom to the heteroatom and a minimum angle of 135°.

### Analysis of Tunnels

The program CAVER 3.0 was chosen for this analysis. Snapshots were taken at 1 ns intervals along the MD trajectories, generating a total number of 500 snapshots of the systems for the tunnel analysis. The ACh was previously removed from the active site to find the tunnels. The probe was placed at the active site of the enzyme. The radius for the probe was set as 1.4 Å.^38^ The clustering threshold was set at a value of 3.5 Å but, due to the closer positions of the doors, careful analysis of the tunnels presented in each snapshot was done to avoid tunnels overestimation or misallocated. For bottleneck average radius distribution, all tunnels were considered. The lowest bottleneck radii are equal to the size of the probe (1.4 Å). Other default parameters were used as listed in the CAVER user guide version 3.0.

The program MDpocket allows us to get pocket descriptors from the MD trajectory (i.e., pocket volume) and the images of pocket occurrences. Protein conformations (500; i.e., 1 snapshot/ns) were subject to tunnel analysis from each MD simulation. All pockets represented in the figures have been delimited using a 0.32 isovalue. This unit is related to the *N*° of Voronoi Vertices inside a cube (8 Å^3^) around each grid point in every snapshot. In addition, this value was taken to calculate the pocket volumes.

### Umbrella Sampling Calculations

Umbrella sampling calculations were carried out using AMBER 16^60^ in order to obtain the energy profiles for the exit of model compound TMA through pathways PAS, SD, and BD. The umbrella sampling calculations were performed by fixing the distance between TMA and residue Gly441 from 5.09 to 24.56 Å, with an interval of 0.33 Å and a force constant of 5 kcal/(mol Å^2^). Results were analyzed and extra windows with intermediate values were added whenever necessary. Each window was simulated for 6 ns, and the last 5 ns were included for the energy calculation. The weighted histogram analysis method^65^ was used in order to construct the free energy profiles. Independent calculations were run for the protein bound to compounds **1** and **2**.

### Analysis of Active Site Conformation

This analysis was performed by plotting the distances between Glu327 (−COO−) and His440 (NHδ) and between Ser200 (−OH) and His440 (Nε) in each simulation. The threshold values used for analyzing active site conformation were ∼3.5 and 2.5 Å, respectively. Confirmation of active site rearrangement was determined by clearly marked jump in the graphic (Figure S28, Supplementary Materials).

### MM/GBSA Calculation

This method evaluates the binding free energy of the enzyme–ligand complex in solution. Since water–water interaction contributions would be much larger than those of the protein–ligand complex; this method employs a thermodynamic cycle to speed up the binding energy calculation, rendering this equation:

Δ*G*°_bind,solv_ = Δ*G*°_bind,vacuum_ + Δ*G*°_solv complex_ – (Δ*G*°_solv,lig_ + Δ*G*°_solv. prot_); where *G* is calculated as following:

*G* = *E*_bind_ + *E*_electrost_ + *E*_vdW_ + *G*_pol_ + *G*_np_ – *TS*where *E*_bind_ + *E*_electrost_ + *E*_vdW_ are the standard molecular mechanics terms and *G*_pol_ and *G*_np_ are the polar and non-polar contributions to the solvation-free energies, respectively. *G*_pol_ is obtained using the generalized Born method, and *G*_np_ is estimated from the linear relationship with solvent-accessible surface area. Entropy—computationally expensive to calculate—, in this case, was not calculated as similar entropy states were compared.

The binding free energy of the enzyme–ligand complex in solution was calculated along the MD simulation analyzing 25 snapshots/ns (12,500 snapshots from each 500 ns MD simulation) with the MMPBSA.py,^66^ part of the open source AmberTools package.

## Data Availability

The inputs used in this work, parameters files, as well as the starting structures, are available at https://github.com/parroyoUV/ache_steroids. For more information, please contact the corresponding authors, who will contact you. All production trajectories generated in this study can be requested from the corresponding authors. Software used: Amber 16, http://ambermd.org/AmberMD.php; Ambertools 16, http://ambermd.org/AmberTools.php; Caver 3.0, https://caver.cz/; fpocket 1.0, https://fpocket.sourceforge.net/; and VMD 1.9.3, http://www.ks.uiuc.edu/Research/vmd/.

## Supporting Information Available

AChE bottleneck movement, additional RMSD calculations, distances related to hydrogen bonds, bottleneck average radii distribution, the free energy profile along distance (umbrella sampling), and active site conformation analysis (PDF)

## Abbreviations

ACh: acetylcholine
AChE: acetylcholinesterase
AChEI: acetylcholinesterase inhibitor
AD: Alzheimer’s disease
Aβ: amyloid β
BD: back door
CAS: catalytic active site
MD: molecular dynamics
PAS: peripheral anionic site
RMSD: root-mean-square deviation
SD: side door
TMA: tetramethylammonium
