# Supplementary material for: Putting the Puzzle Together To Get the Whole Picture: Molecular Basis of the Affinity of Two Steroid Derivatives to Acetylcholinesterase
Source: ACS Omega. 2023 Jul 6;8(28):25610–22. doi: 10.1021/acsomega.3c03749 (PMC10357547; doi:10.1021/acsomega.3c03749)
Supplement: Supplementary file 1 — ao3c03749_si_001.pdf [file ao3c03749_si_001.pdf]

Putting the puzzle together to get the whole picture: Molecular basis of the affinity of two steroid derivatives to acetylcholinesterase

Victoria Richmond[1], Bruno N. Falcone[1†], Marta S. Maier[1], Pau Arroyo Máñez[2]**

[1]Facultad de Ciencias Exactas y Naturales, Departamento de Química Orgánica, Universidad de Buenos Aires, Buenos Aires, C1428EGA, Argentina y CONICET-Universidad de Buenos Aires, Unidad de Microanálisis y Métodos Físicos aplicados a la Química Orgánica (UMYMFOR), Buenos Aires, Argentina

[2]Instituto Interuniversitario de Investigación de Reconocimiento Molecular y Desarrollo Tecnológico (IDM), Universitat Politècnica de València, Universitat de València, Valencia, 46100, Spain and Departamento de Química Orgánica, Universitat de València, Valencia, 46100, Spain

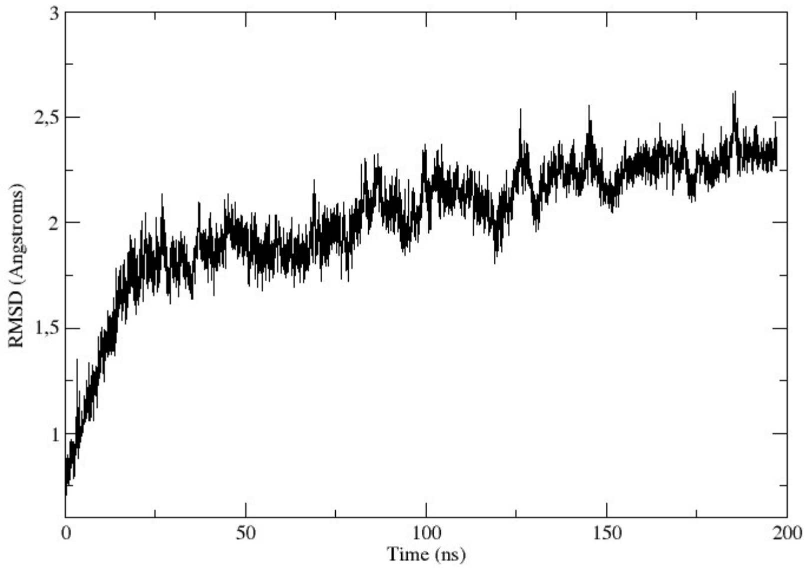

Figure S1 - RMSD of the protein backbone AChE

[†] † Current address: School of Chemistry, University of Nottingham, Nottingham, UK.

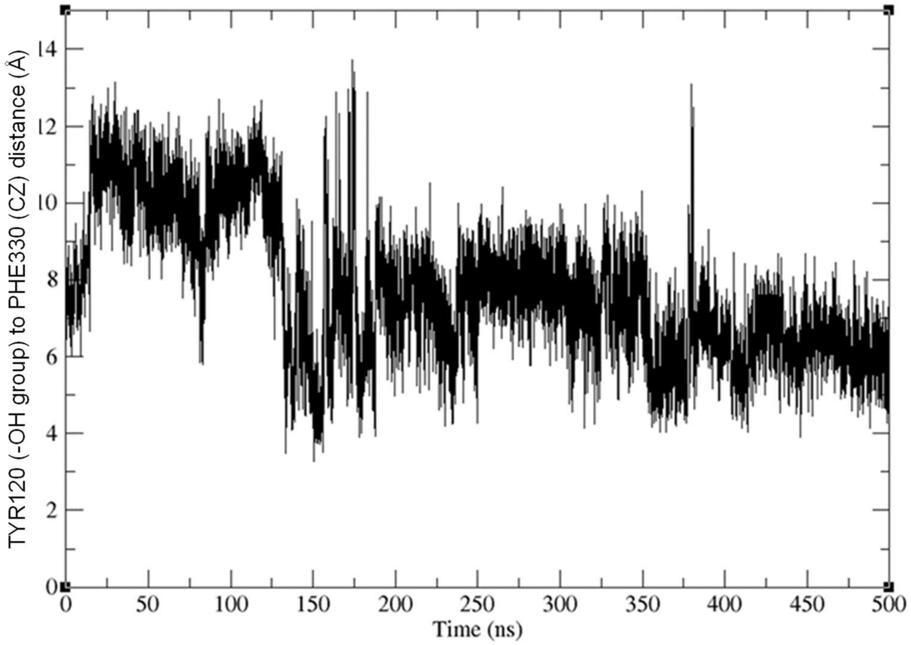

Figure S2 – AChE bottleneck movement represented by the distance between CZ of Phe330 and the oxygen phenol of Tyr120

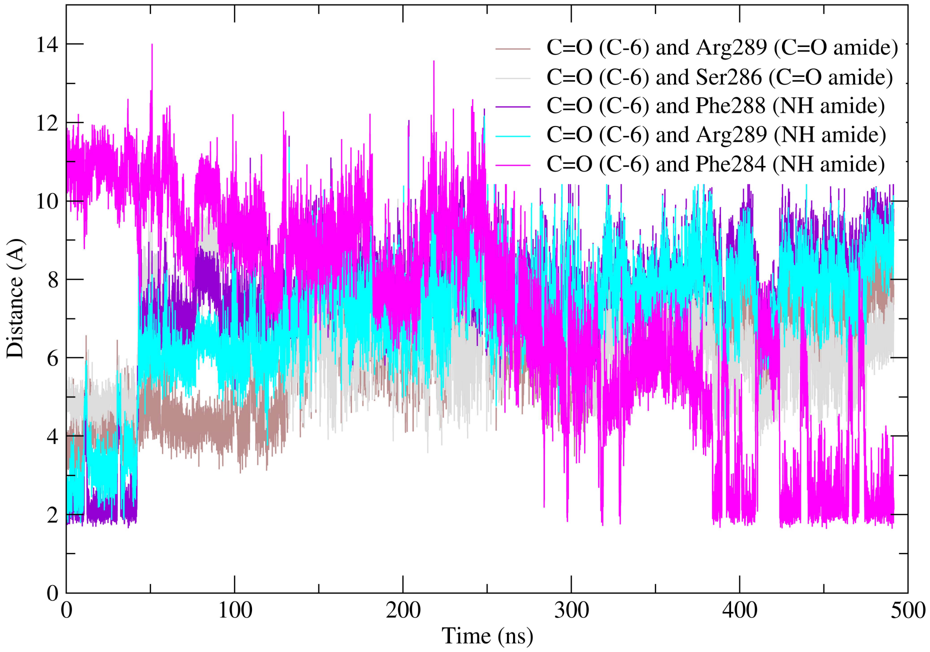

Figure S3 – Water mediated or direct H-bond involving Compound 1 (C=O) during MD1.

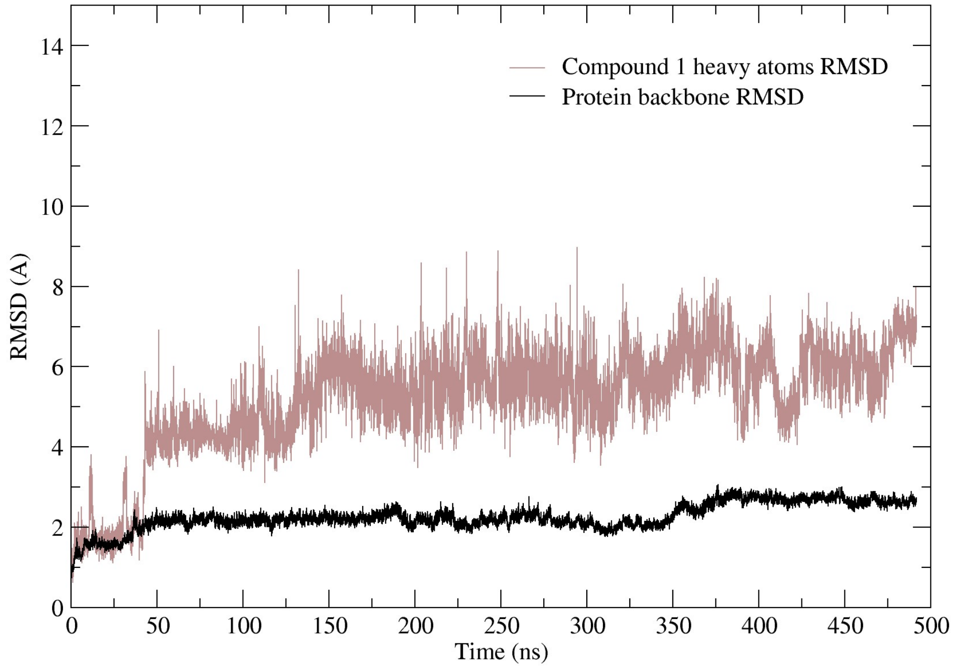

Figure S4 – RMSD of the protein backbone (black) and RMSD of heavy atoms of 1 (brown) during MD1.

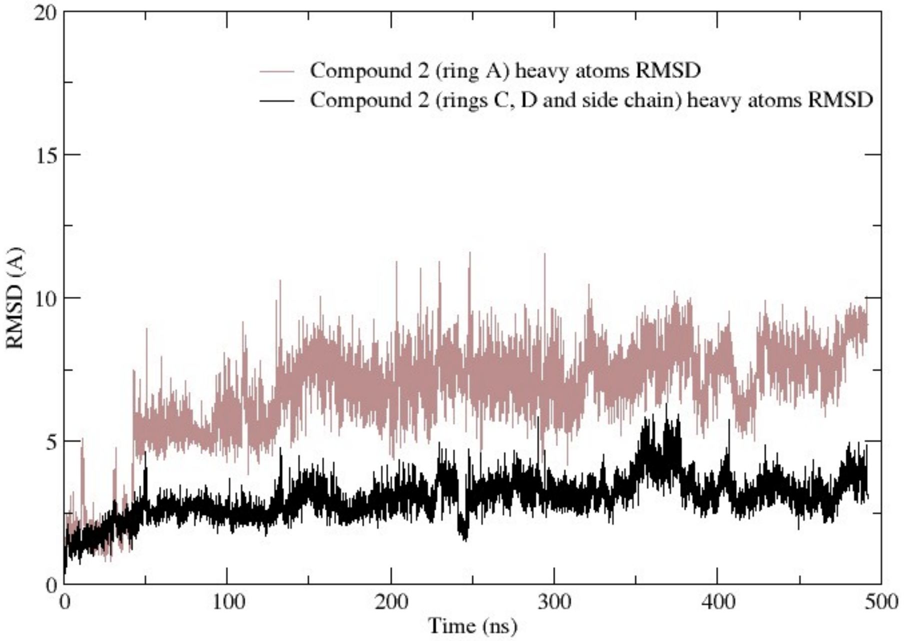

Figure S5 – RMSD of ring A heavy atoms (black) and rings C and D heavy atoms (brown) of 1 during MD1.

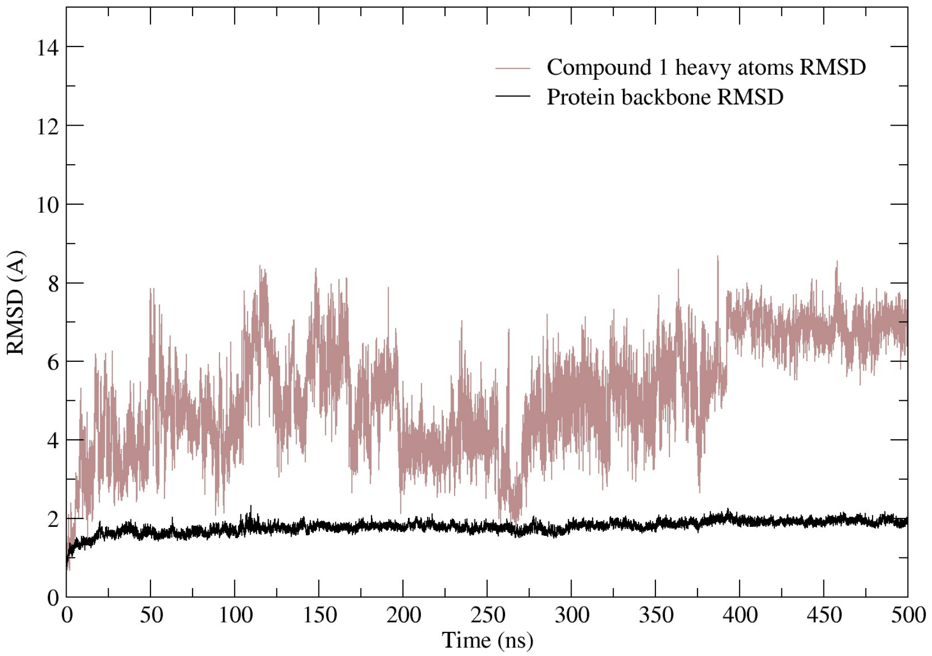

Figure S6 – RMSD of the protein backbone (black) and RMSD of heavy atoms of 1 (brown) during MD2.

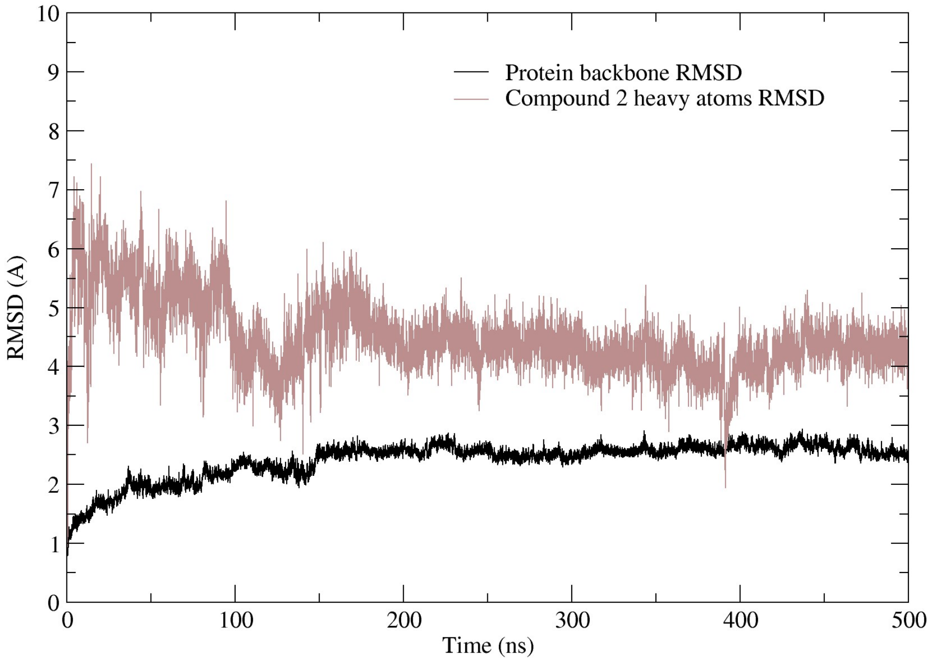

Figure S7 – RMSD of the protein backbone (black) and RMSD of heavy atoms of 2 (brown) during MD3.

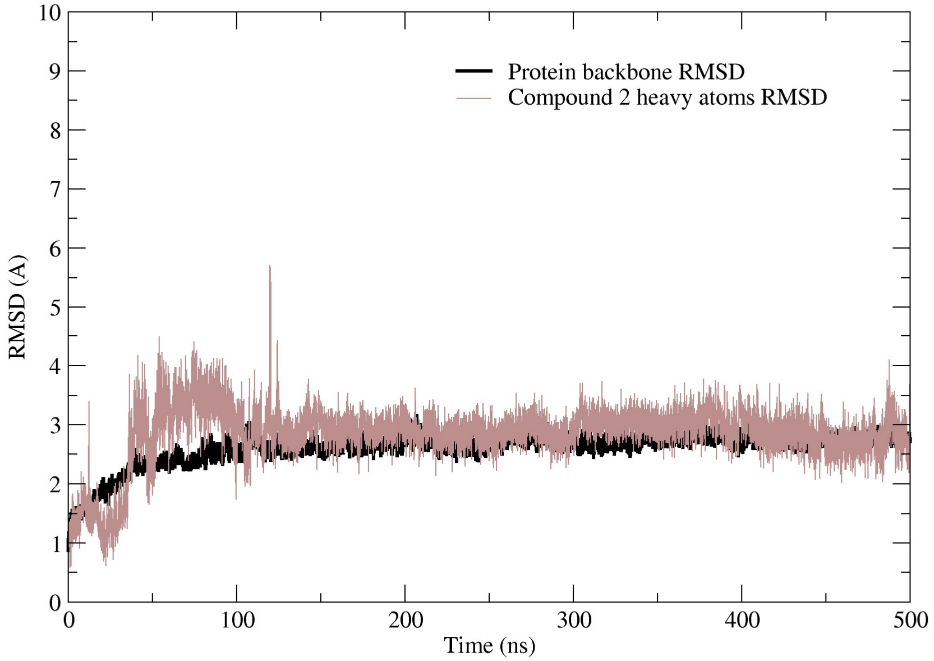

Figure S8 – RMSD of the protein backbone (black) and RMSD of heavy atoms of 2 (brown) during MD4.

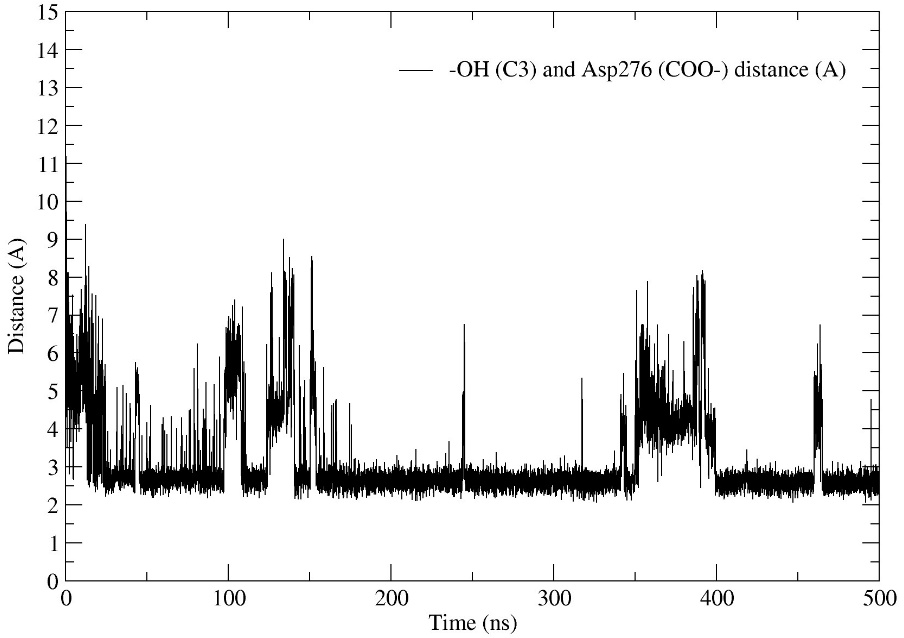

Figure S9 – –OH (C-3) and Asp276 (–COO-) distance during AChE-2 (MD3)

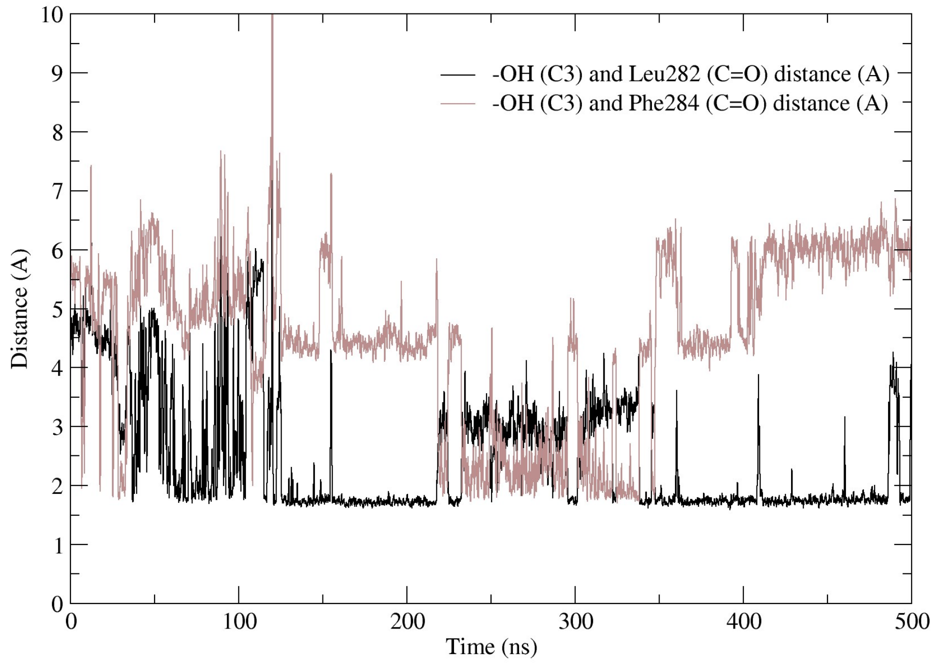

Figure S10 – –OH (C-3) and Leu282 (C=O) and –OH (C-3) and Phe284 (C=O) distance during AChE-2 (MD4)

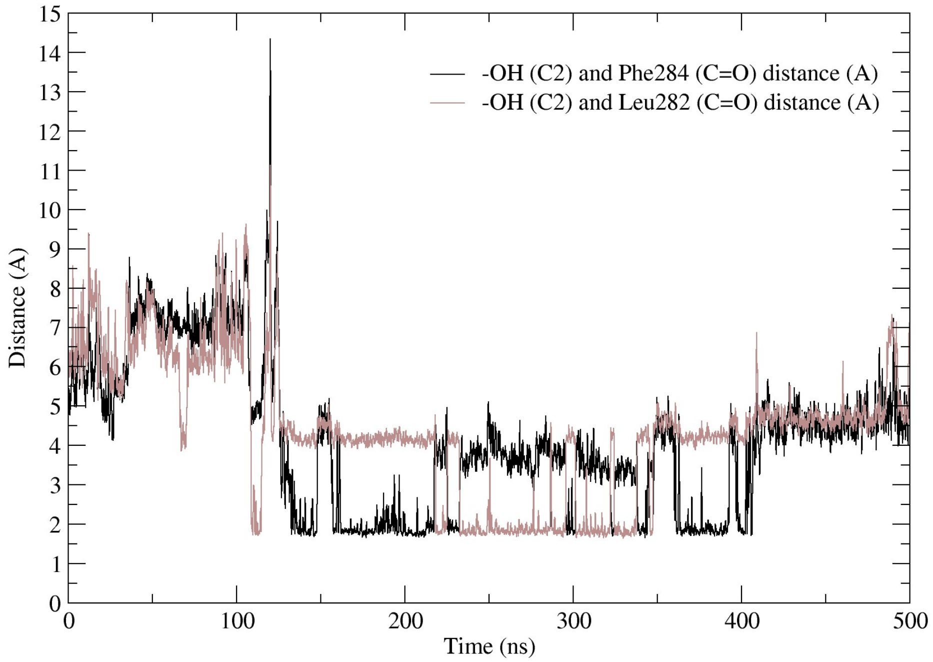

Figure S11 – –OH (C-2) and Phe284 (C=O) and –OH (C-2) and Leu282 (C=O) distance during AChE-2 (MD4)

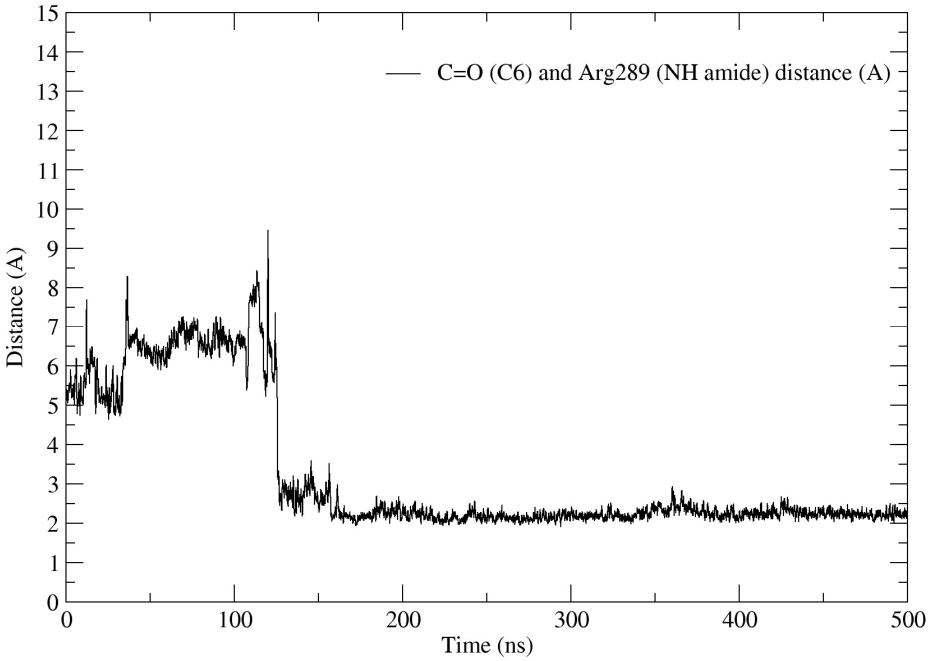

Figure S12 – C=O (C-6) and Arg289 (NH amide) distance during AChE-2 (MD4)

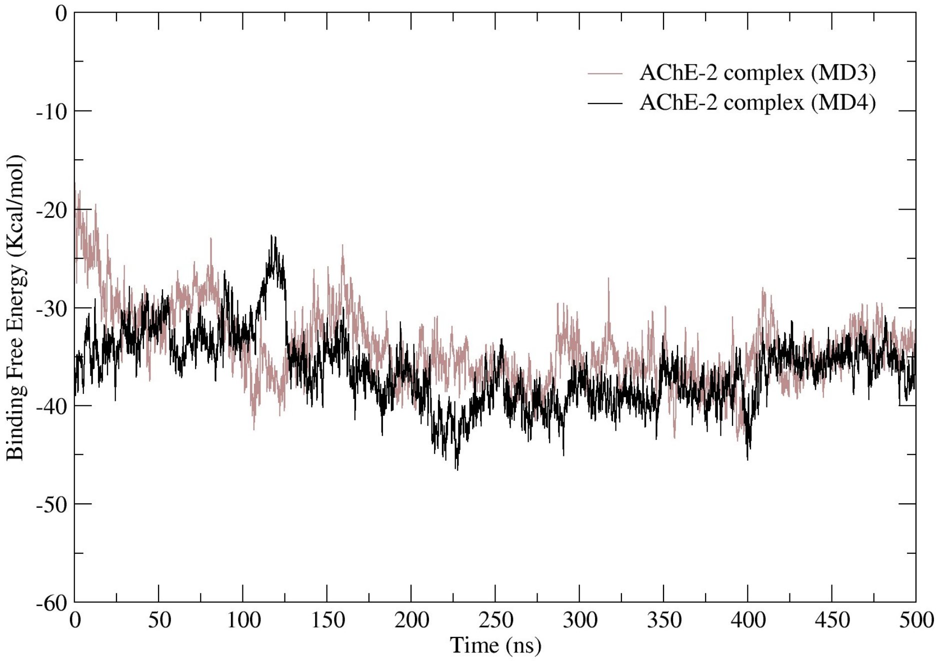

Figure S13 – MM-GBSA free energy graphic for the complex AChE-2. MD3 is represented in red and MD4, in black.

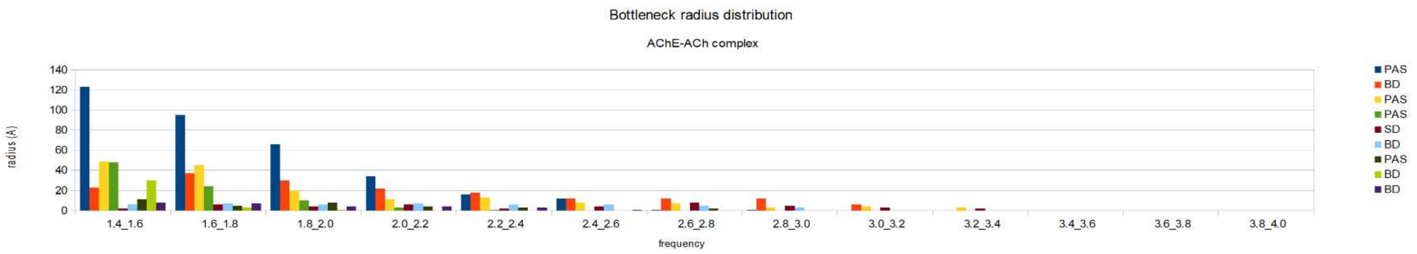

Figure S14 – Bottleneck radius distribution of the tunnels found in AChE-ACh complex (Caver).

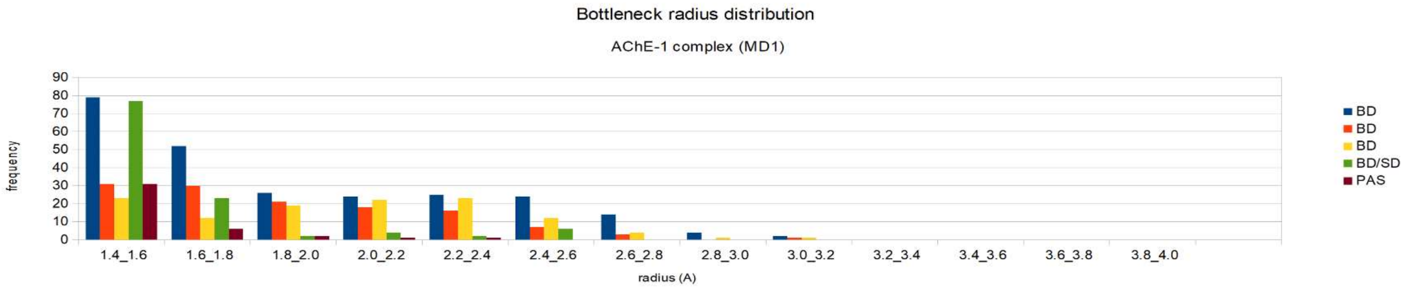

Figure S15 – Bottleneck radius distribution of the tunnels found in AChE-1 (MD1) complex (Caver).

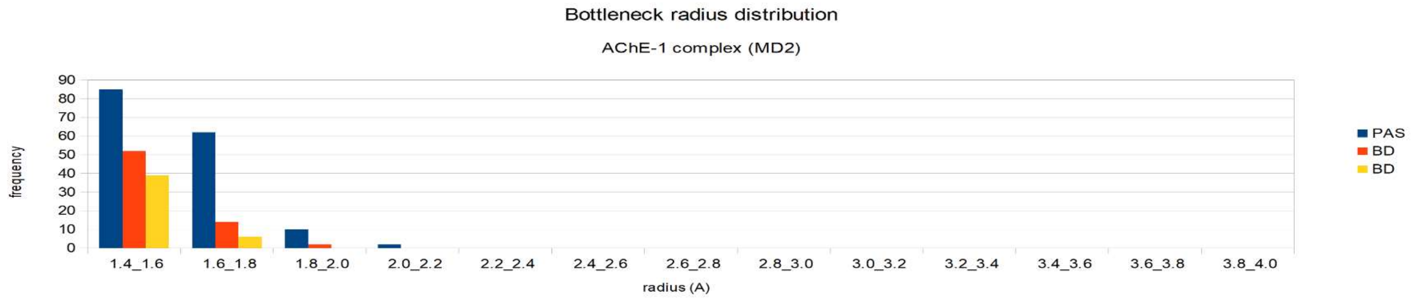

Figure S16 – Bottleneck radius distribution of the tunnels found in AChE-1 (MD2) complex (Caver).

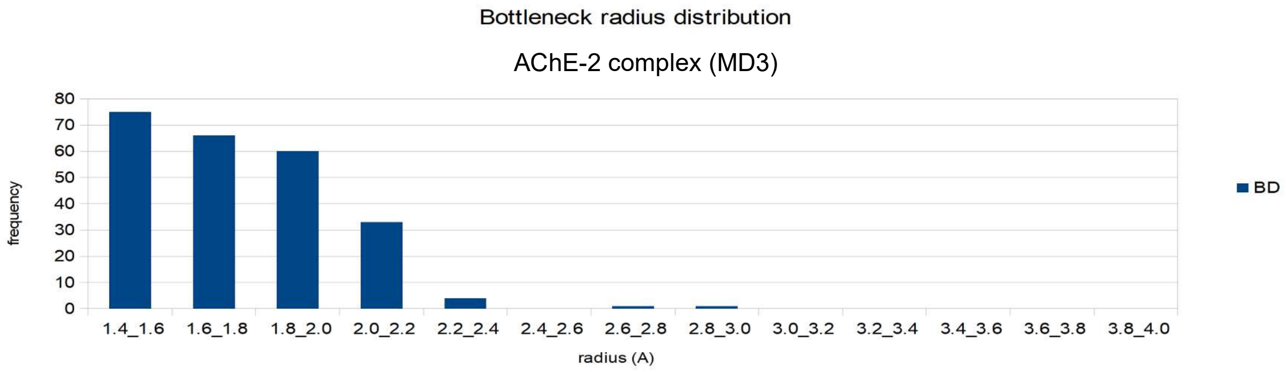

Figure S17 – Bottleneck radius distribution of the tunnels found in AChE-2 (MD3) complex (Caver).

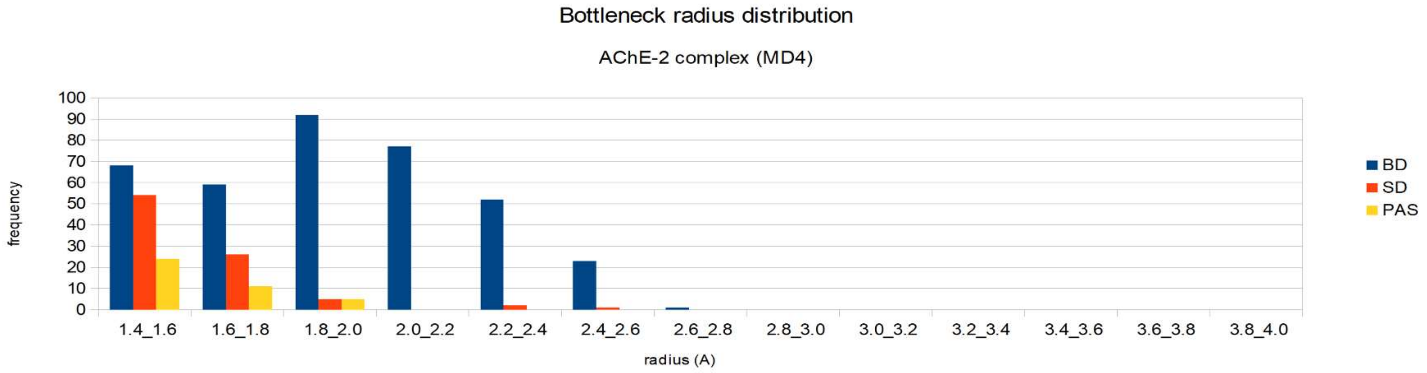

Figure S18 – Bottleneck radius distribution of the tunnels found in AChE-2 (MD4) complex (Caver).

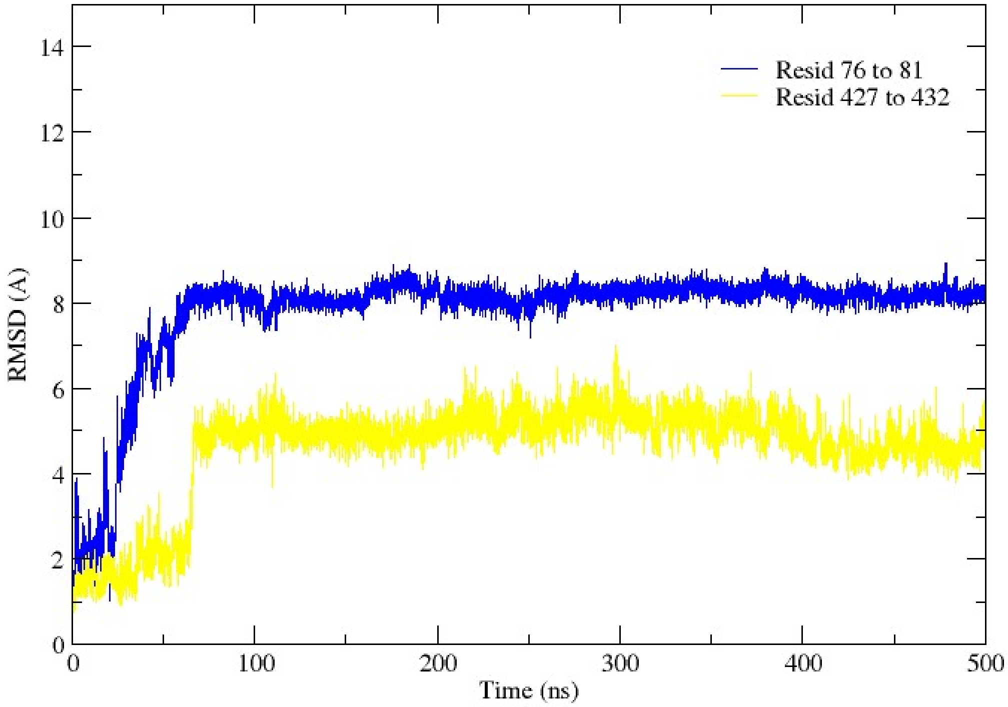

Figure S1**9 –** RMSD of heavy atoms of resids 76 to 81 (part of Ω-loop) and resid 427 to 432 during AChE-2 simulation (MD4).

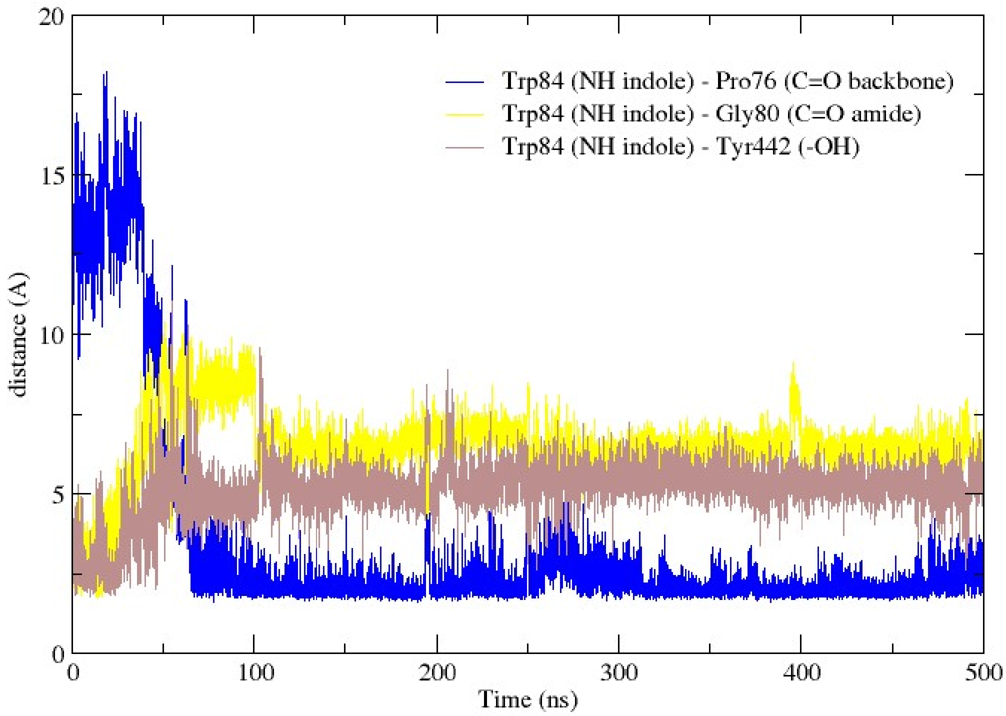

Figure S20 – Hydrogen bonds involved during BD opening in AChE-2 simulation (MD4)

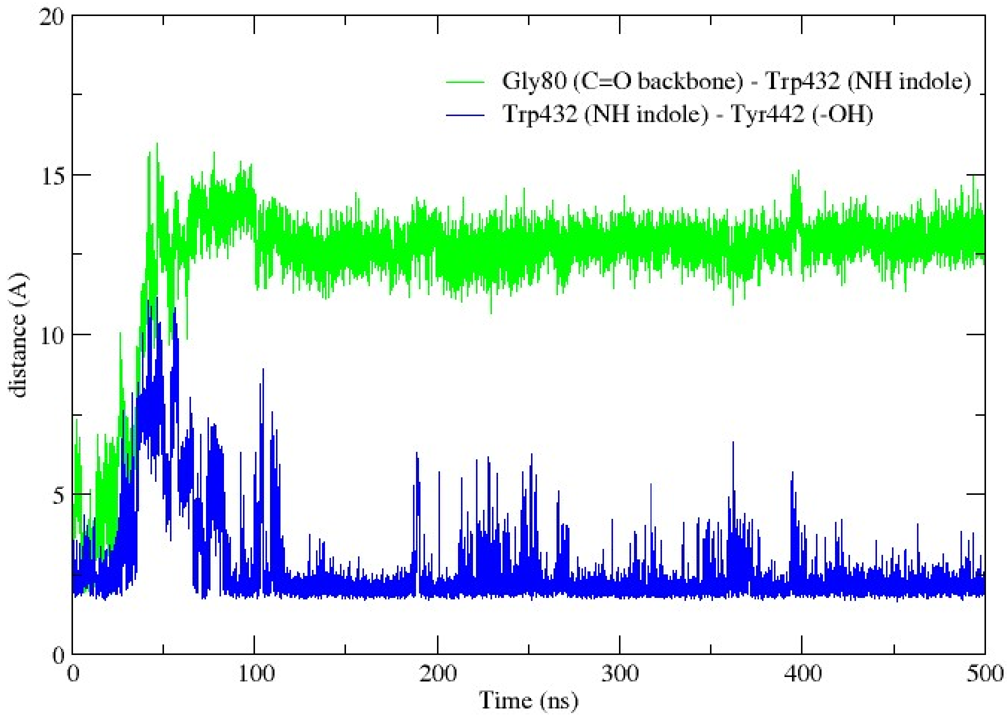

Figure S21 – Hydrogen bonds involved during BD opening in AChE-2 simulation (MD4)

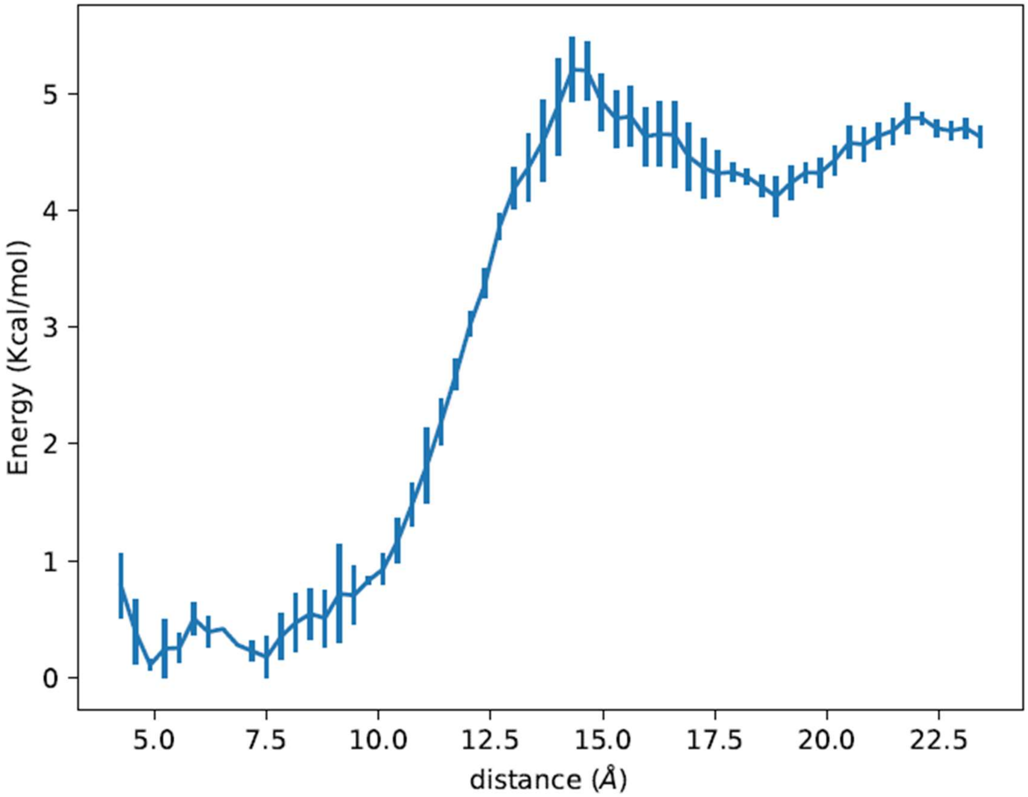

Figure S22 – Energy barrier of TMA in AChE-1 complex to cross the PAS.

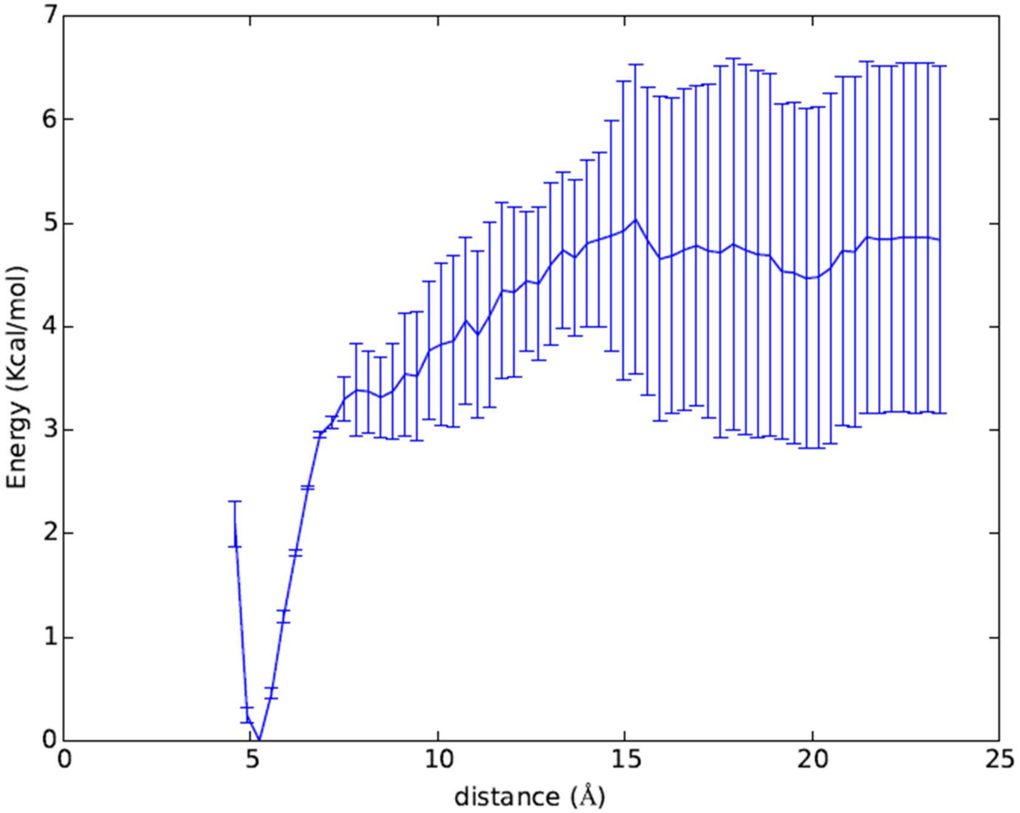

Figure S23 – Energy barrier of TMA in AChE-1 complex to cross the SD.

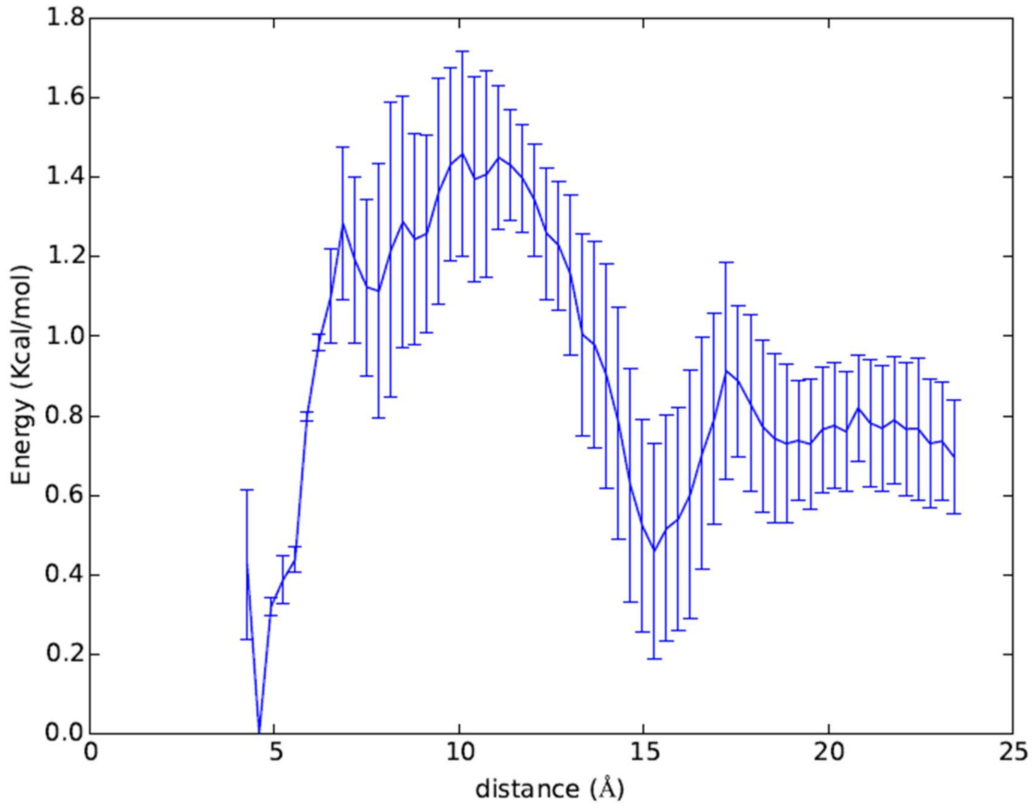

Figure S24 – Energy barrier of TMA in AChE-1 complex to cross the BD.

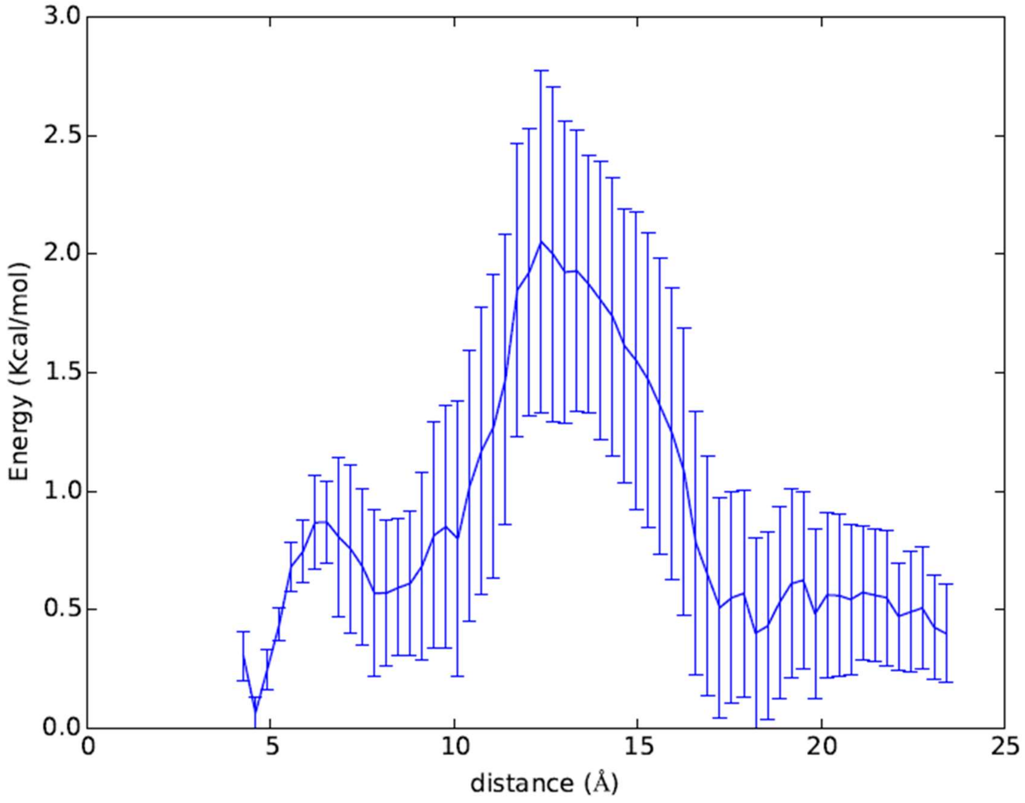

Figure S25 – Energy barrier of TMA in AChE-2 complex to cross the PAS.

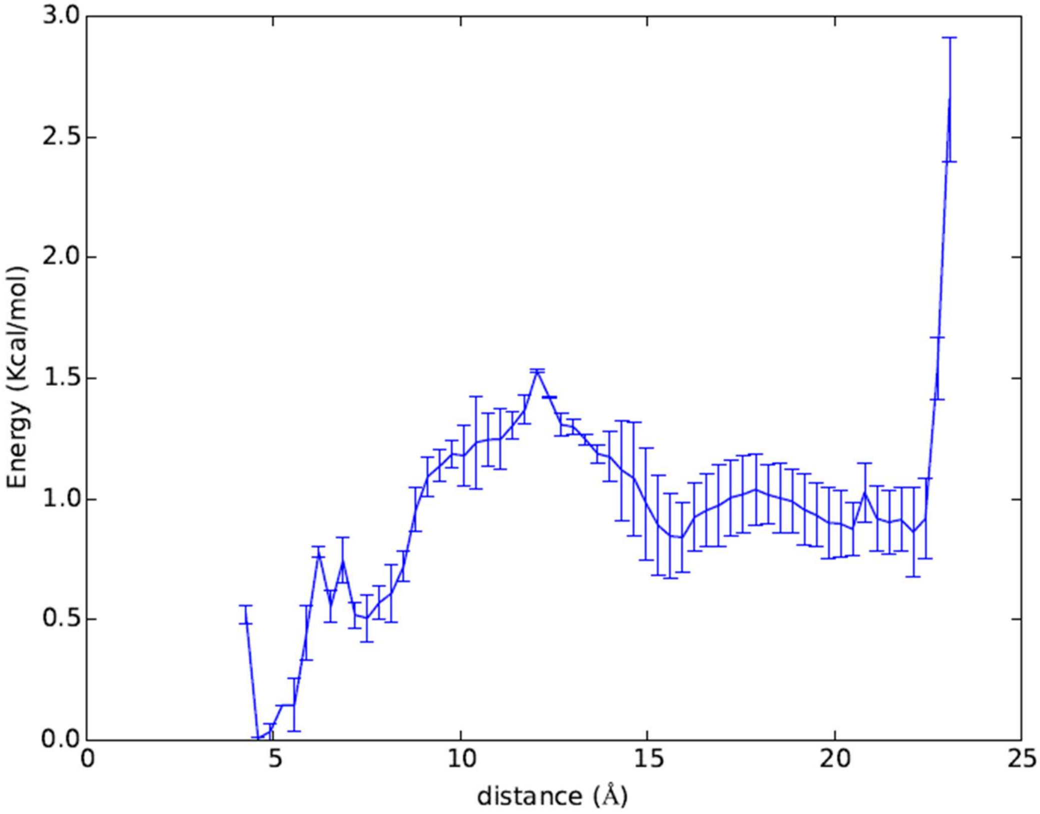

Figure S26 – Energy barrier of TMA in AChE-2 complex to cross the SD.

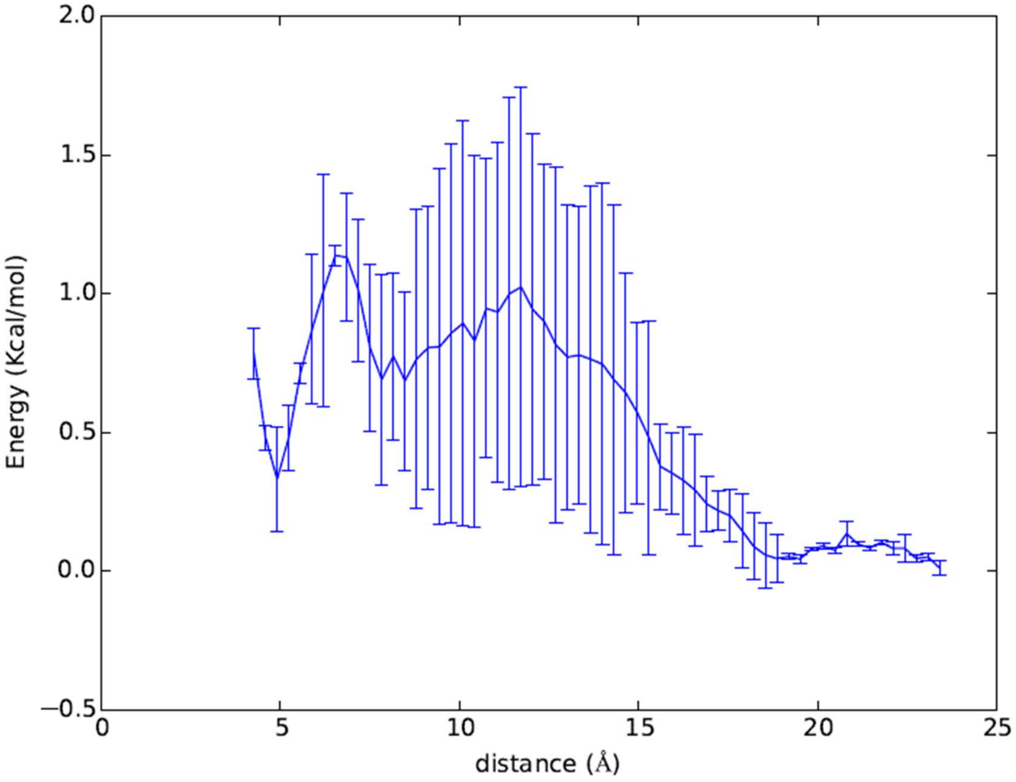

Figure S27 – Energy barrier of TMA in AChE-2 complex to cross the BD.

Figure S28 – Active site analysis of the different complexes.

AChE-AC

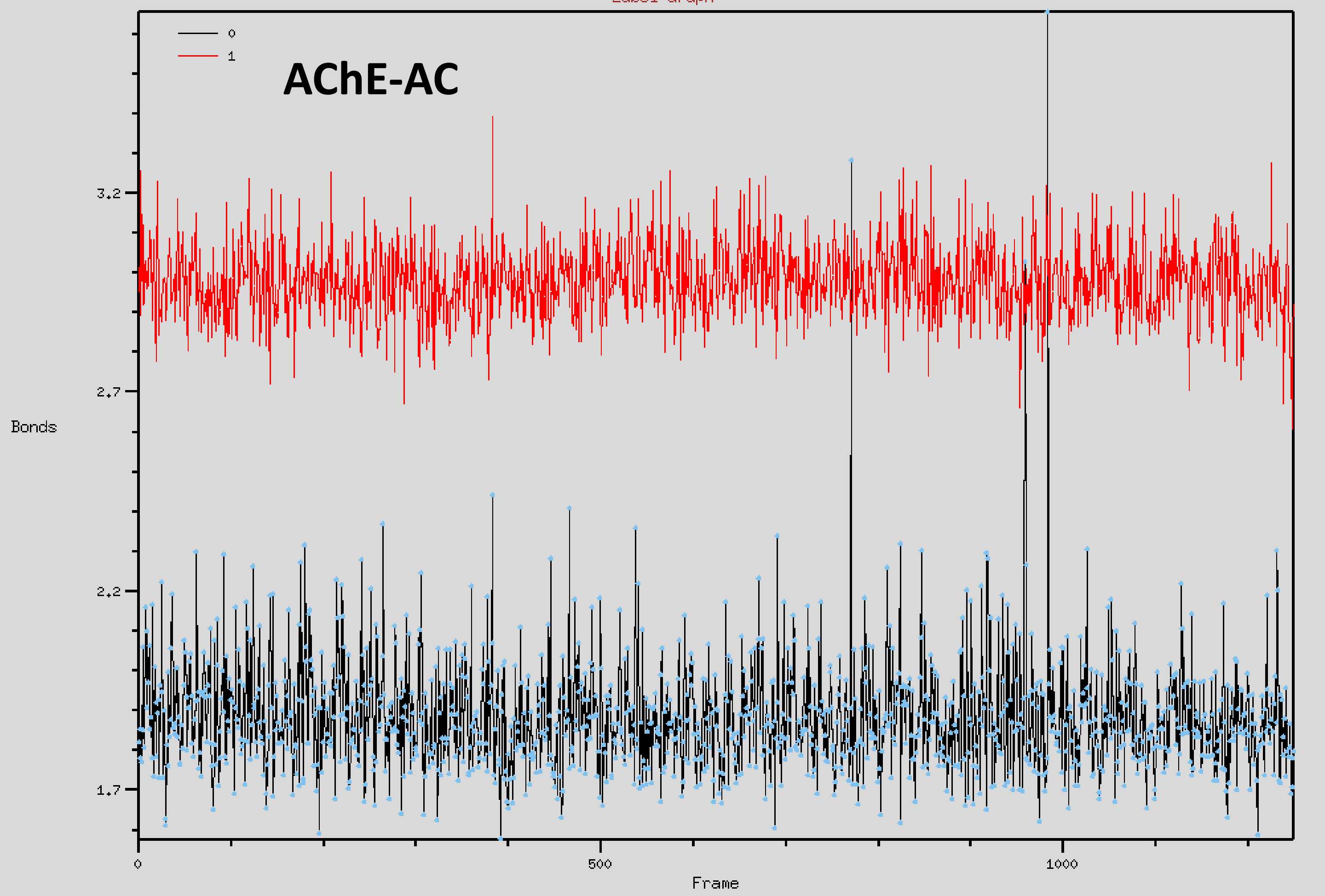

0
1
AChE-AC
3.2
2.7
2.2
1.7
Bonds
0
500
1000
Frame

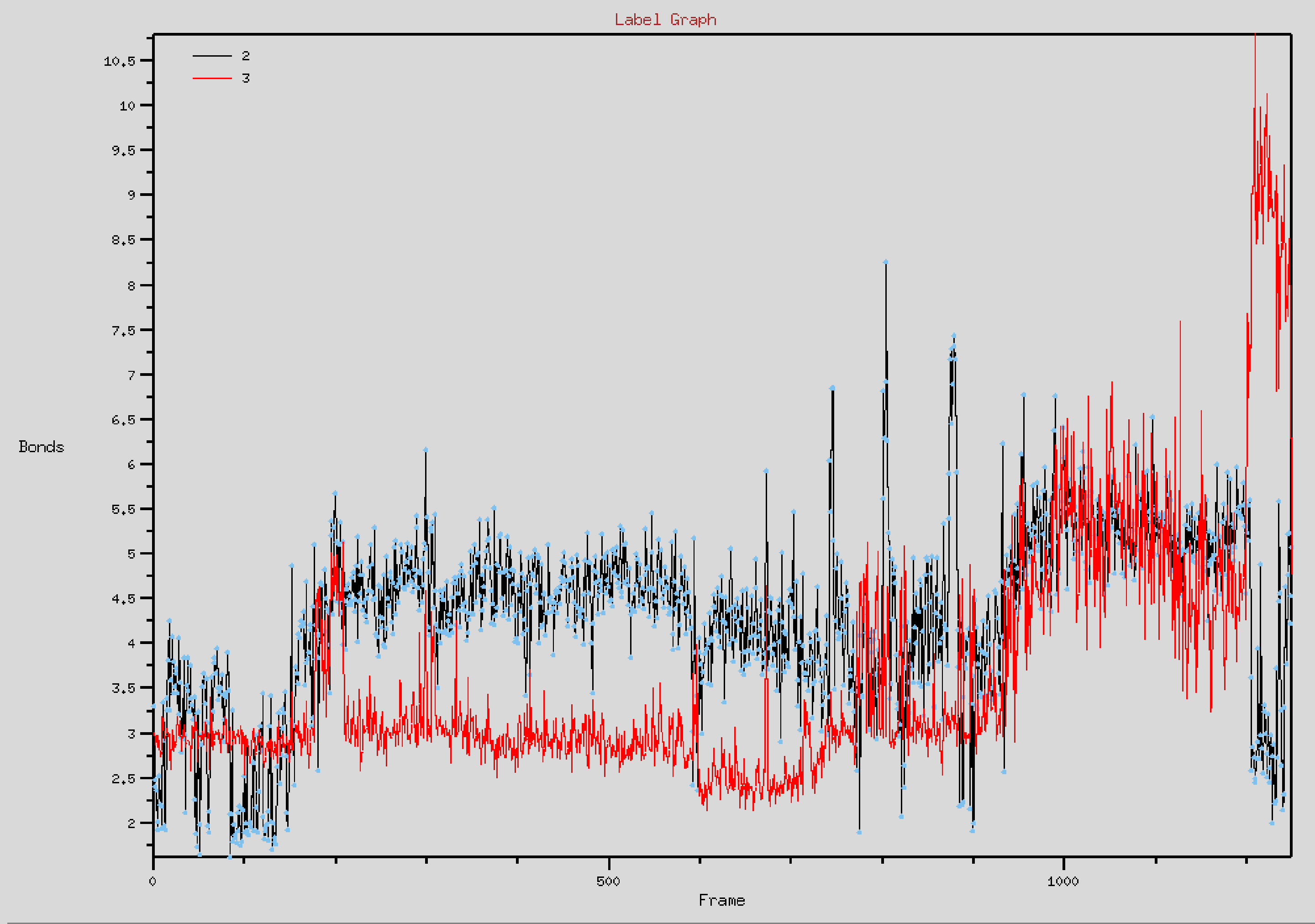

Label Graph
2
3
10.5
10
9.5
9
8.5
8
7.5
7
6.5
6
5.5
5
4.5
4
3.5
3
2.5
2
Bonds
0
500
1000
Frame

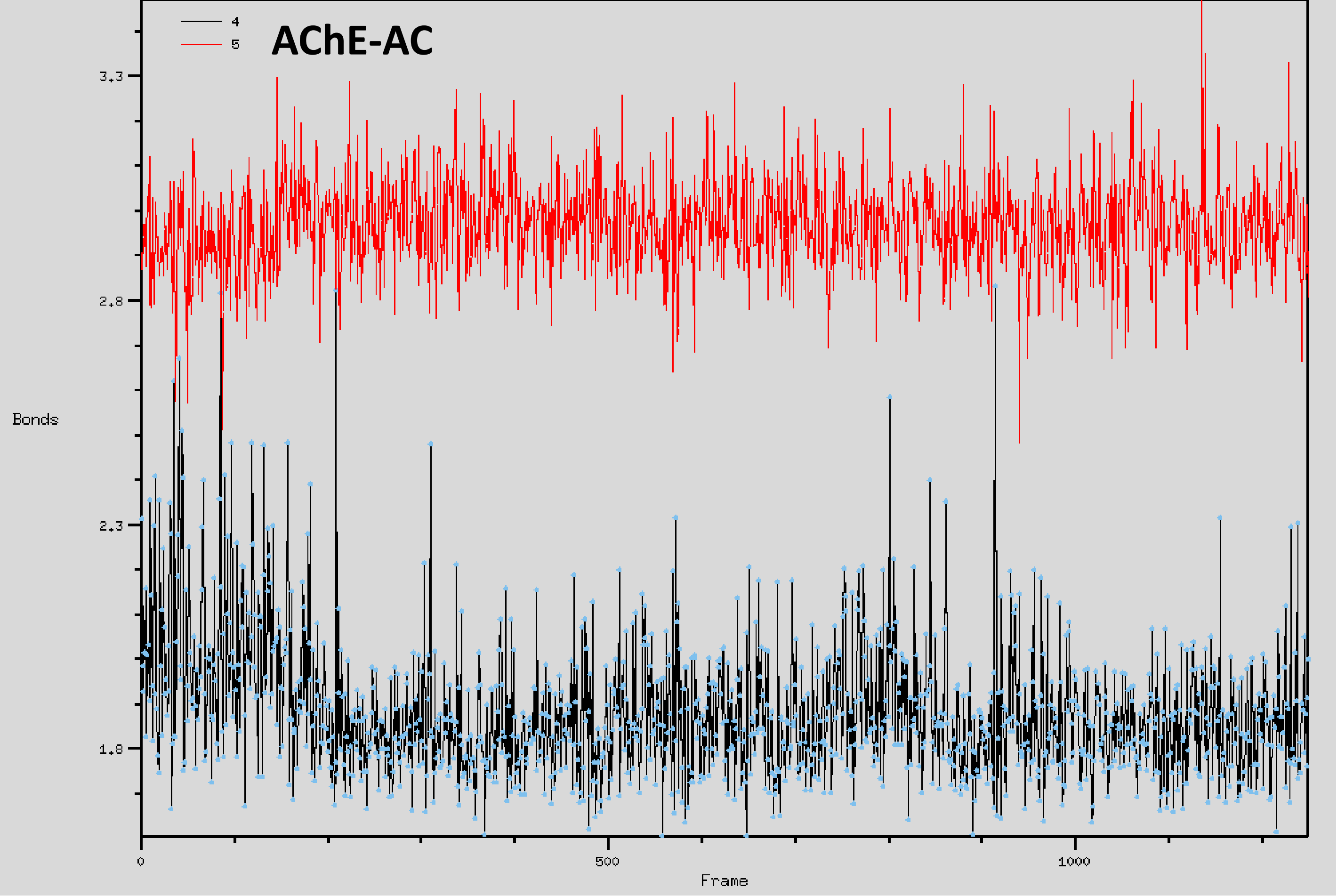

4
5
AChE-AC
3.3
2.8
2.3
1.8
Bonds
0
500
1000
Frame

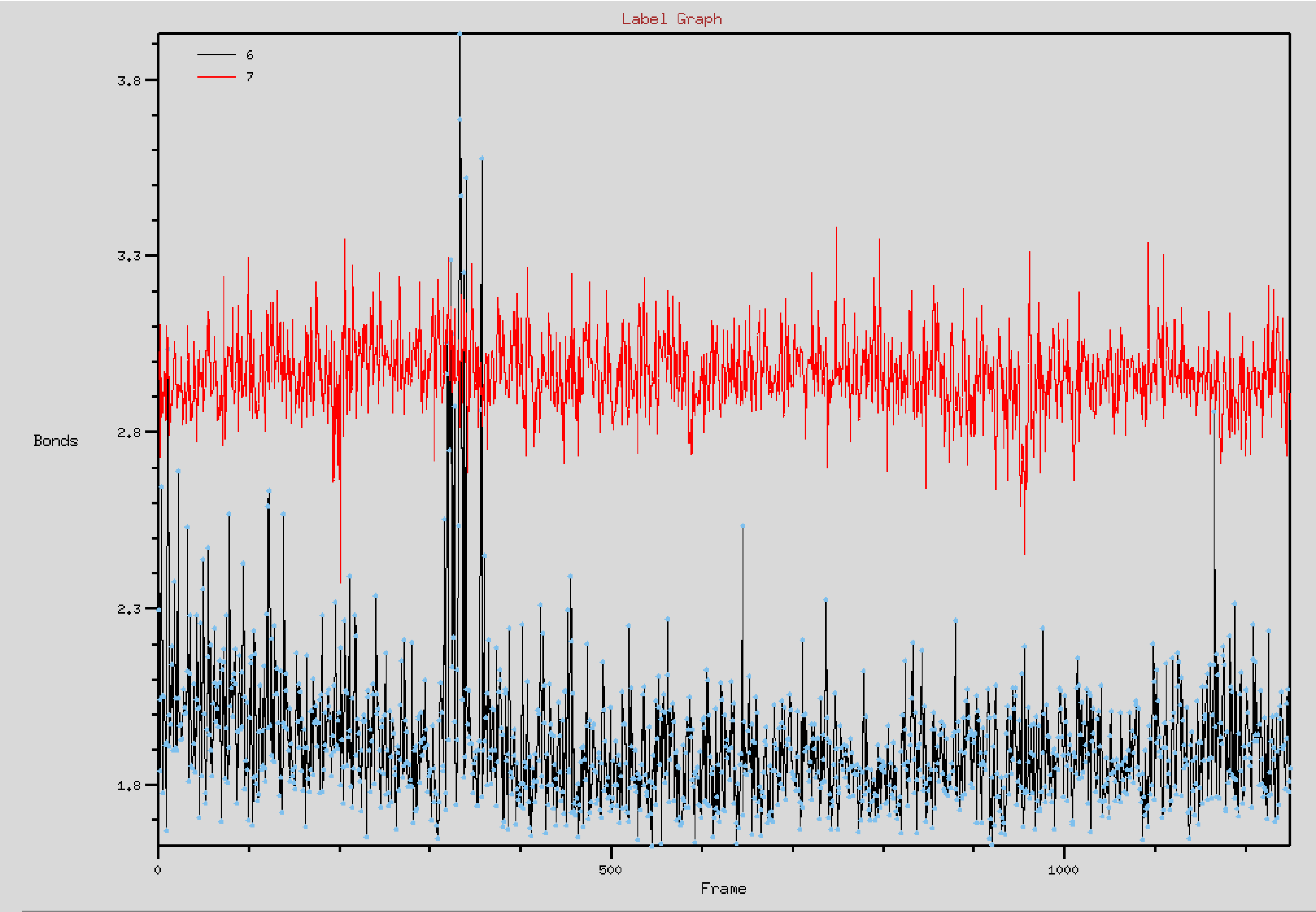

Label Graph
6
7
3.8
3.3
2.8
2.3
1.8
Bonds
0
500
1000
Frame

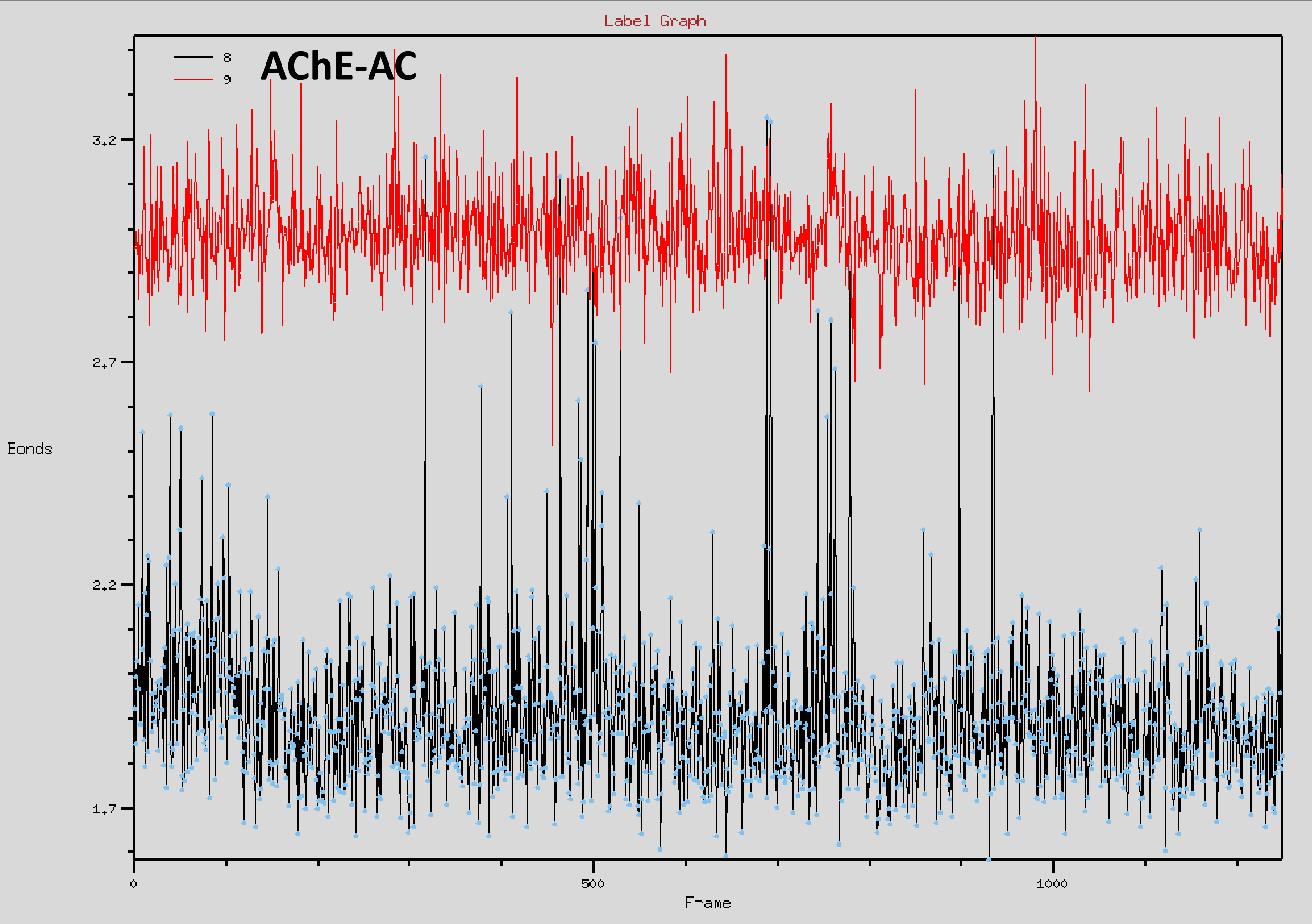
Label Graph
8
9
AChE-AC
Bonds
3.2
2.7
2.2
1.7
0
500
1000
Frame

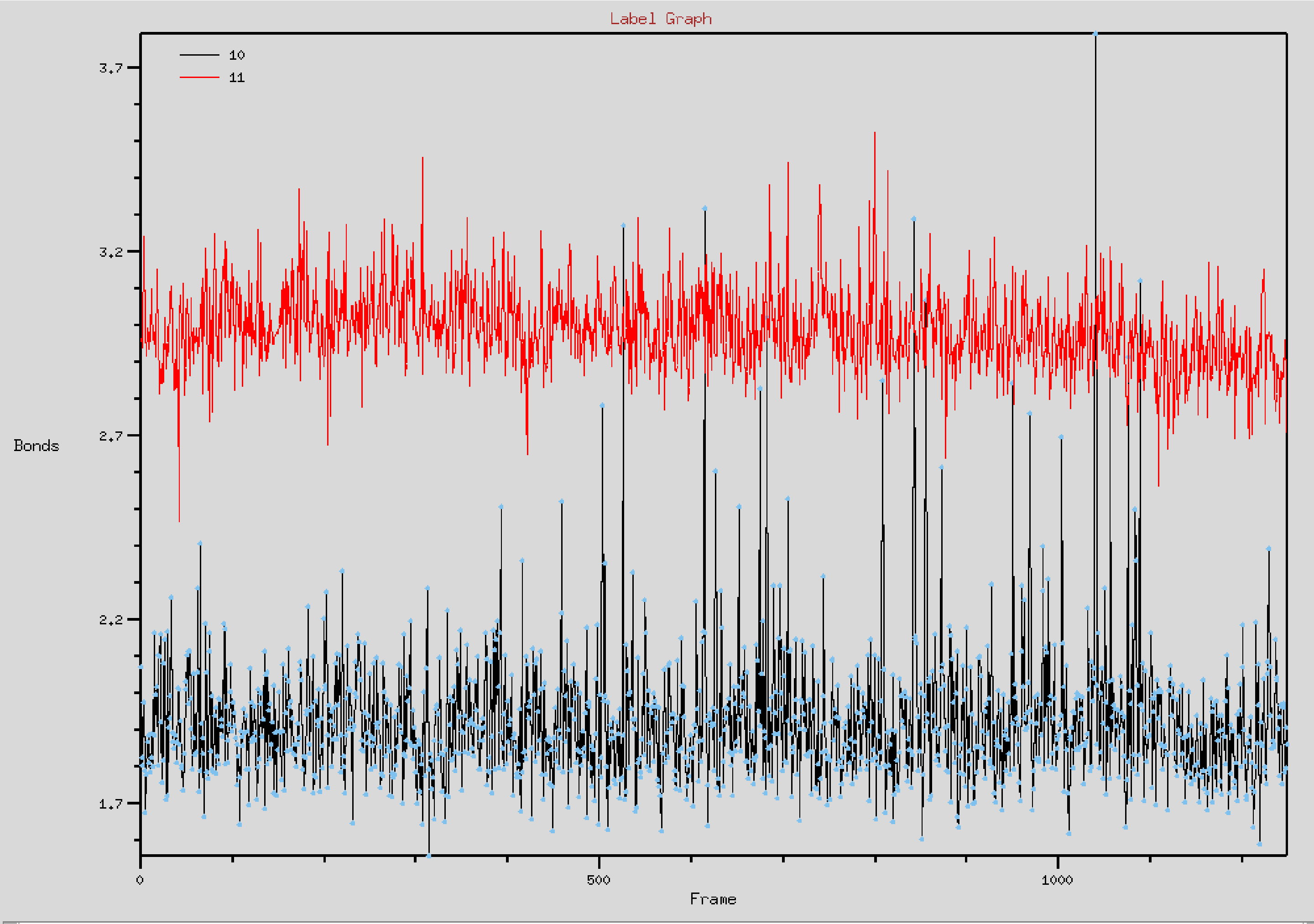
Label Graph
10
11
Bonds
3.7
3.2
2.7
2.2
1.7
0
500
1000
Frame

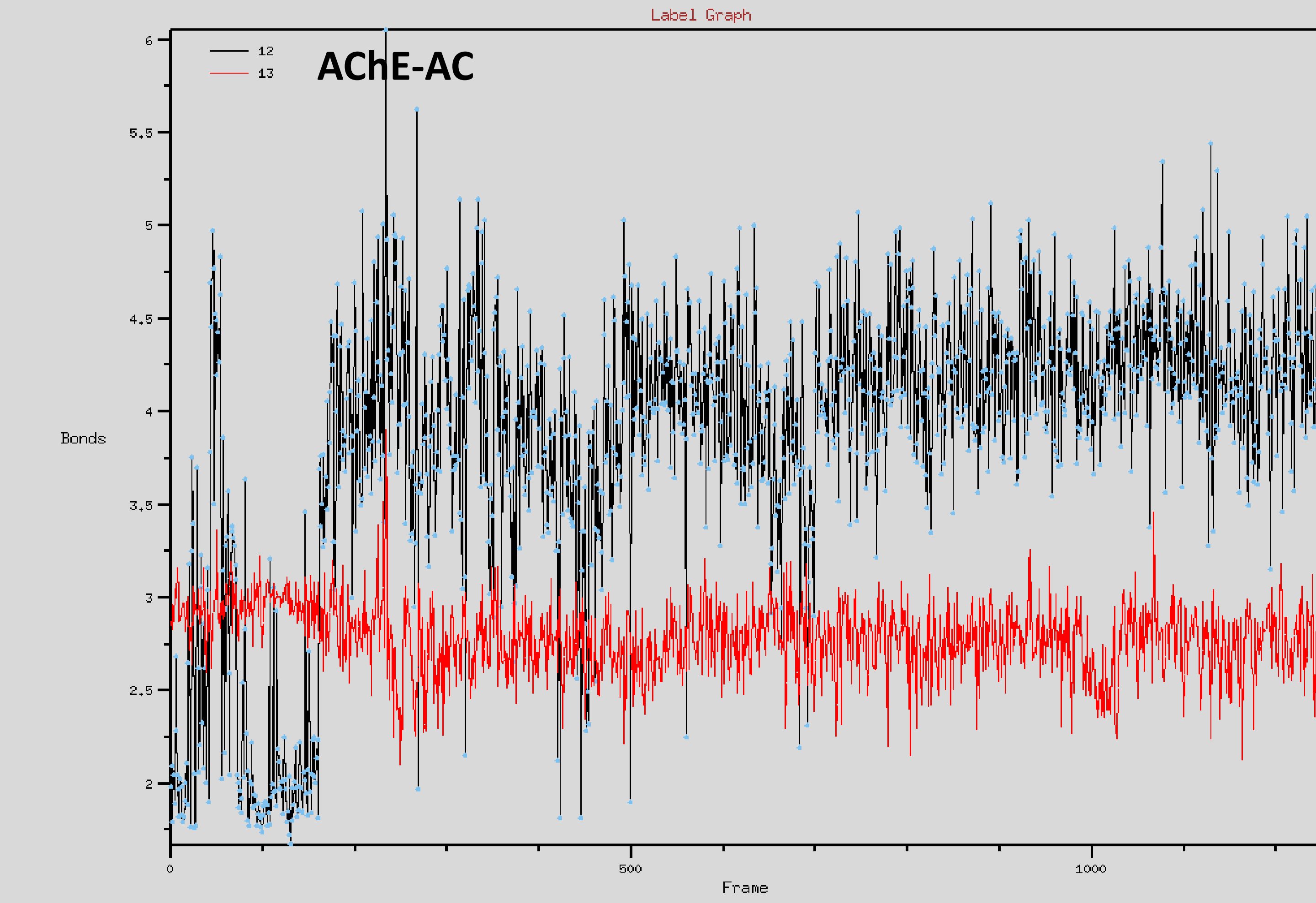
Label Graph
12
13
AChE-AC
Bonds
Frame
6
5.5
5
4.5
4
3.5
3
2.5
2
0
500
1000

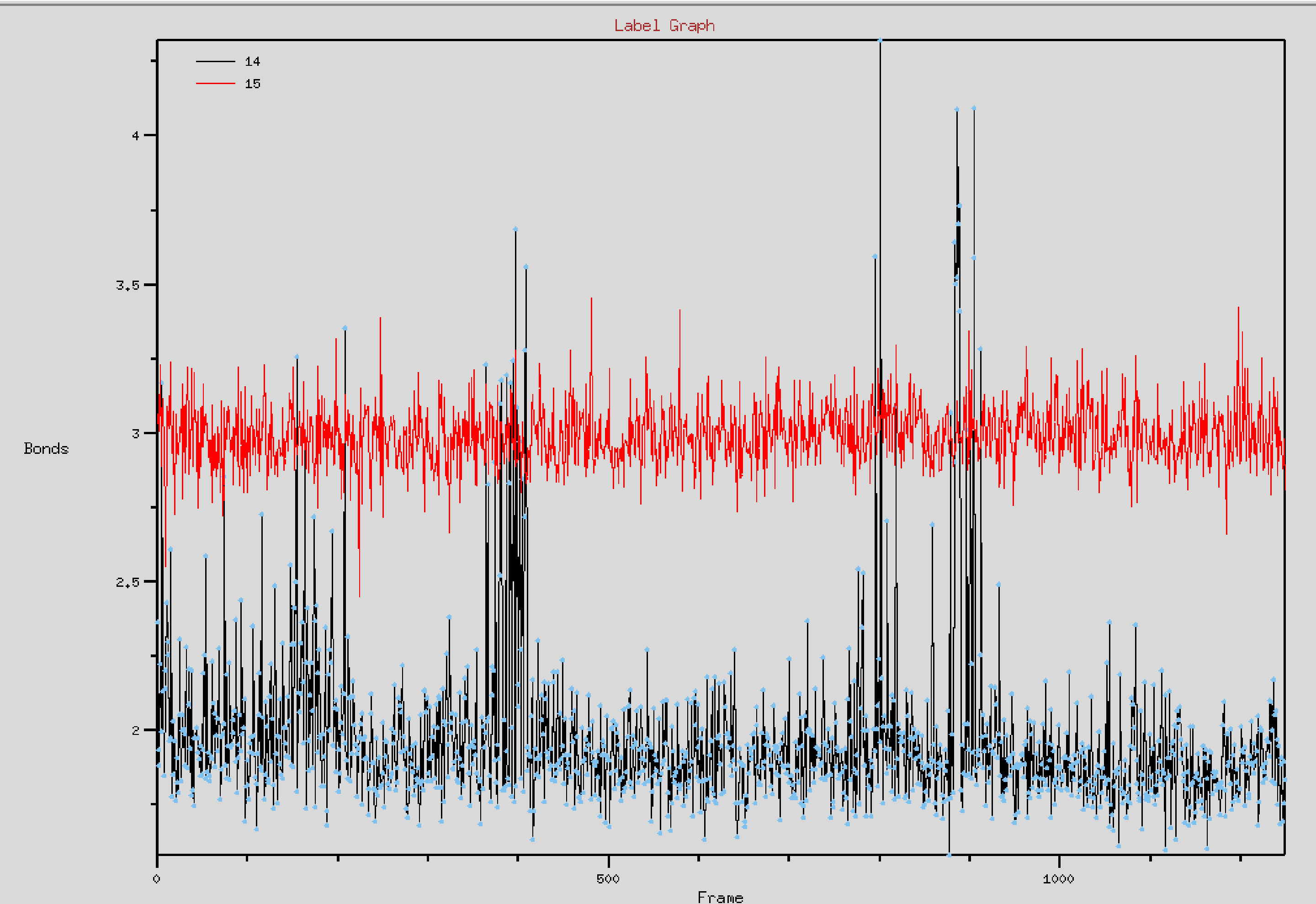
Label Graph
14
15
Bonds
Frame
4
3.5
3
2.5
2
0
500
1000

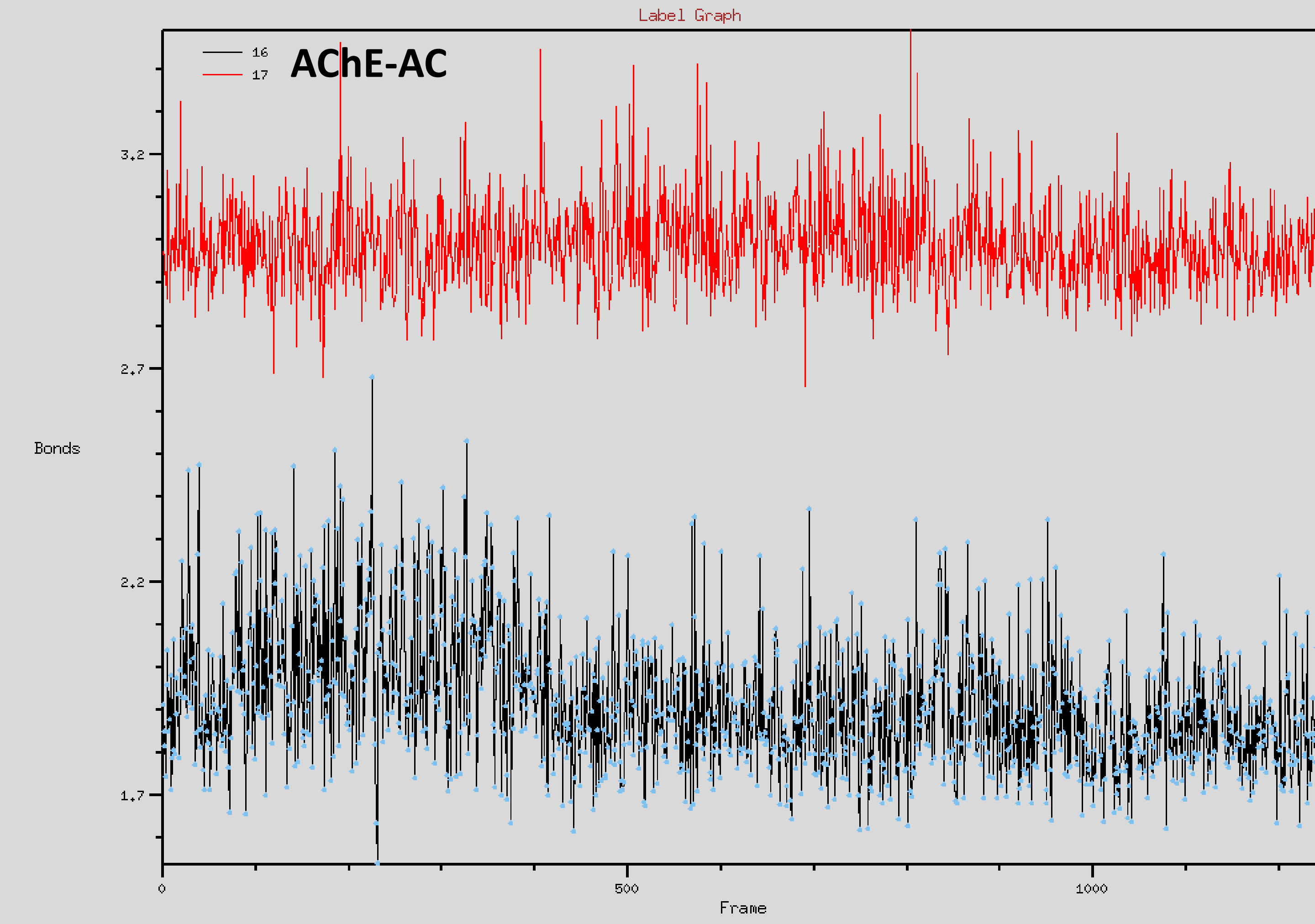
Label Graph
16
17
AChE-AC
3.2
2.7
2.2
1.7
Bonds
0
500
1000
Frame

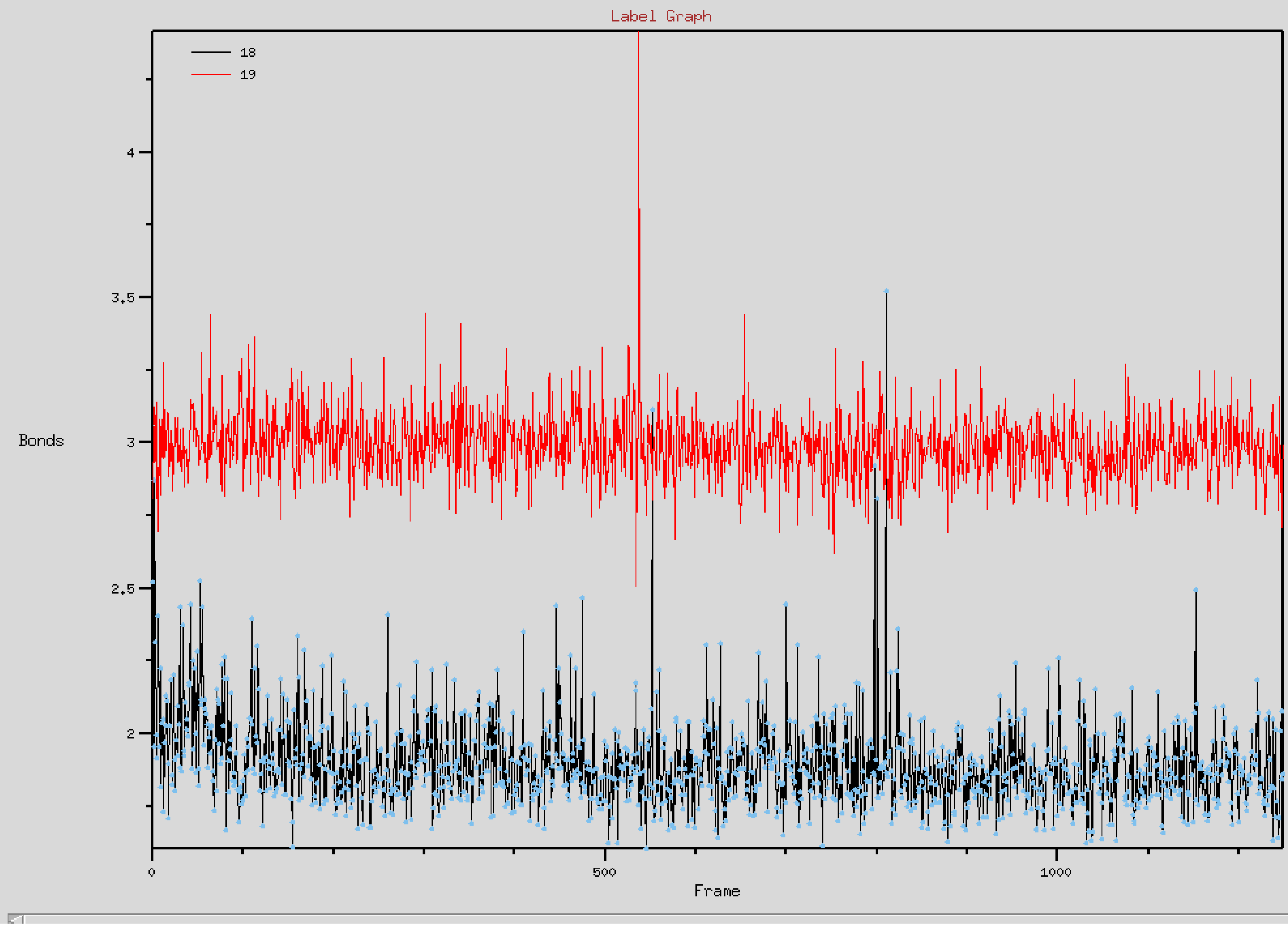
Label Graph
18
19
4
3.5
3
2.5
2
Bonds
0
500
1000
Frame

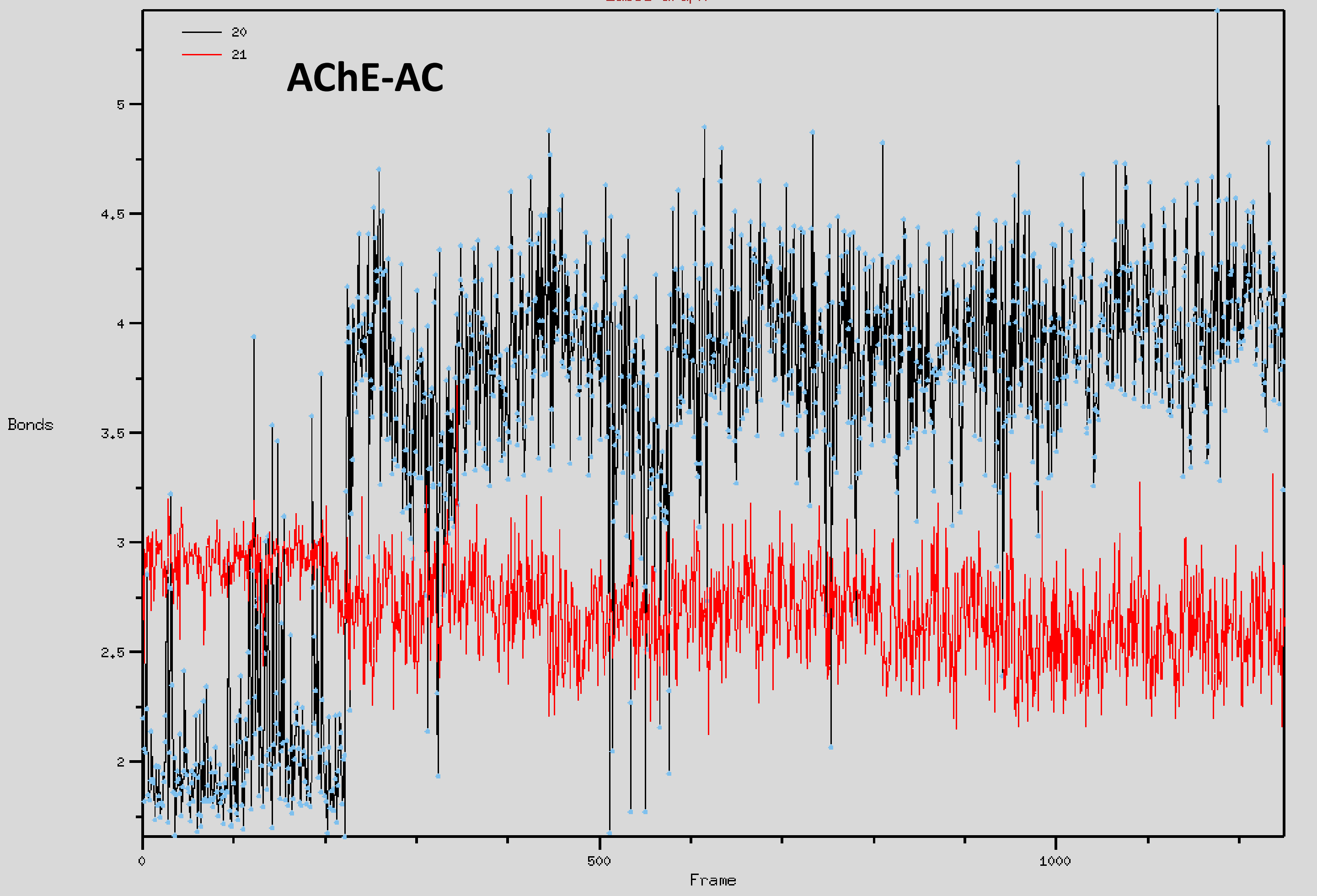
20
21
AChE-AC
Bonds
Frame
5
4.5
4
3.5
3
2.5
2
0
500
1000

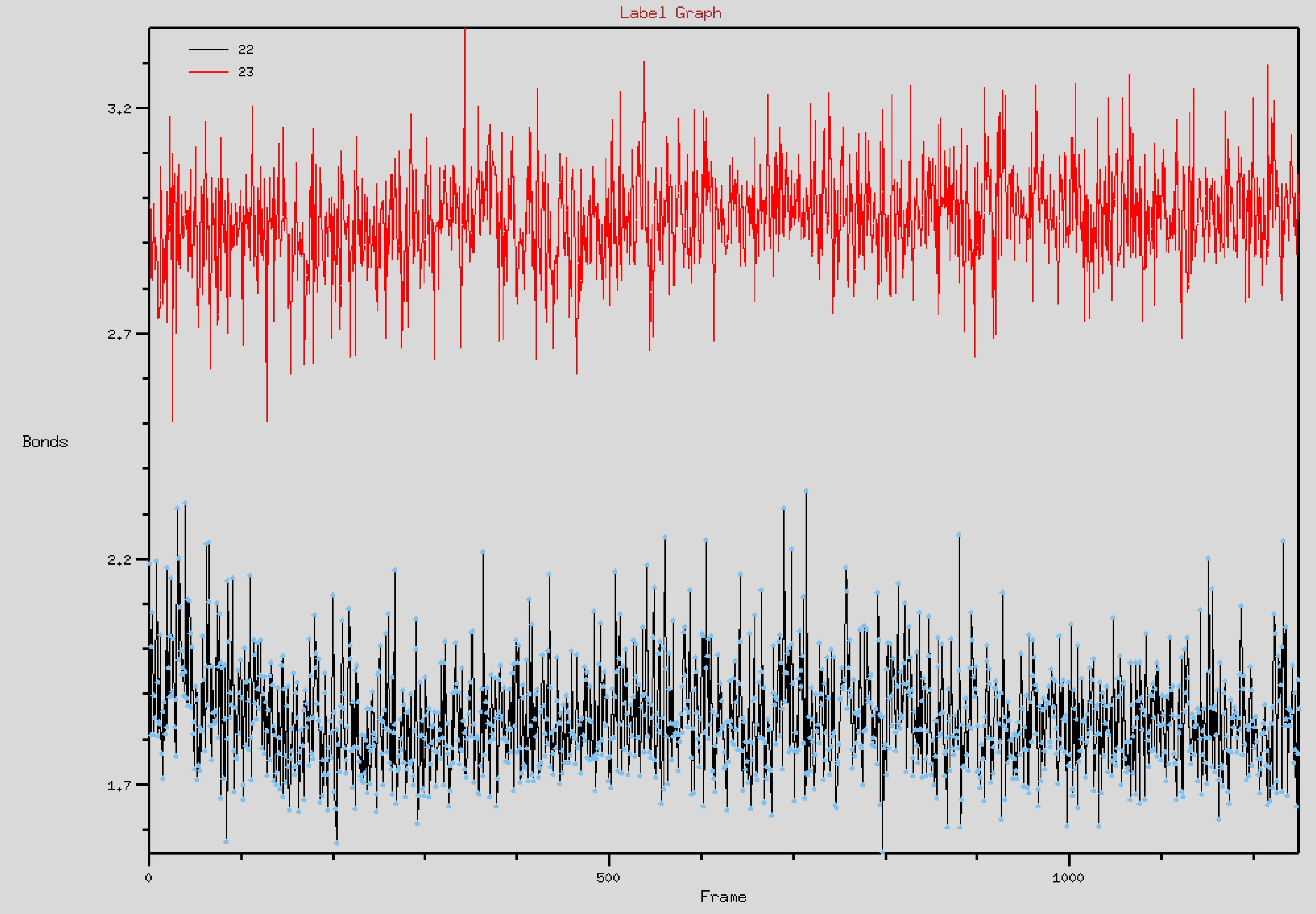
Label Graph
22
23
Bonds
Frame
3.2
2.7
2.2
1.7
0
500
1000

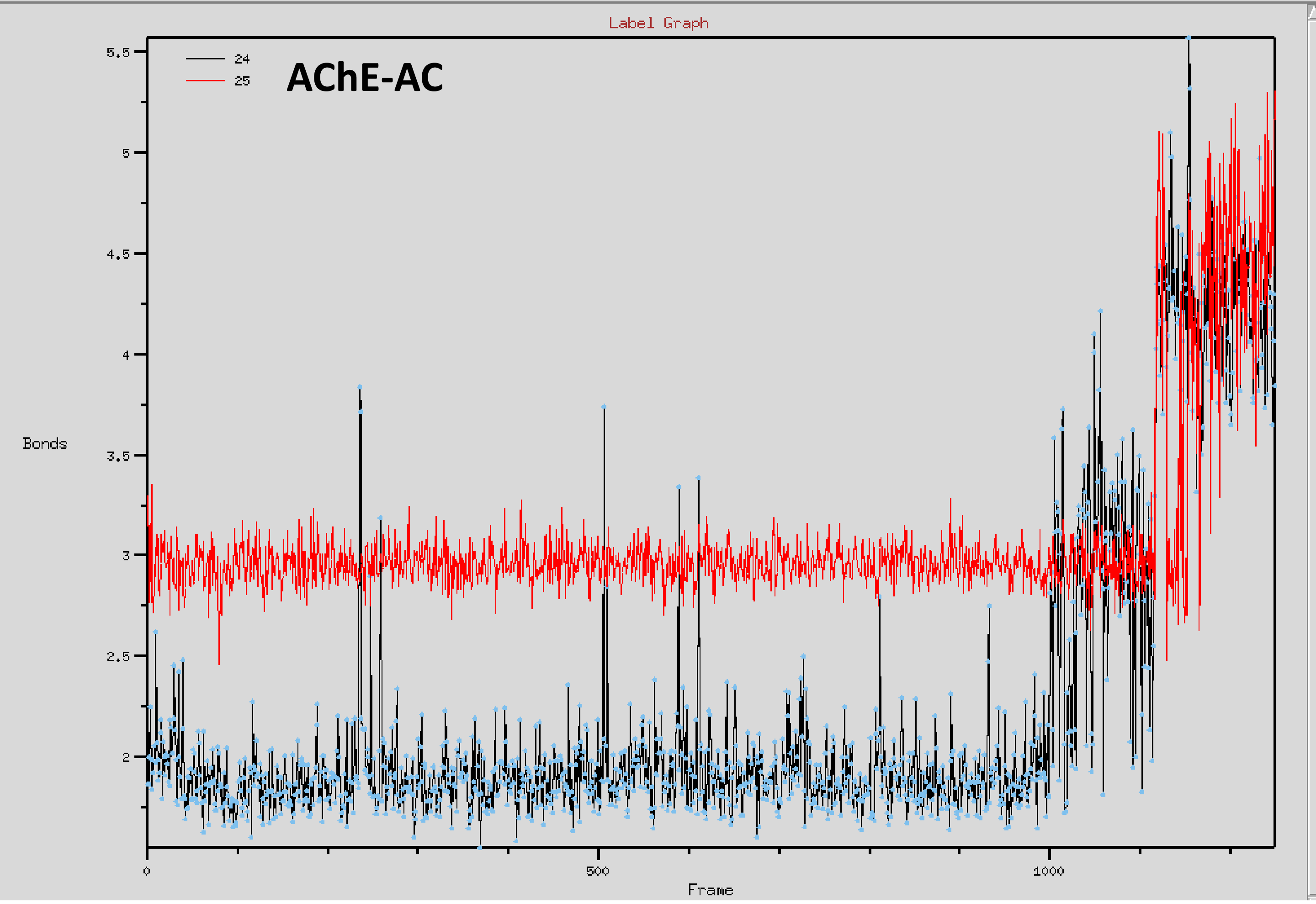
Label Graph
24
25
AChE-AC
Bonds
Frame
5,5
5
4,5
4
3,5
3
2,5
2
0
500
1000

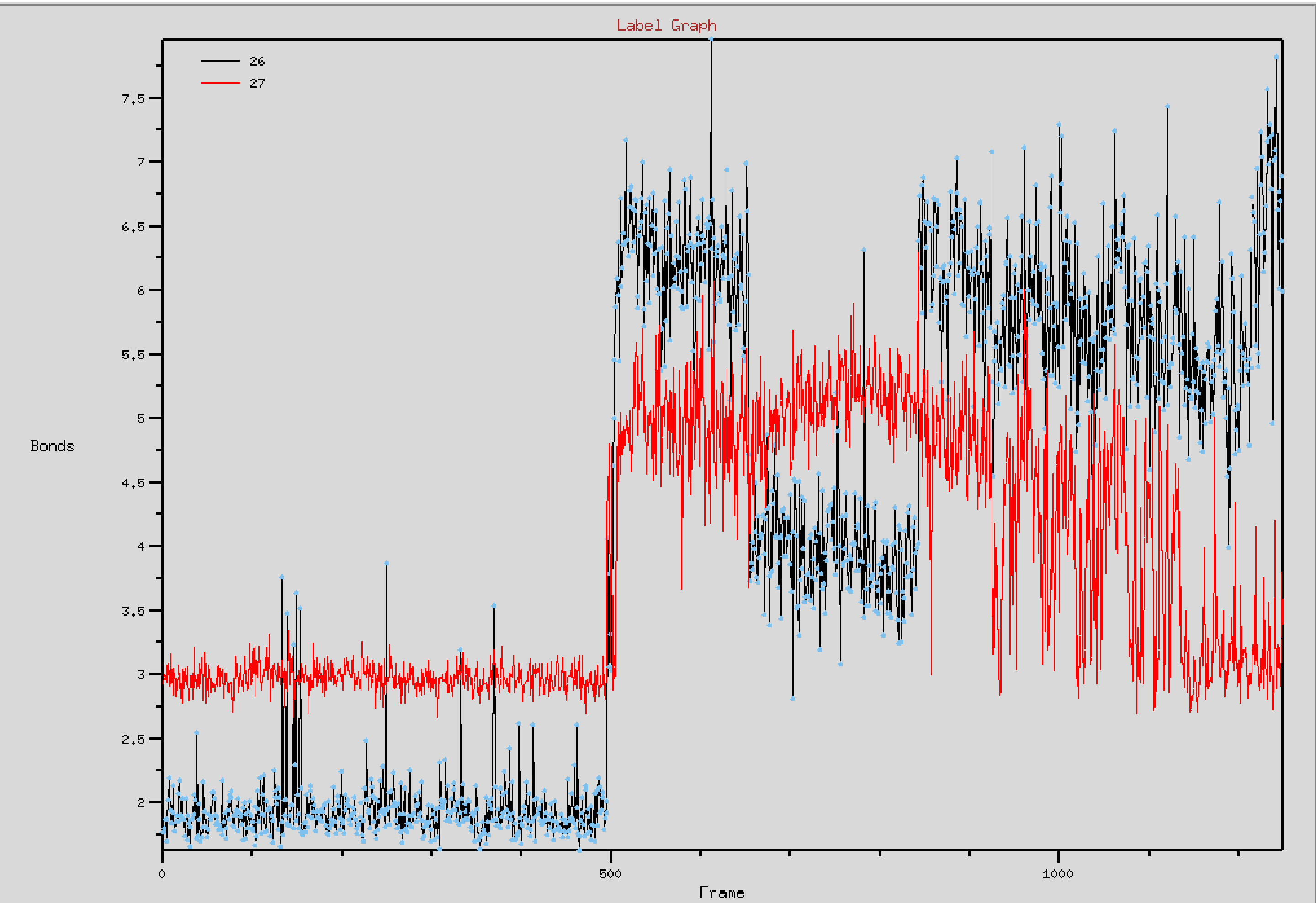
Label Graph
26
27
Bonds
Frame
7,5
7
6,5
6
5,5
5
4,5
4
3,5
3
2,5
2
0
500
1000

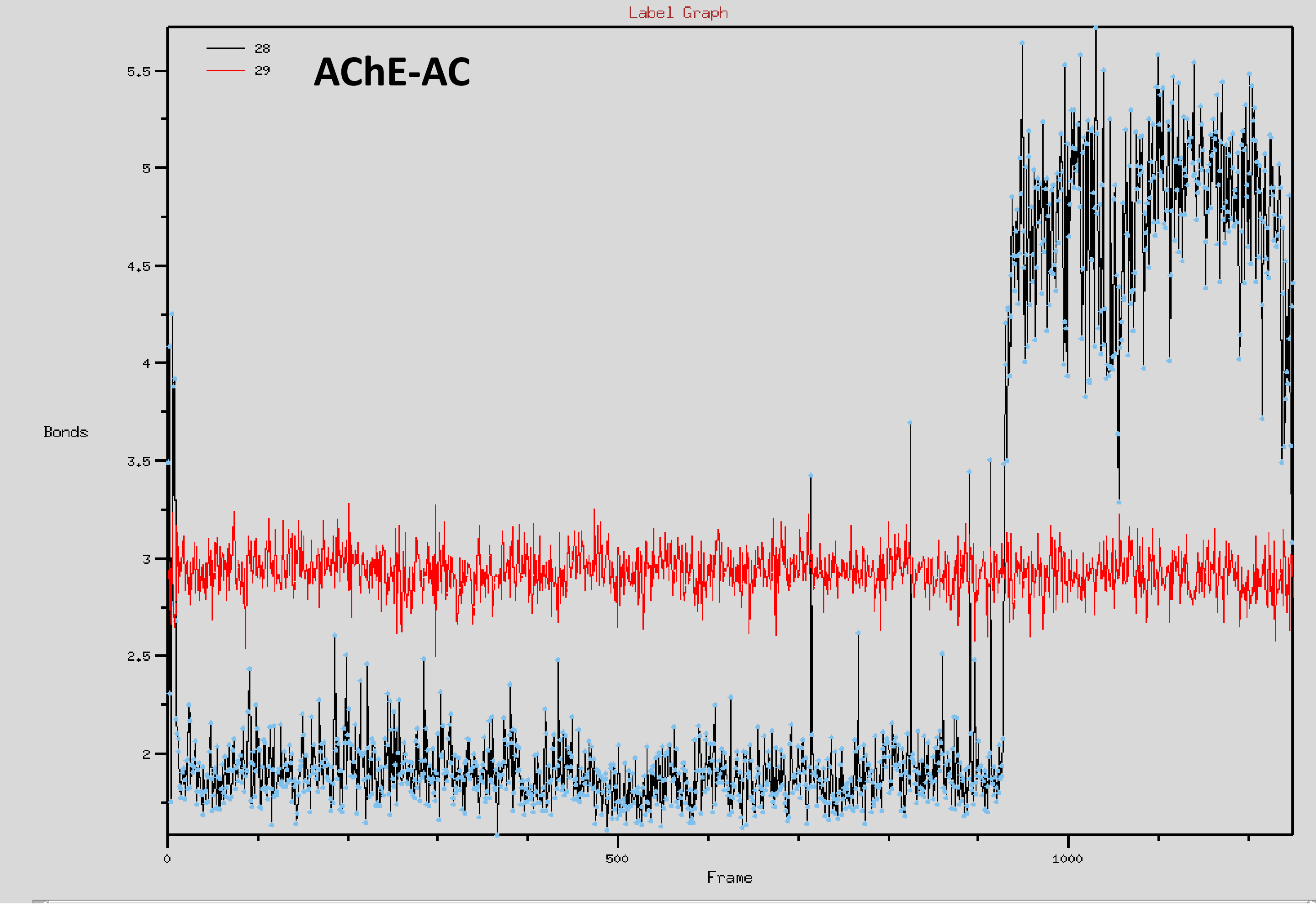
Label Graph
28
29
AChE-AC
Bonds
Frame
5.5
5
4.5
4
3.5
3
2.5
2
0
500
1000

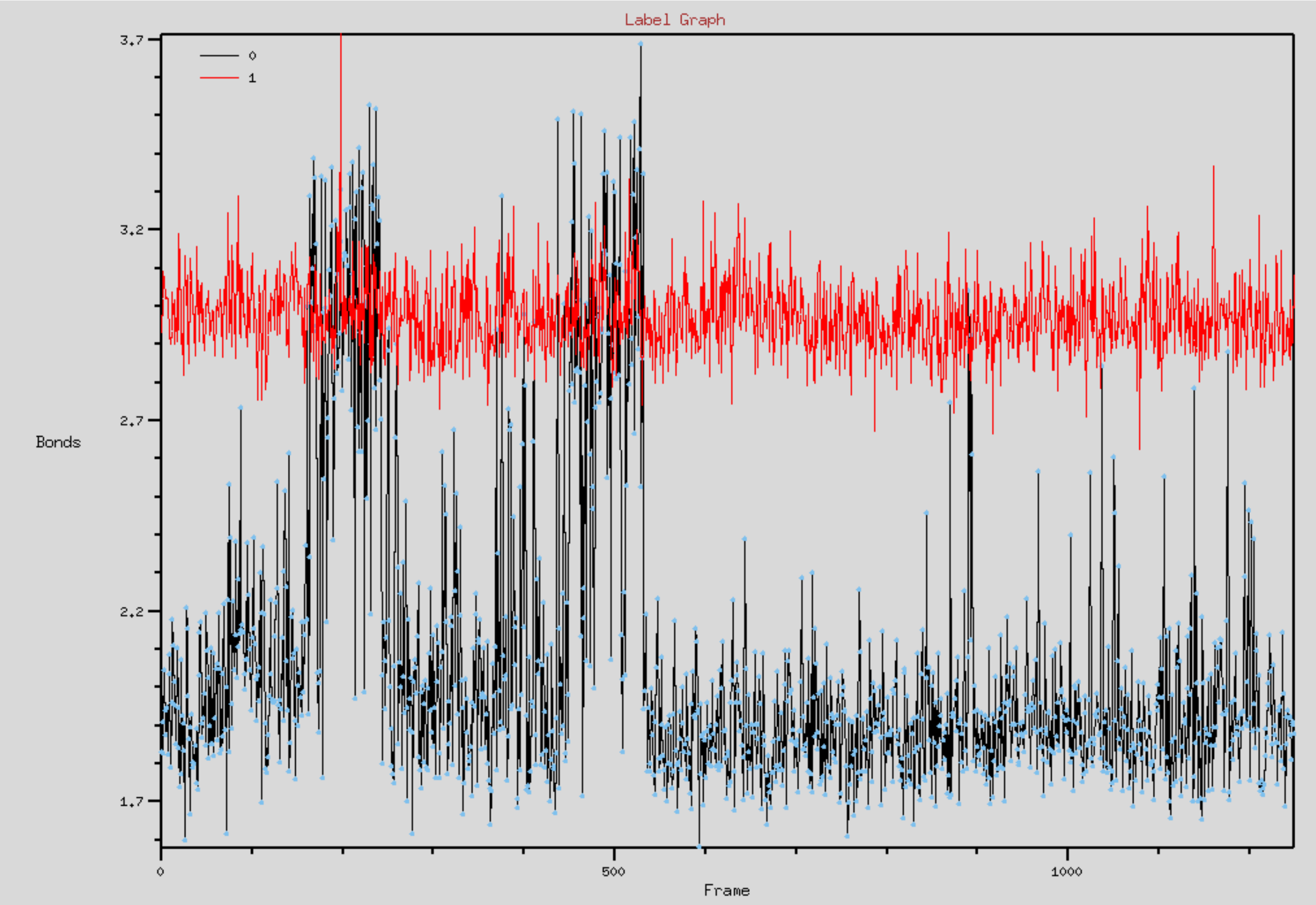
Label Graph
0
1
Bonds
Frame
3.7
3.2
2.7
2.2
1.7
0
500
1000

AChE-1

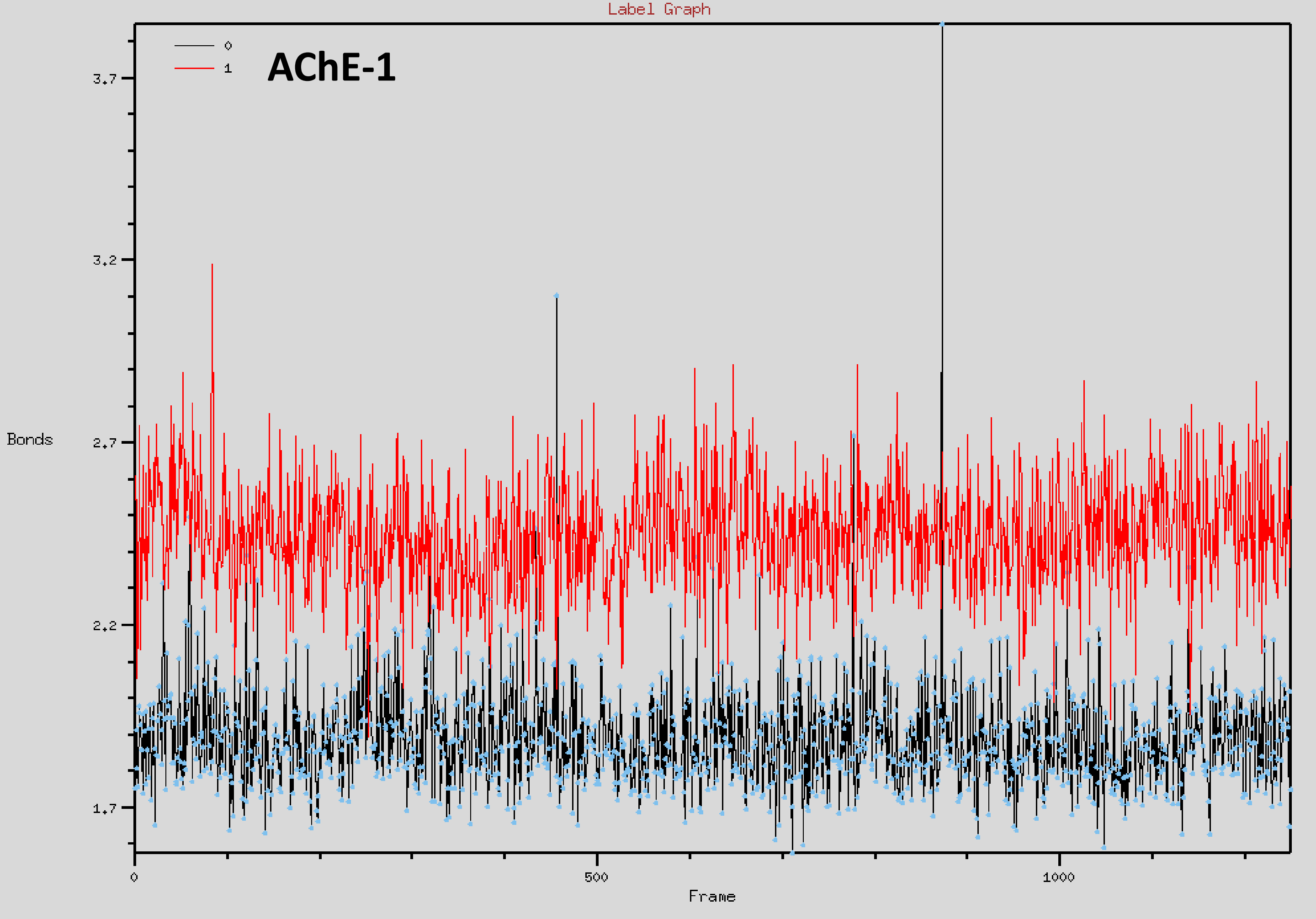
Label Graph
0
1
AChE-1
Bonds
3.7
3.2
2.7
2.2
1.7
0
500
1000
Frame

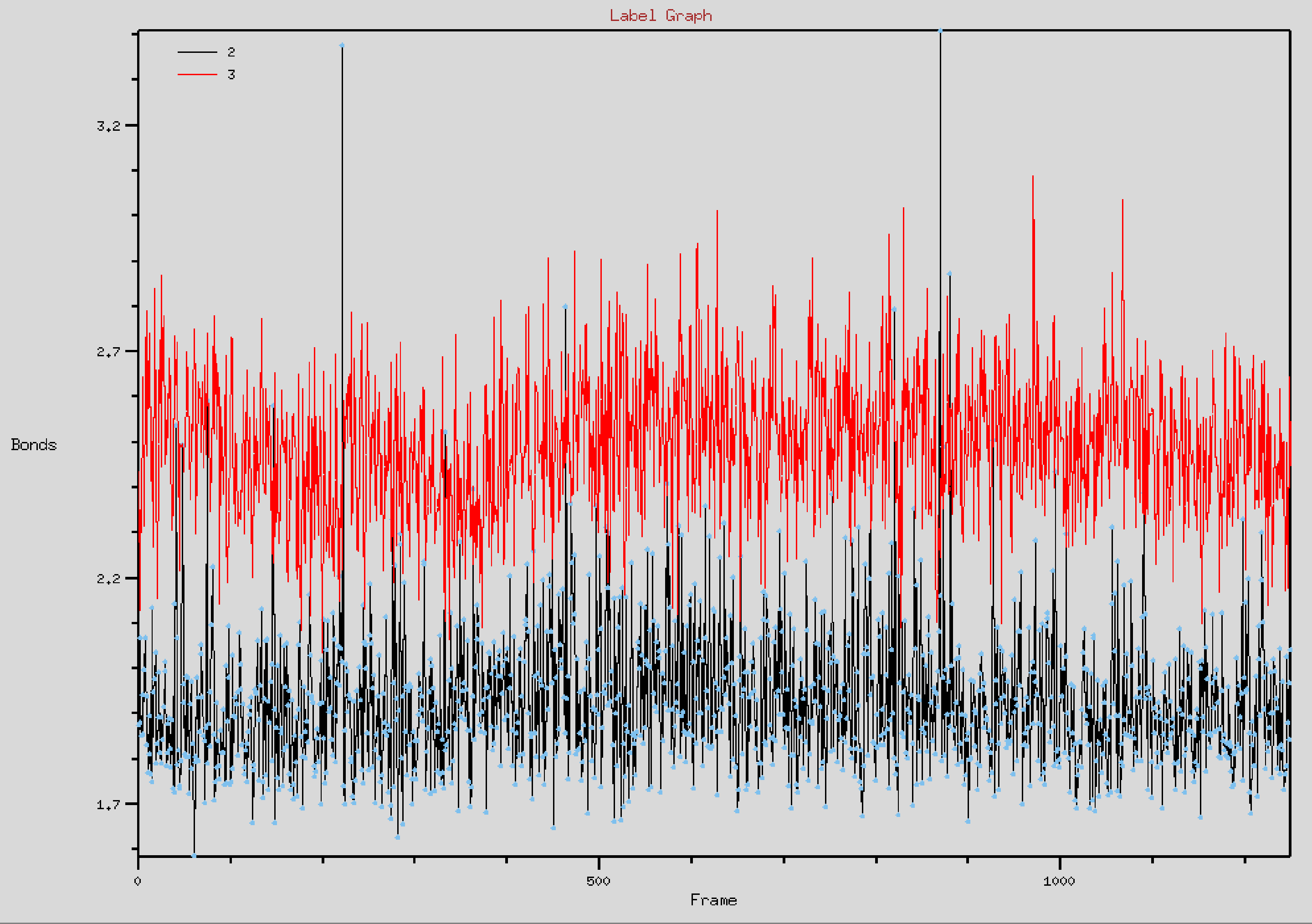
Label Graph
2
3
Bonds
3.2
2.7
2.2
1.7
0
500
1000
Frame

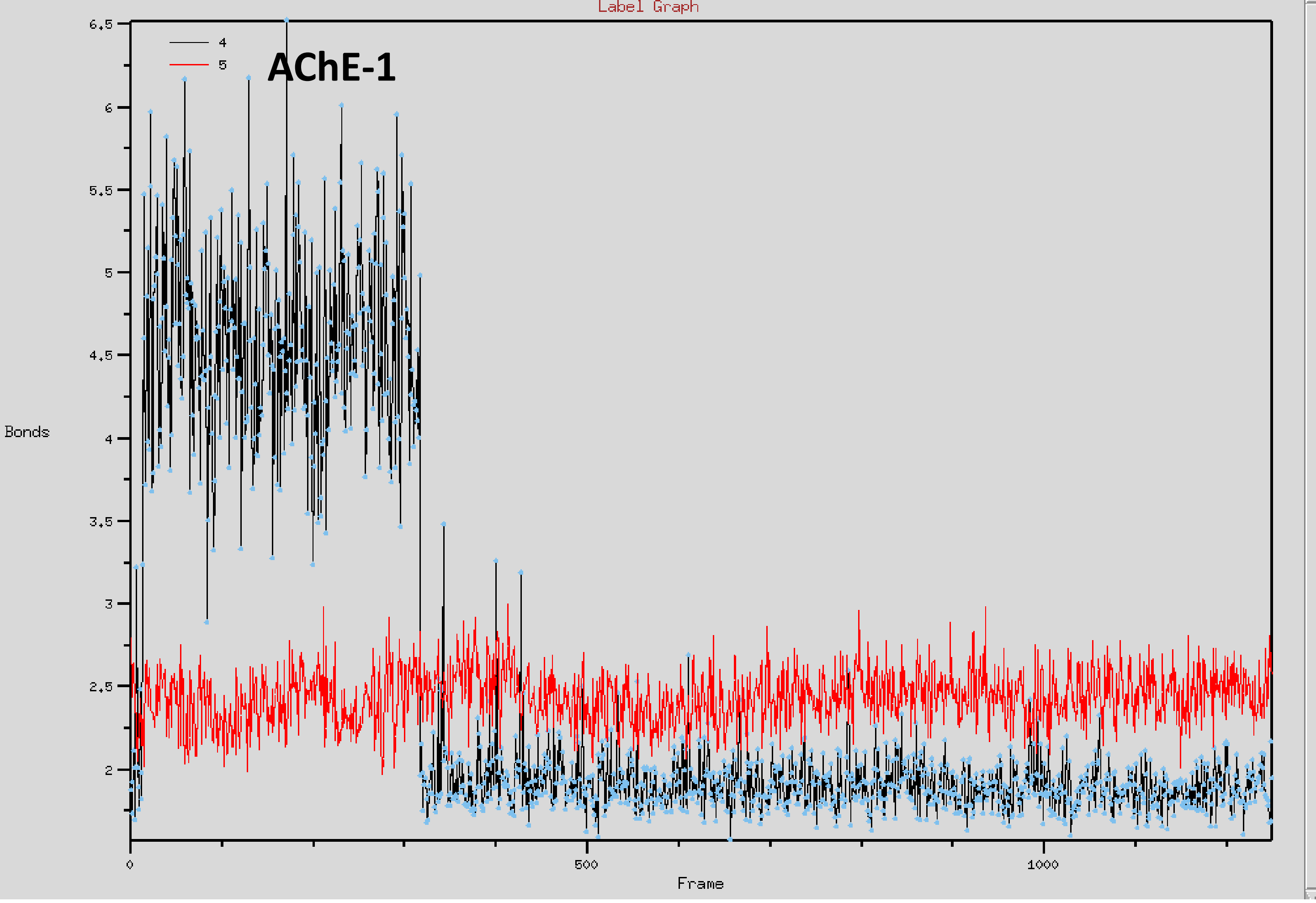
Label Graph
4
5
AChE-1
Bonds
Frame
6.5
6
5.5
5
4.5
4
3.5
3
2.5
2
0
500
1000

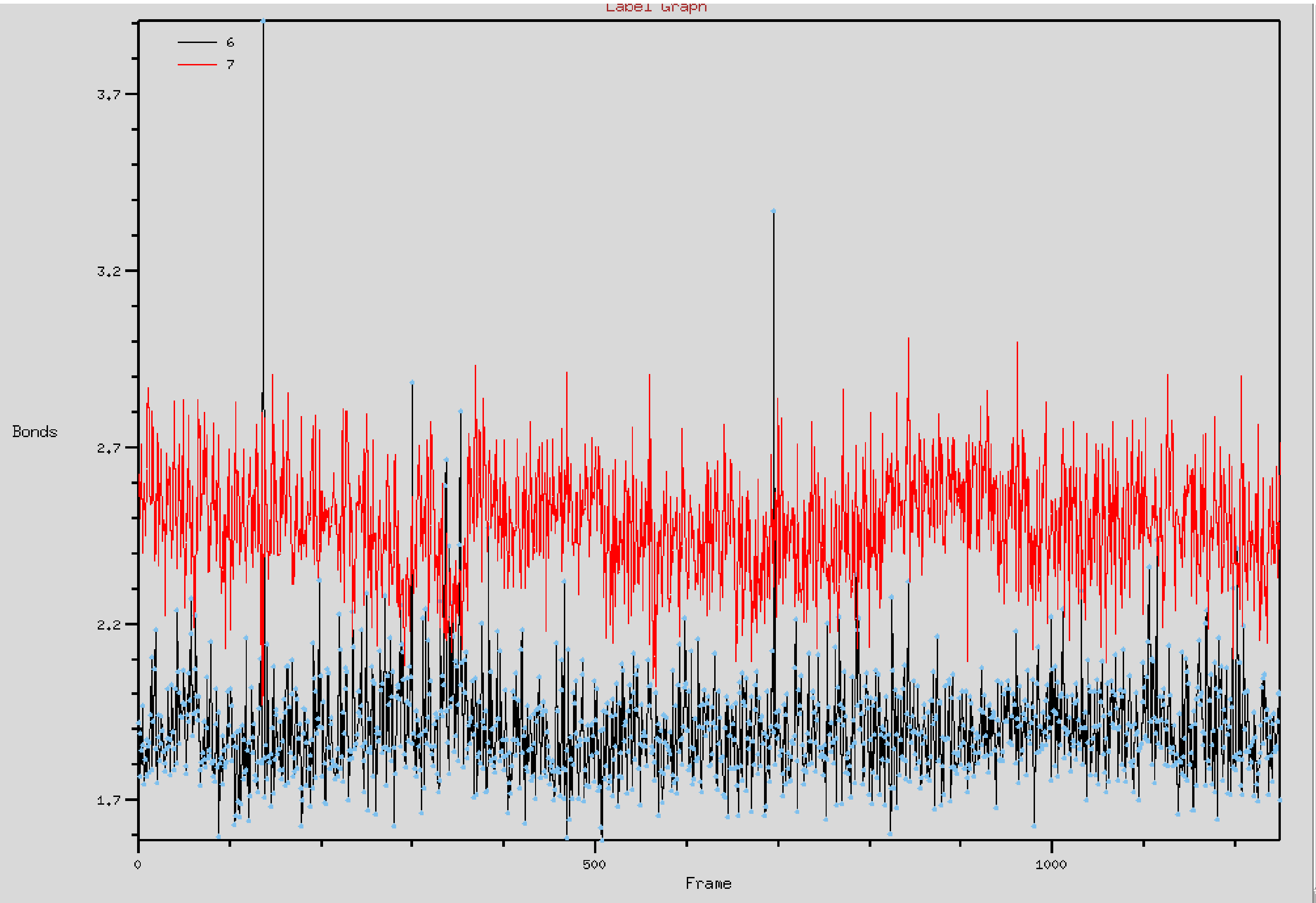
Label Graph
6
7
Bonds
Frame
3.7
3.2
2.7
2.2
1.7
0
500
1000

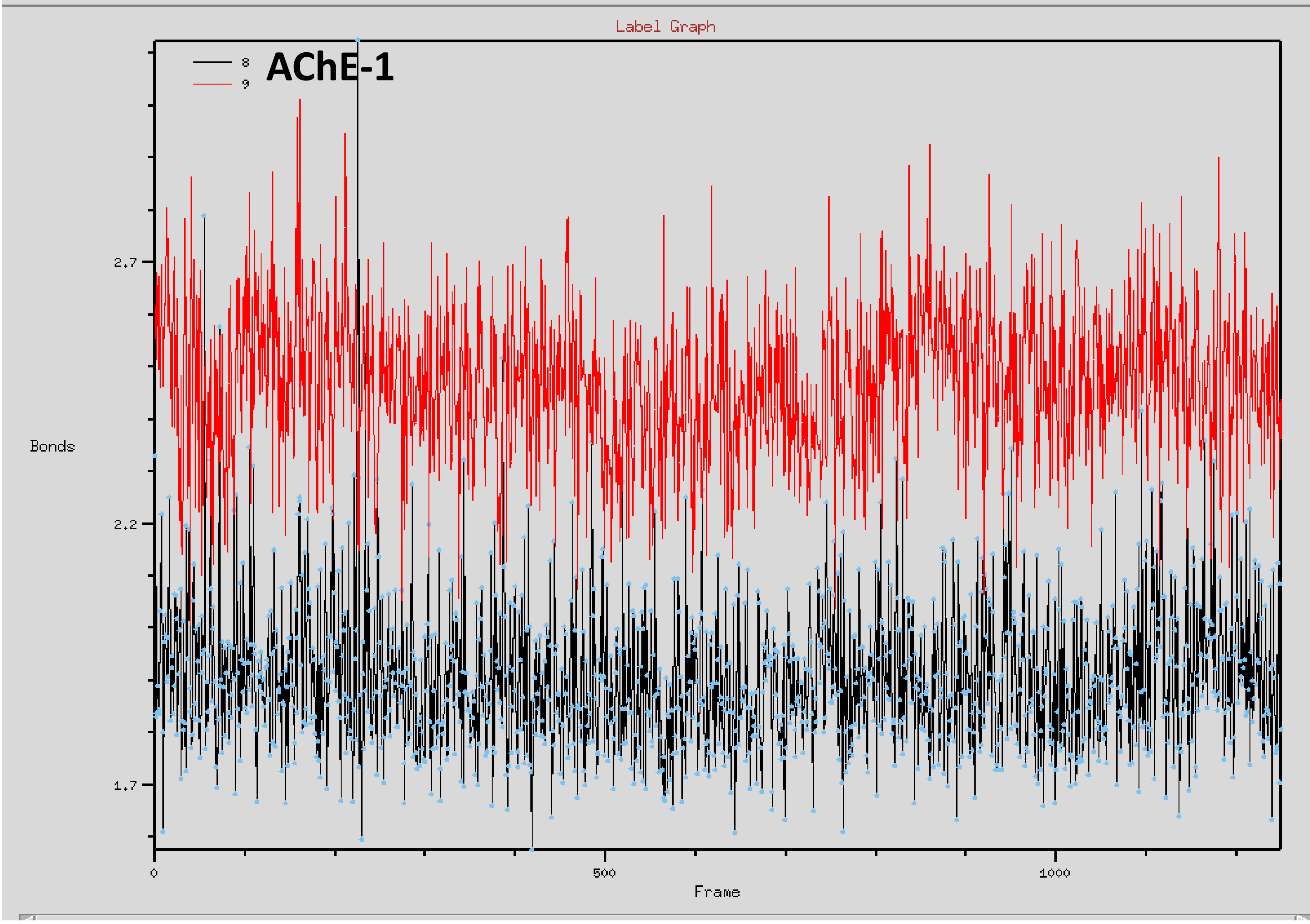

Label Graph
AChE-1
8
9
2.7
2.2
1.7
Bonds
0
500
1000
Frame

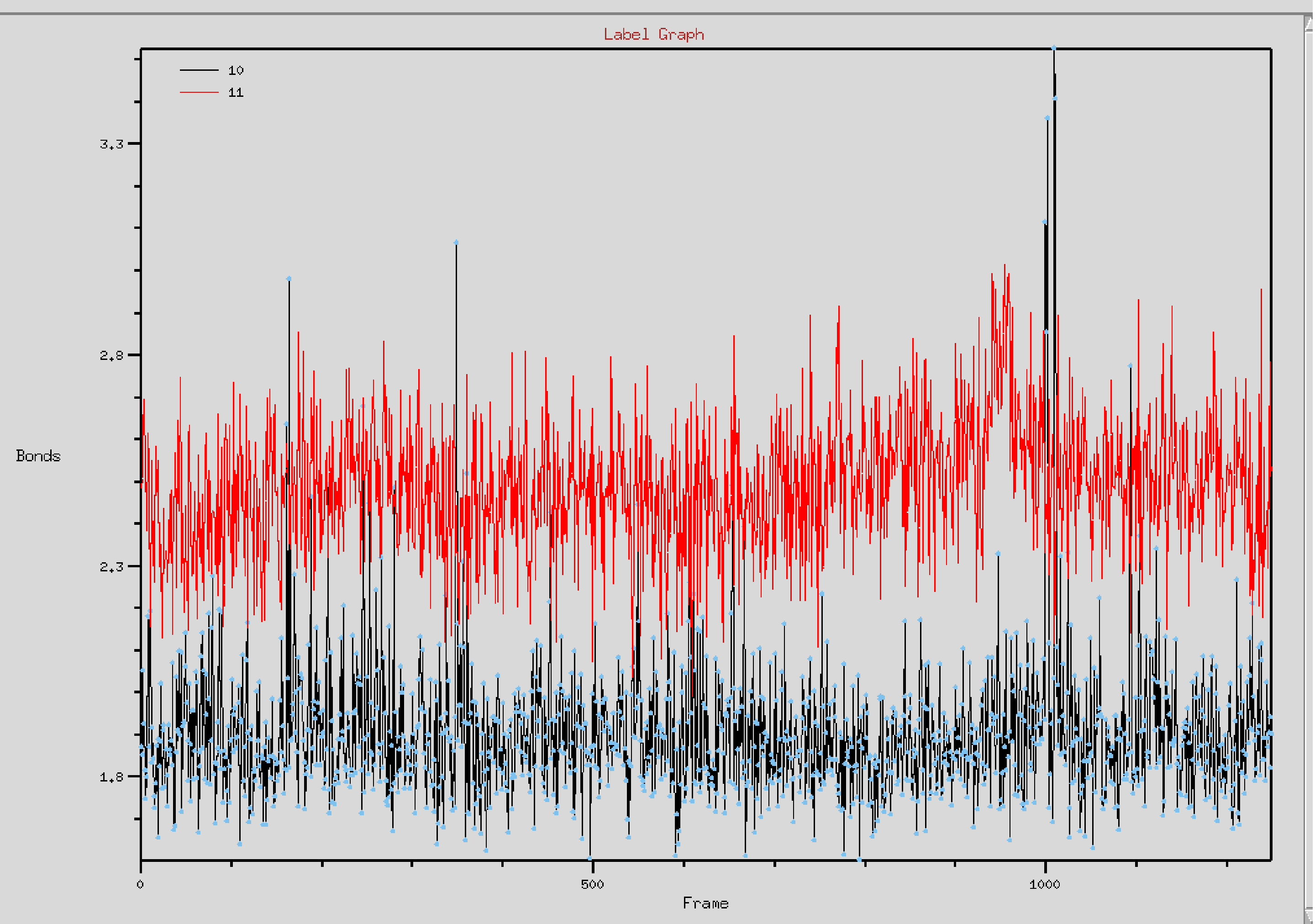

Label Graph
10
11
3.3
2.8
2.3
1.8
Bonds
0
500
1000
Frame

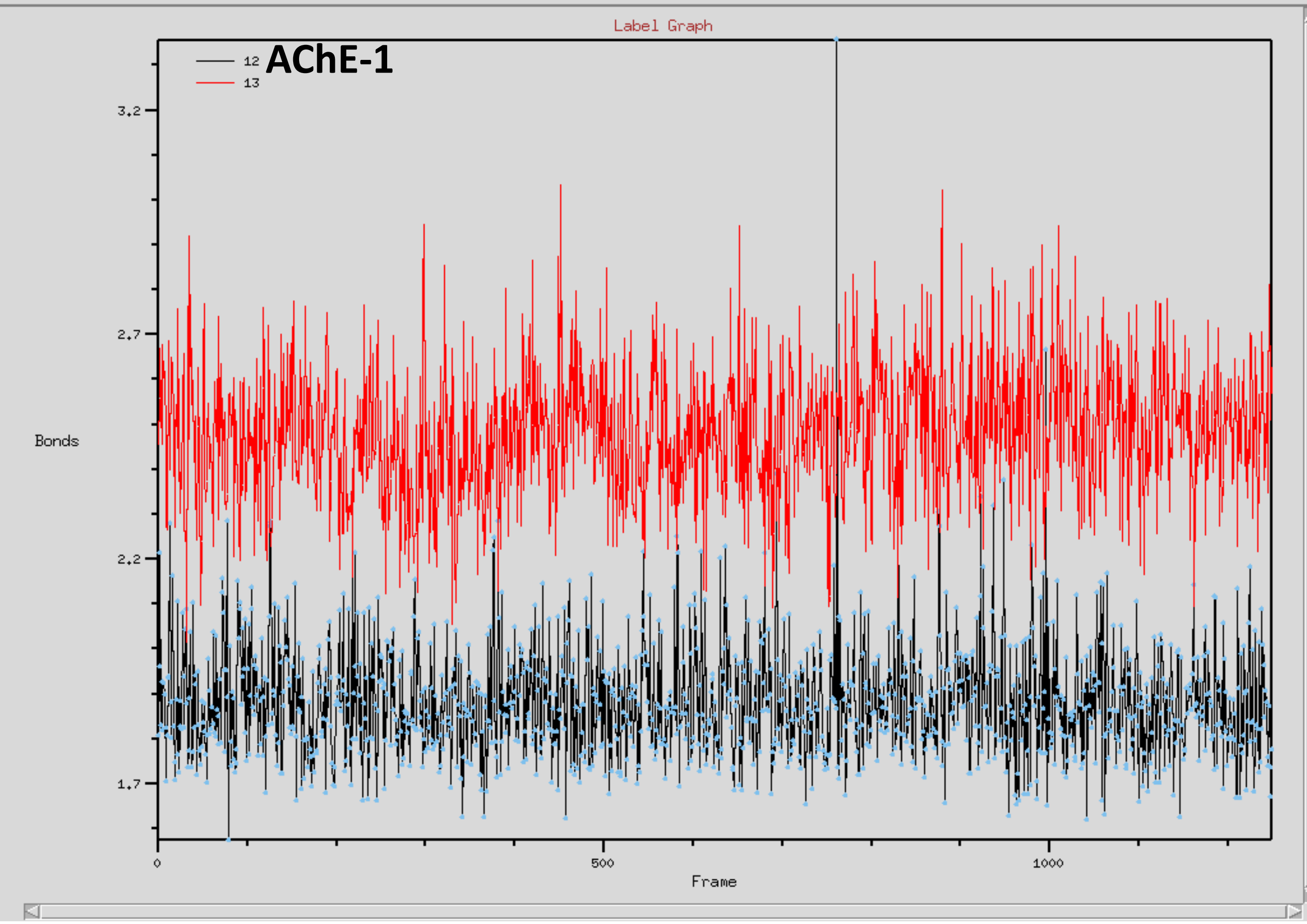
Label Graph
12
13
AChE-1
3.2
2.7
2.2
1.7
Bonds
0
500
1000
Frame

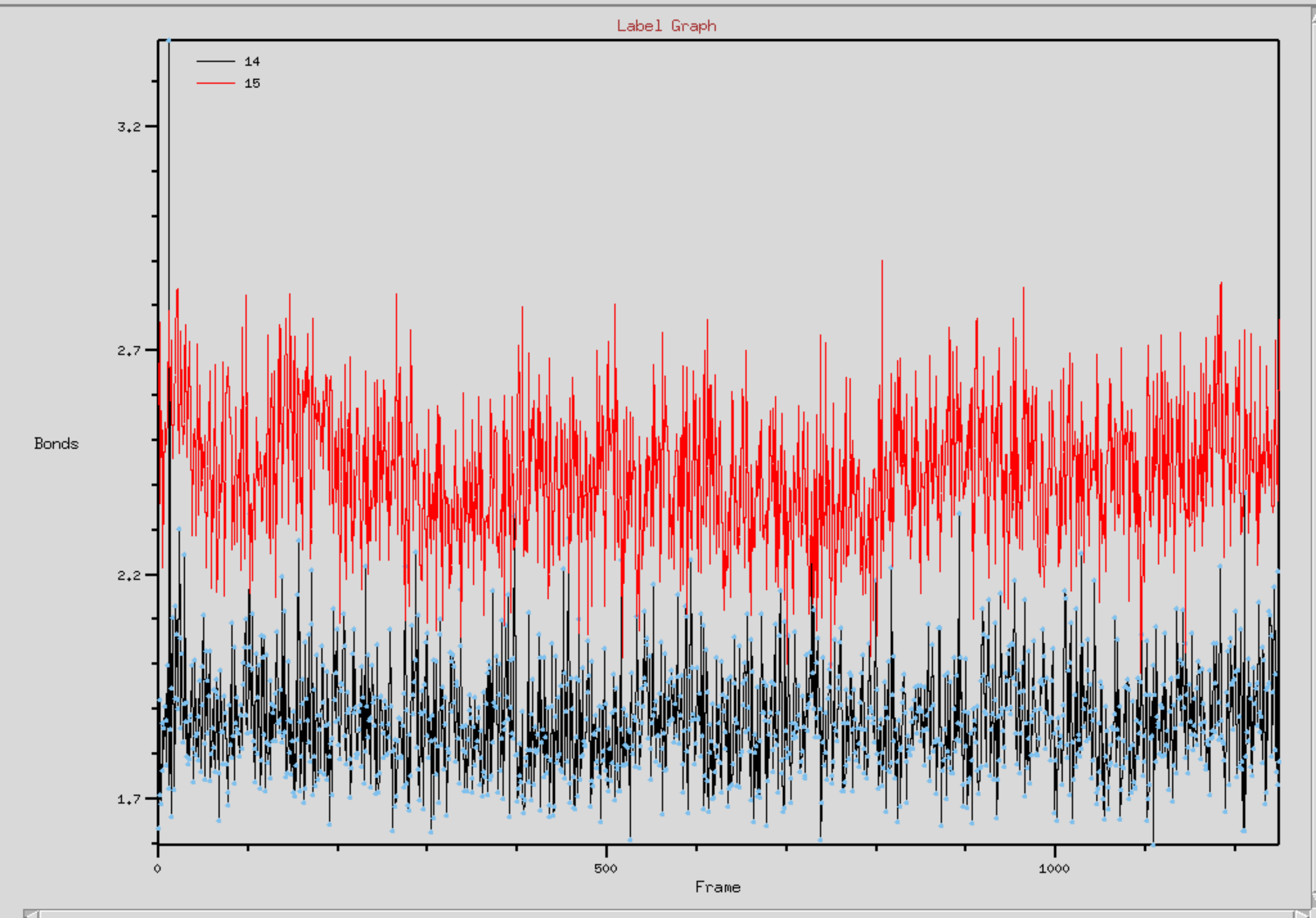
Label Graph
14
15
3.2
2.7
2.2
1.7
Bonds
0
500
1000
Frame

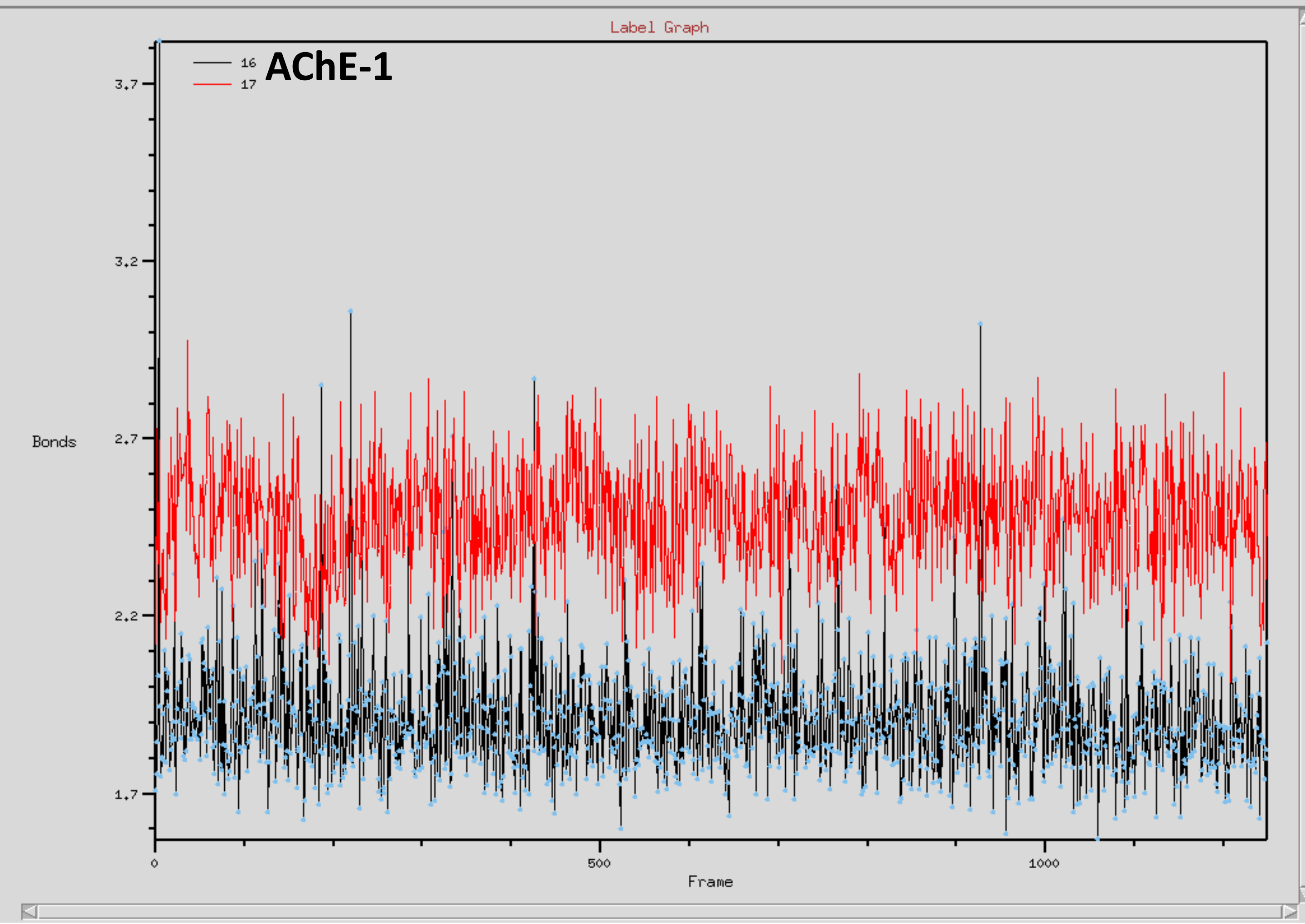

Label Graph
16
17
AChE-1
Bonds
3.7
3.2
2.7
2.2
1.7
0
500
1000
Frame

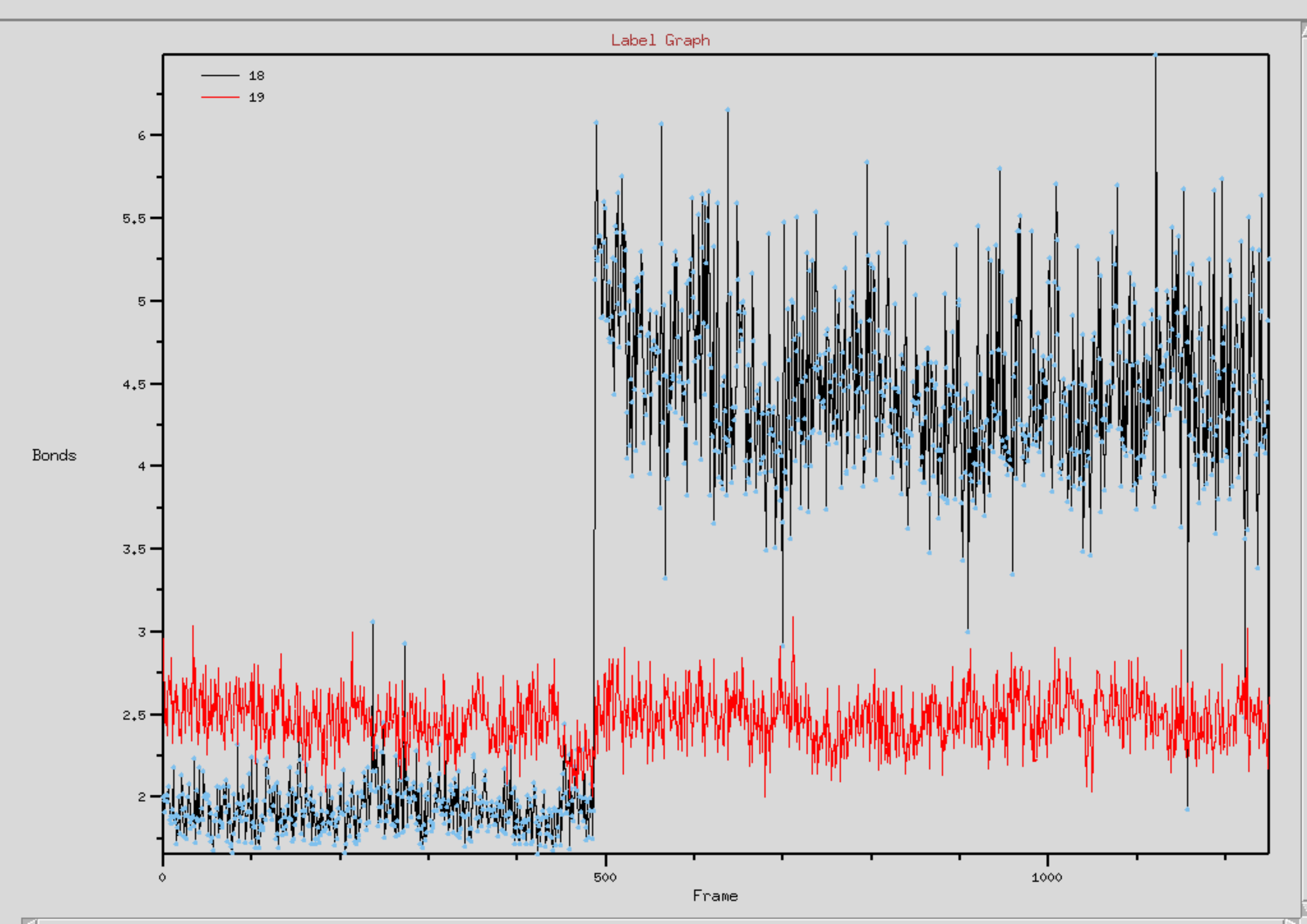

Label Graph
18
19
Bonds
6
5.5
5
4.5
4
3.5
3
2.5
2
0
500
1000
Frame

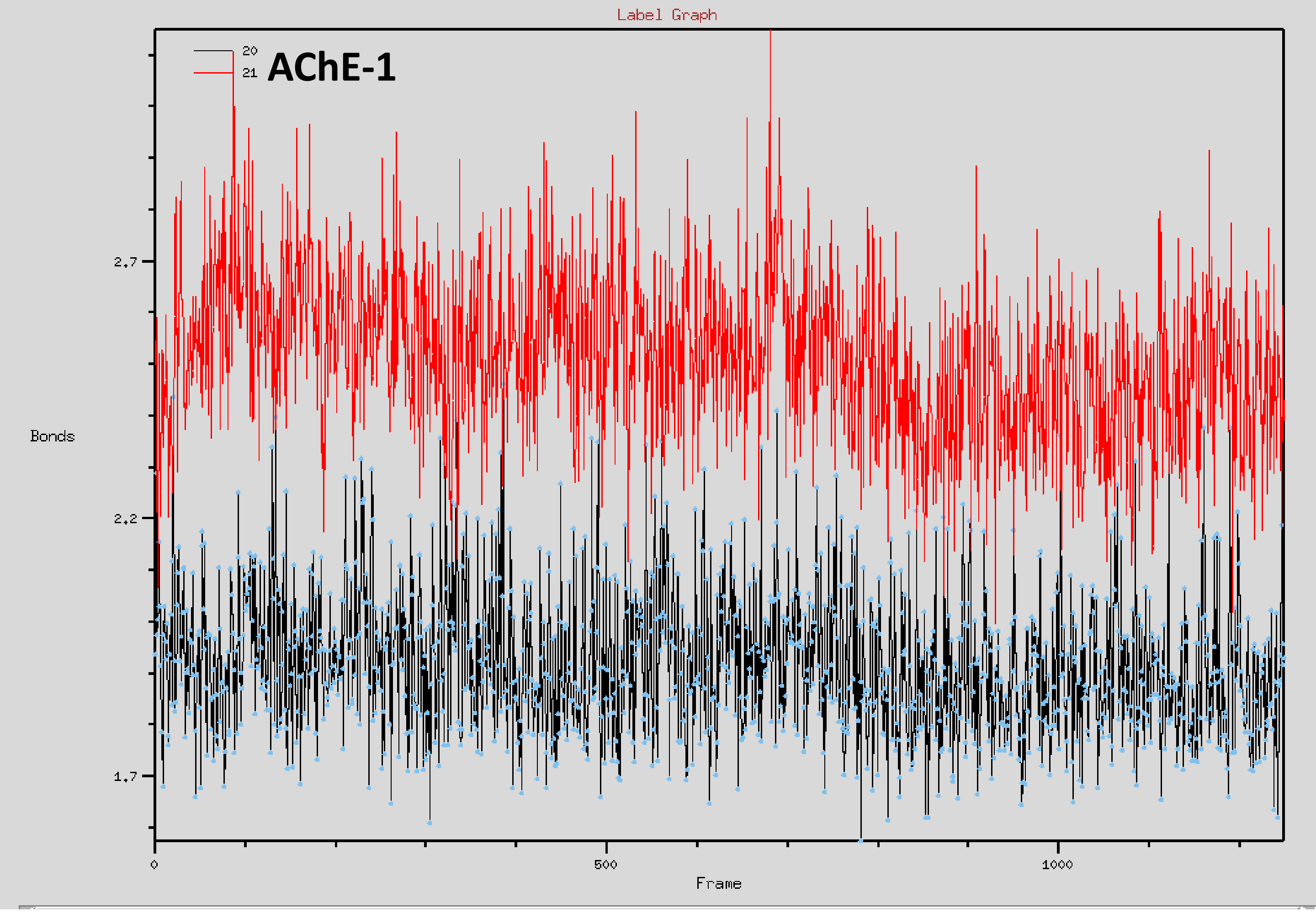
Label Graph
AChE-1
20
21
Bonds
2.7
2.2
1.7
0
500
1000
Frame

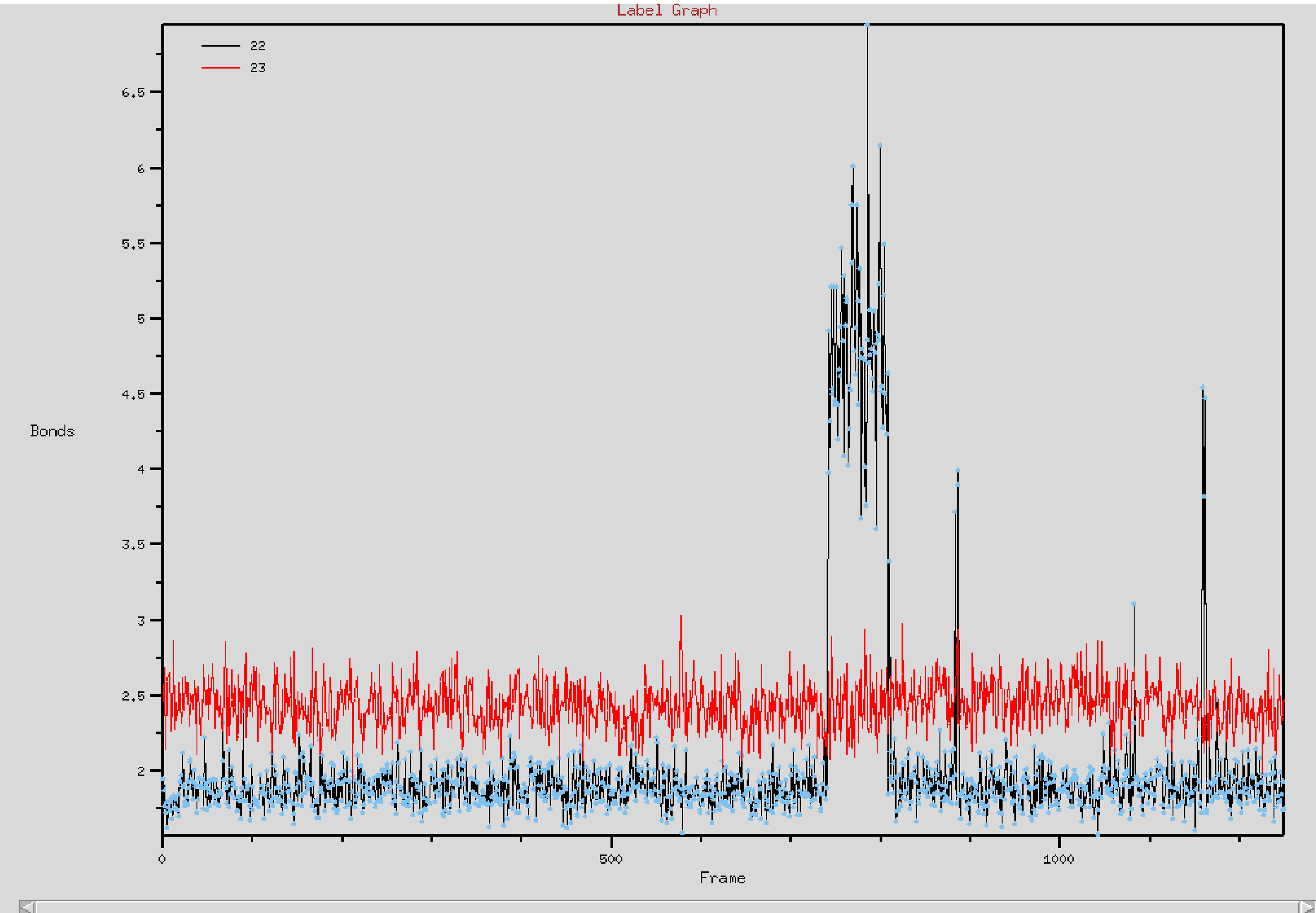
Label Graph
22
23
Bonds
6.5
6
5.5
5
4.5
4
3.5
3
2.5
2
0
500
1000
Frame

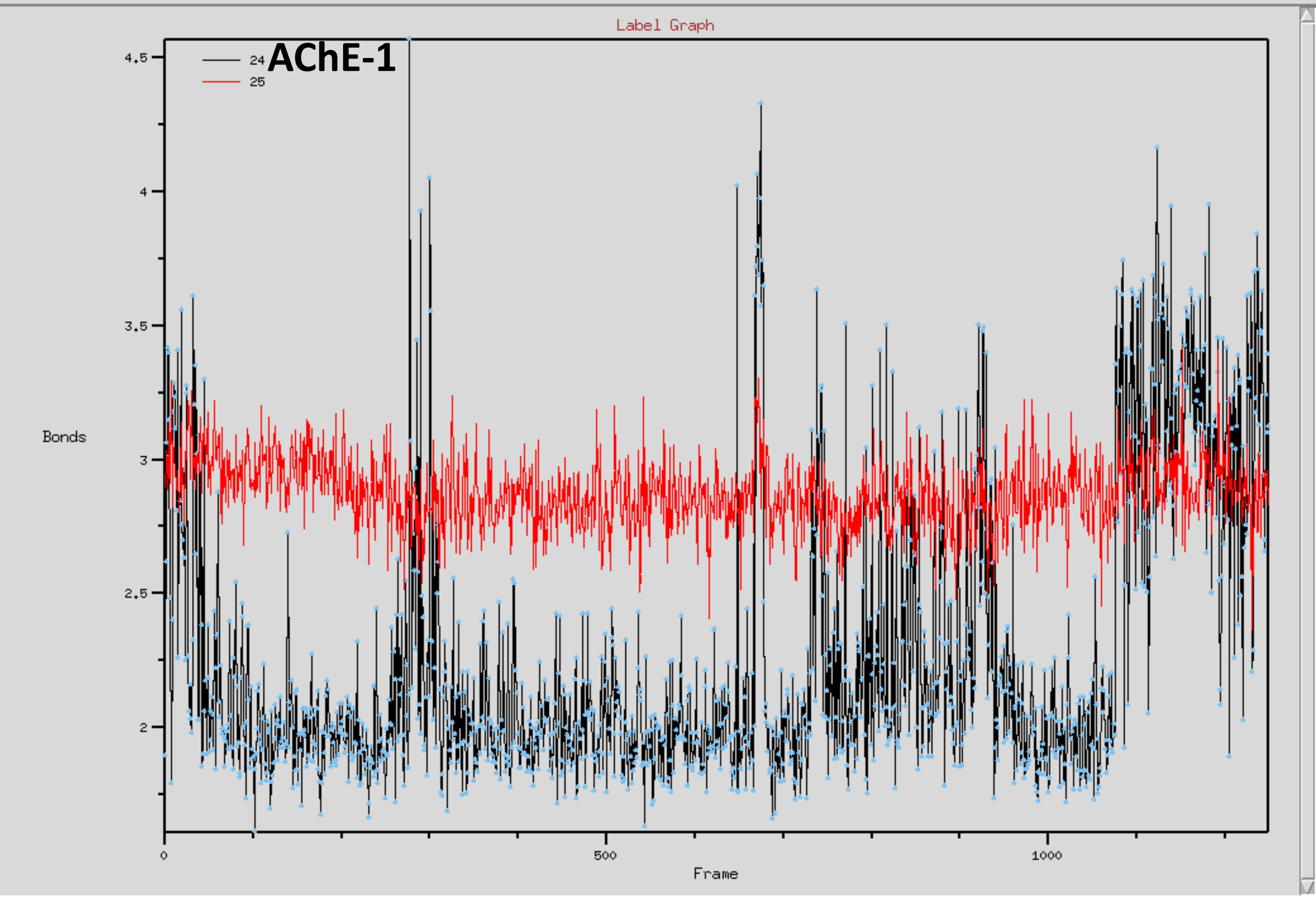
Label Graph
AChE-1
24
25
Bonds
Frame
4.5
4
3.5
3
2.5
2
0
500
1000

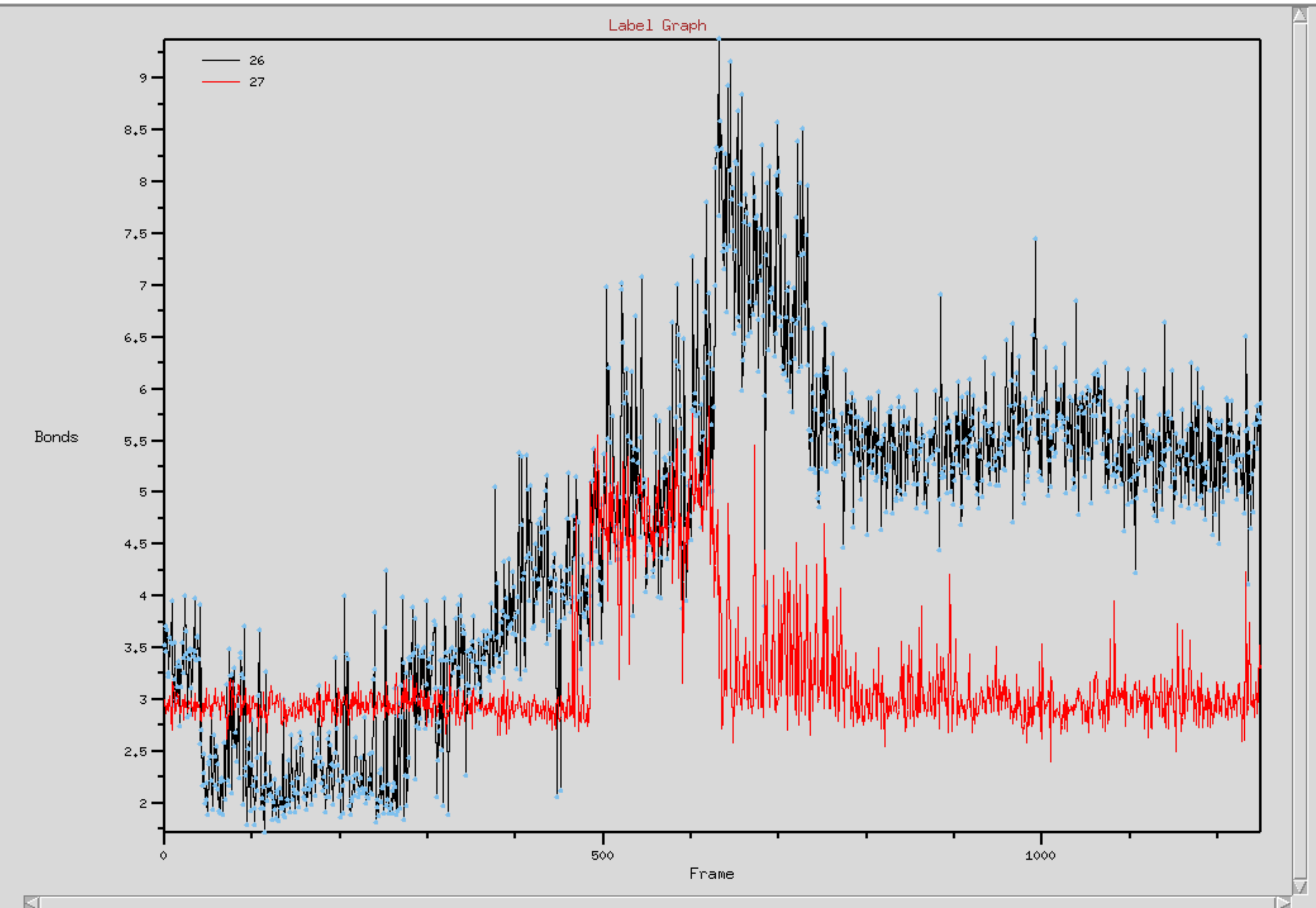
Label Graph
26
27
Bonds
Frame
9
8.5
8
7.5
7
6.5
6
5.5
5
4.5
4
3.5
3
2.5
2
0
500
1000

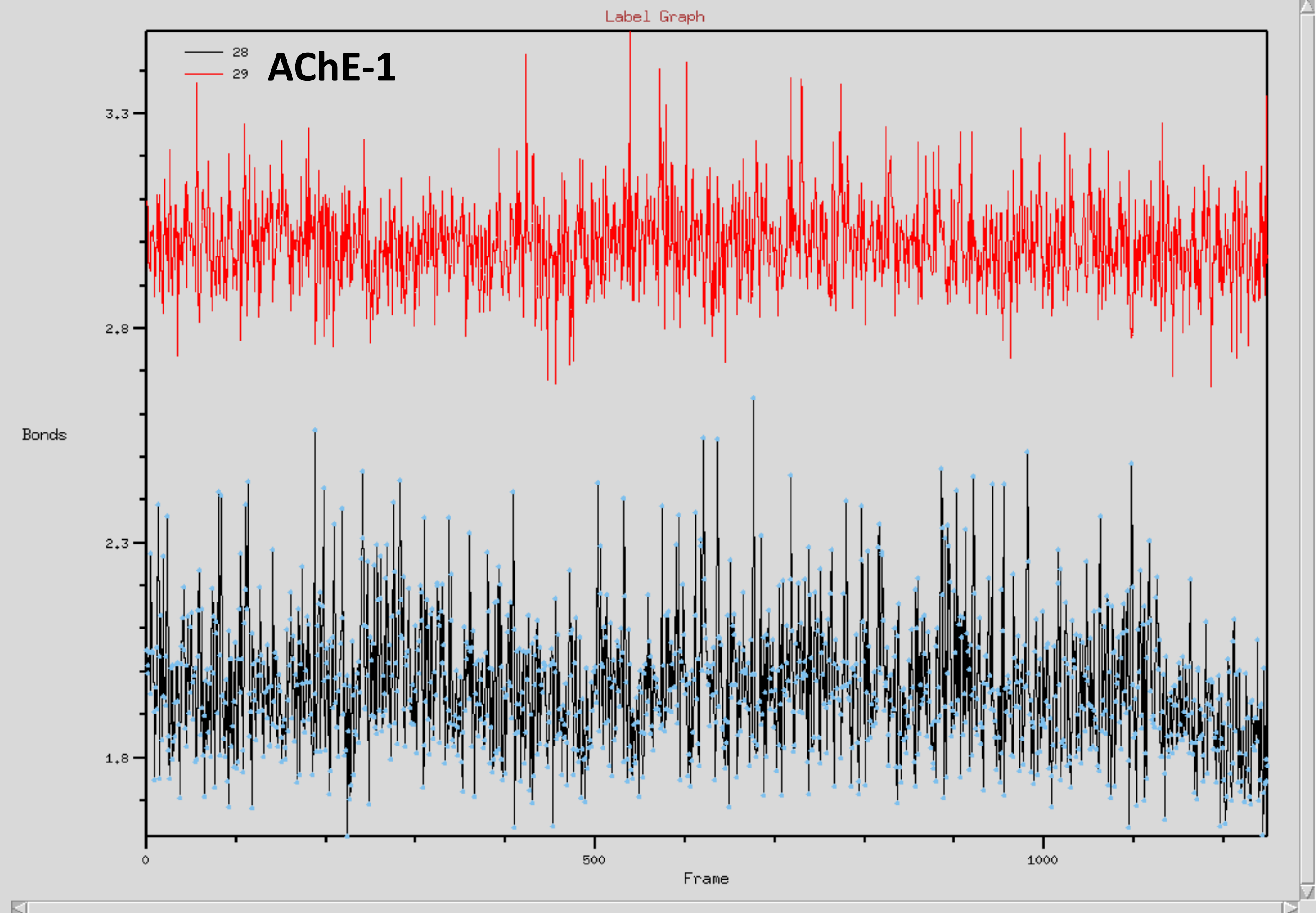

Label Graph
28
29
AChE-1
3.3
2.8
2.3
1.8
Bonds
0
500
1000
Frame

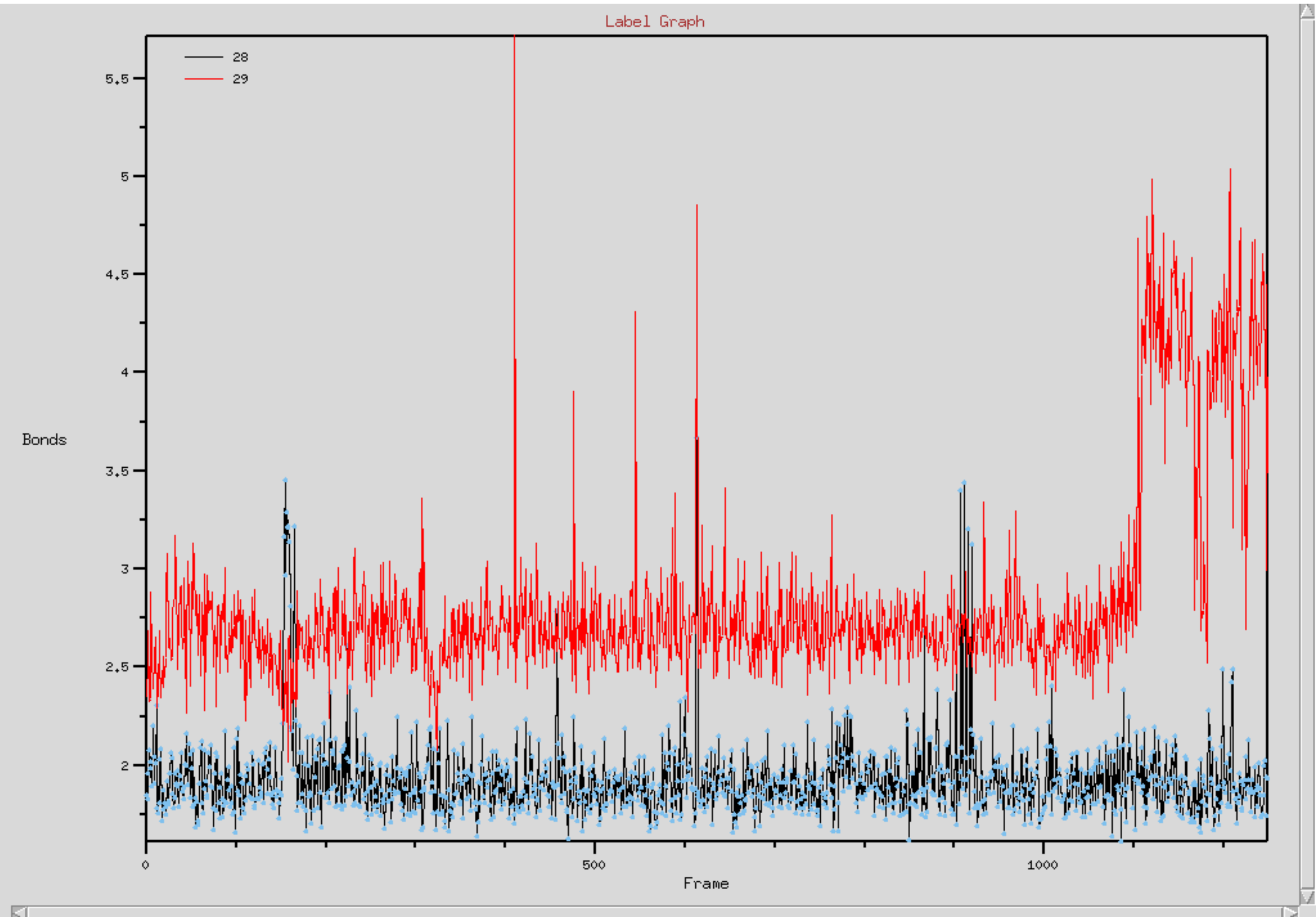

Label Graph
28
29
5.5
5
4.5
4
3.5
3
2.5
2
Bonds
0
500
1000
Frame

AChE-AC-1

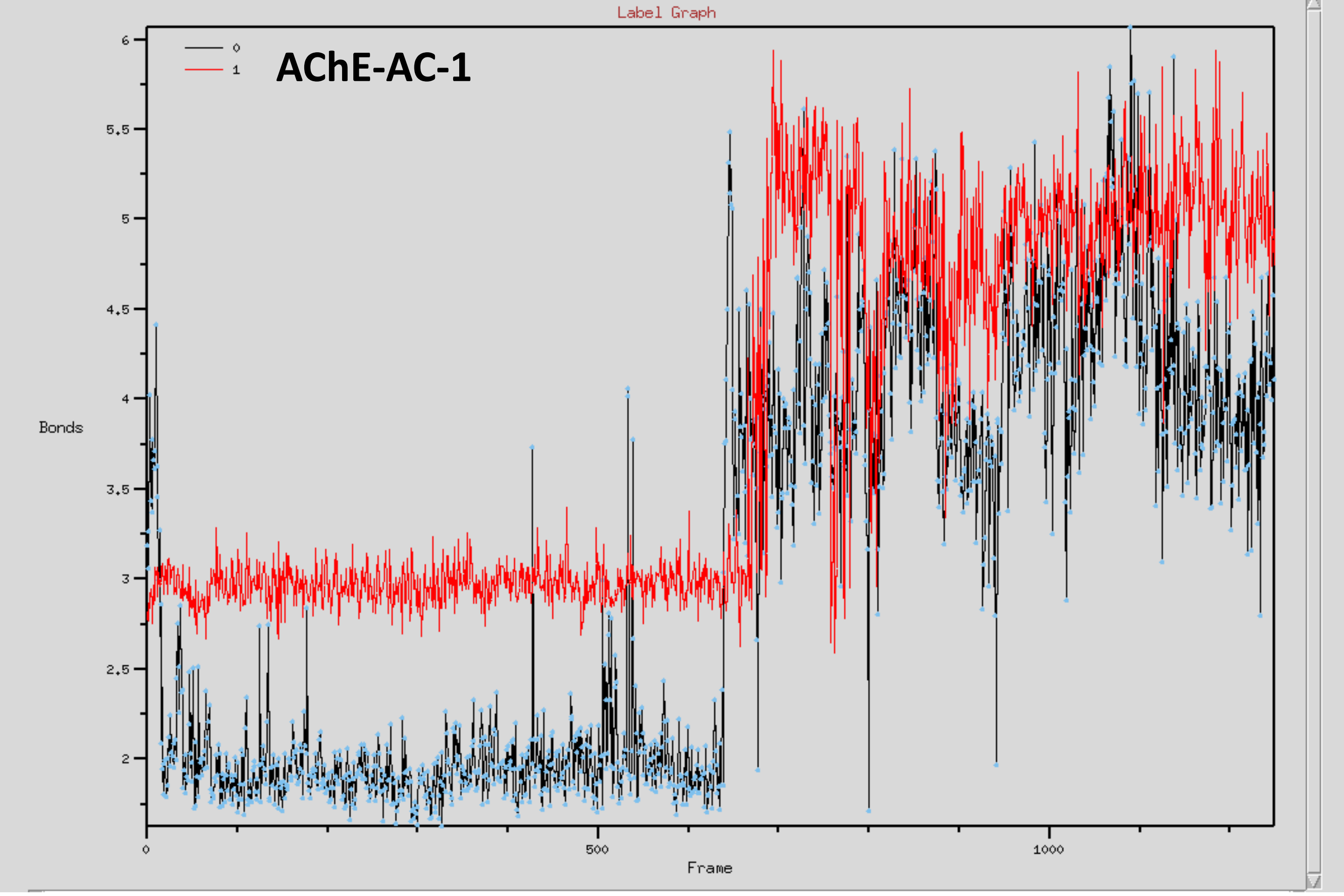

Label Graph
AChE-AC-1
0
1
Bonds
Frame
6
5.5
5
4.5
4
3.5
3
2.5
2
0
500
1000

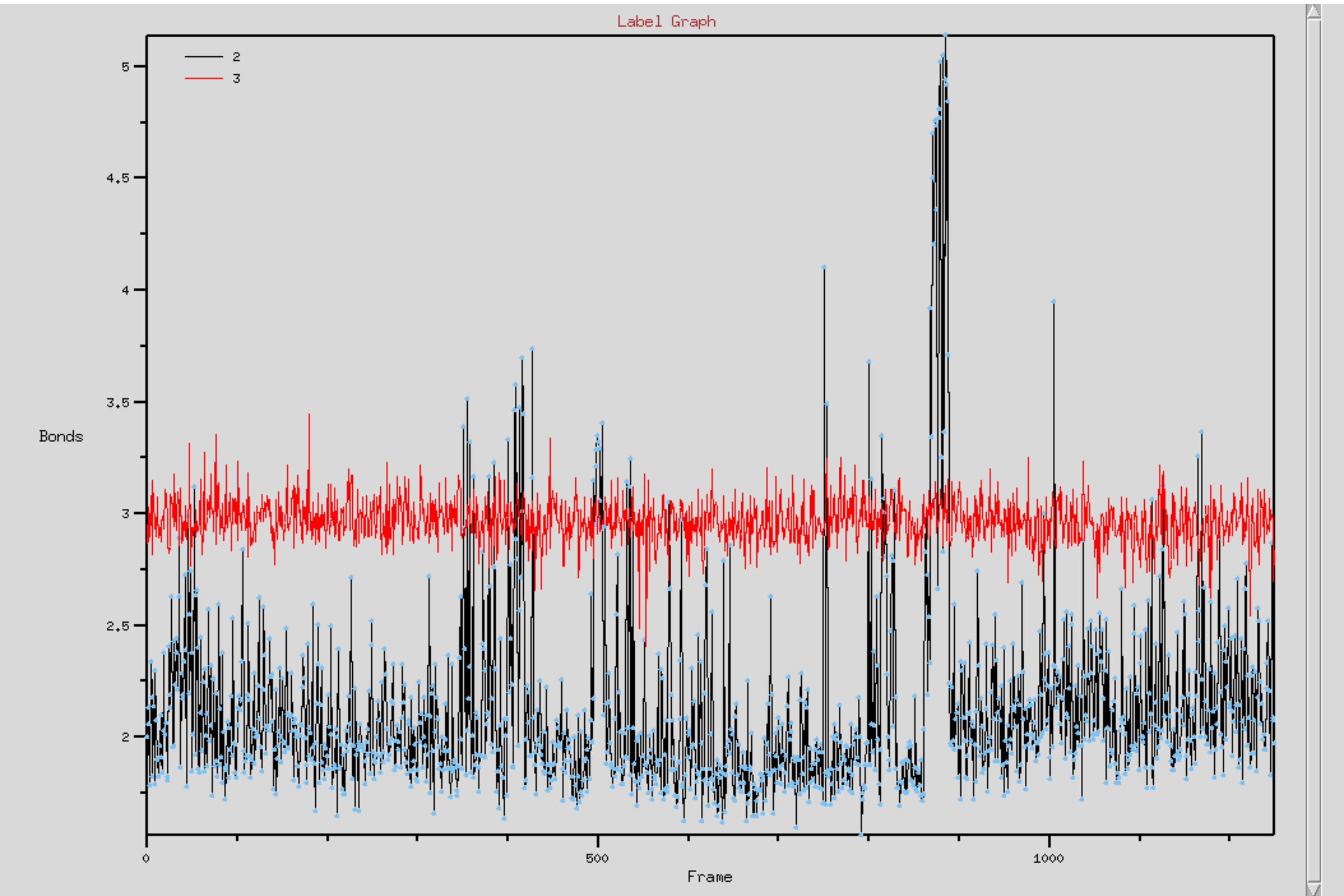

Label Graph
2
3
Bonds
Frame
5
4.5
4
3.5
3
2.5
2
0
500
1000

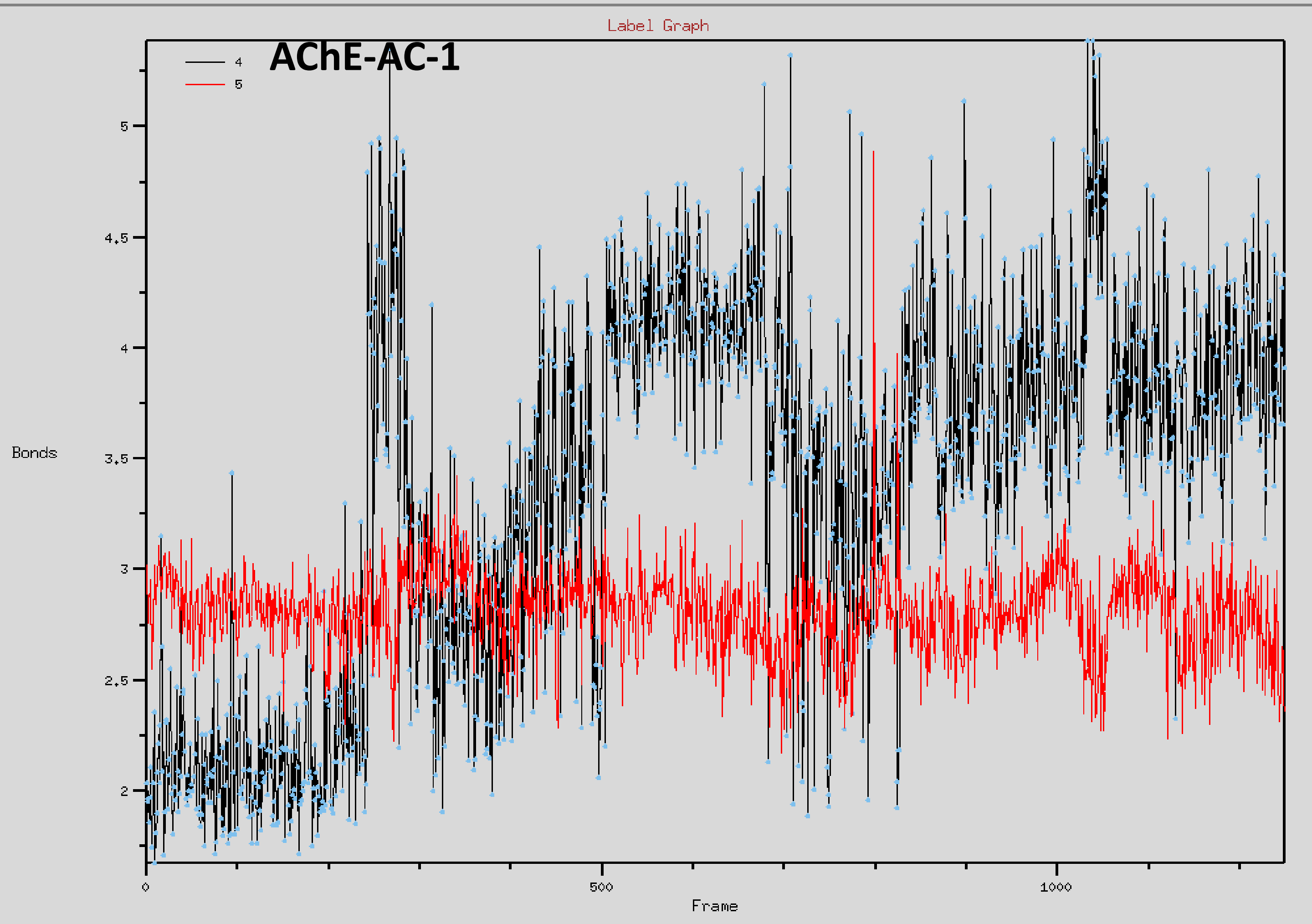
Label Graph
ACHE-AC-1
4
5
Bonds
5
4,5
4
3,5
3
2,5
2
0
500
1000
Frame

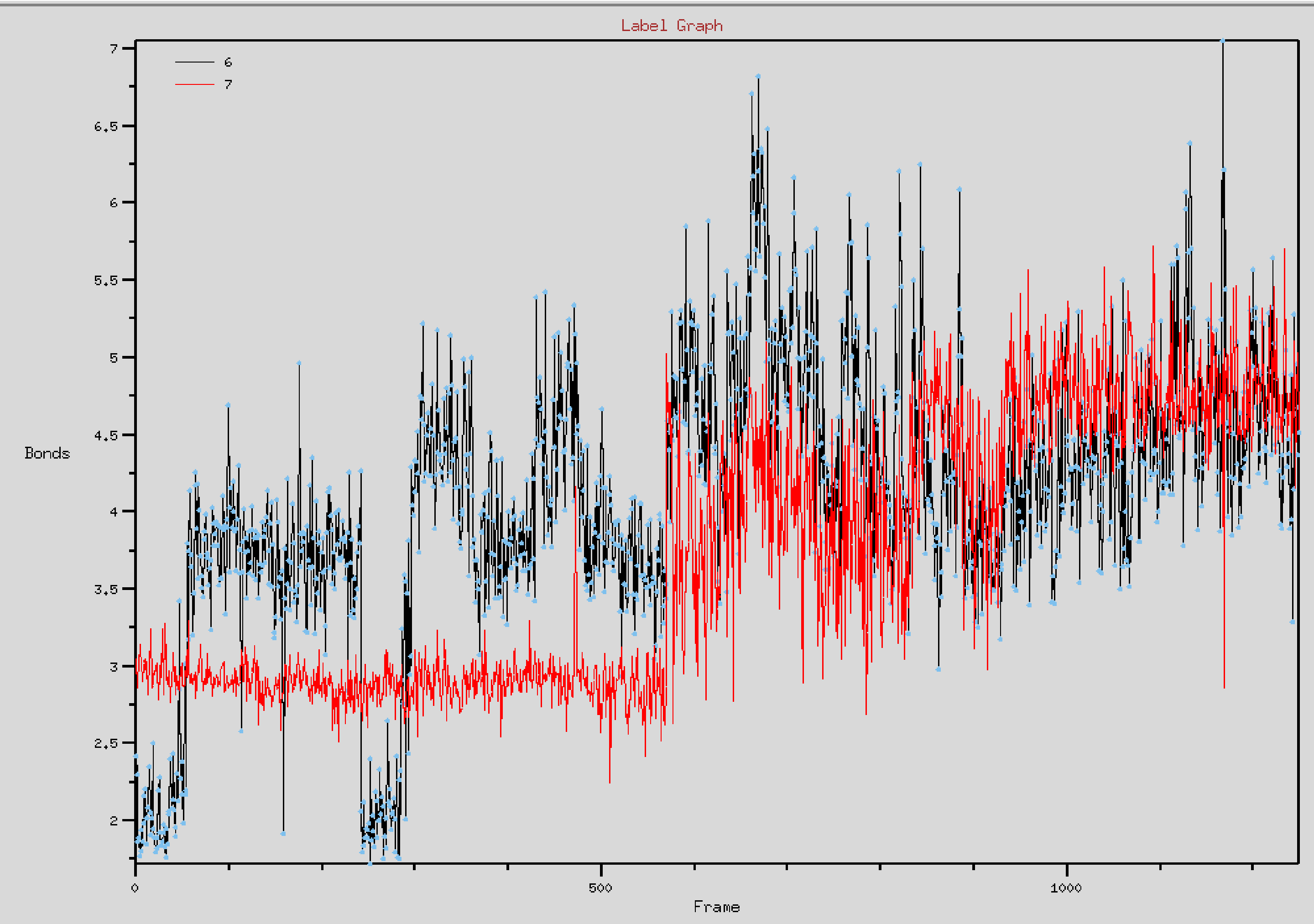
Label Graph
6
7
Bonds
7
6,5
6
5,5
5
4,5
4
3,5
3
2,5
2
0
500
1000
Frame

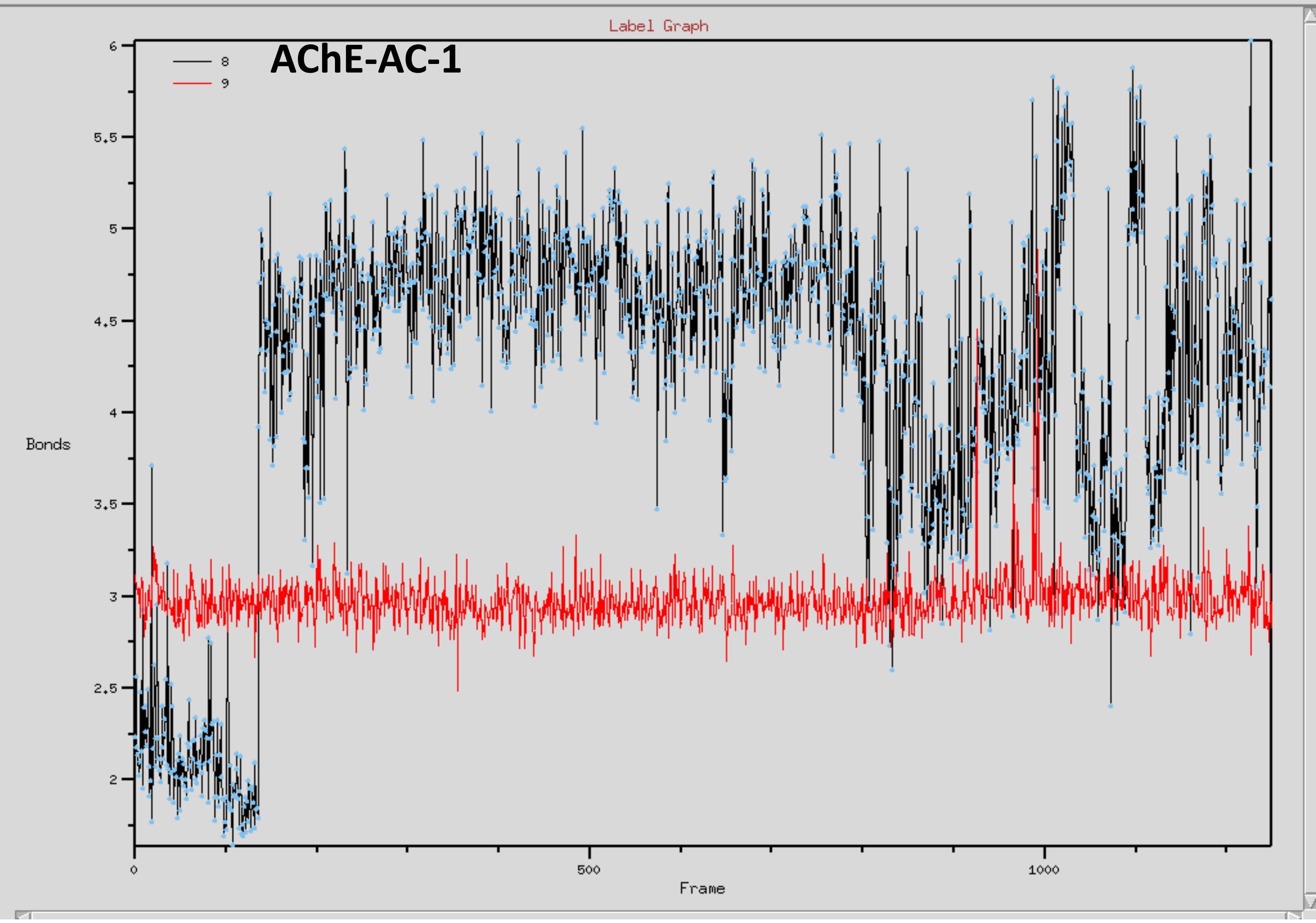
Label Graph
AChE-AC-1
8
9
Bonds
Frame
0
500
1000
6
5.5
5
4.5
4
3.5
3
2.5
2

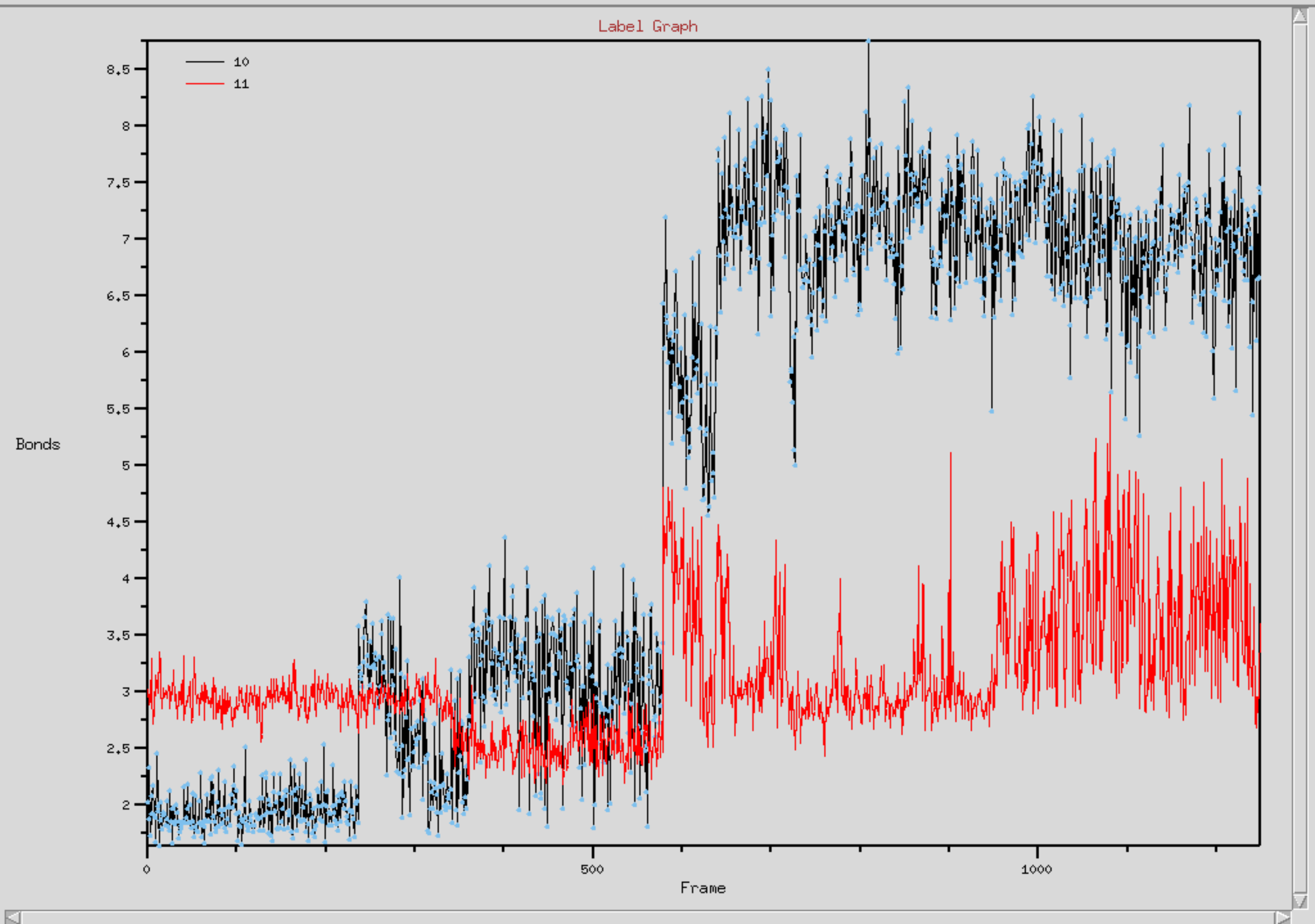
Label Graph
10
11
Bonds
Frame
0
500
1000
8.5
8
7.5
7
6.5
6
5.5
5
4.5
4
3.5
3
2.5
2

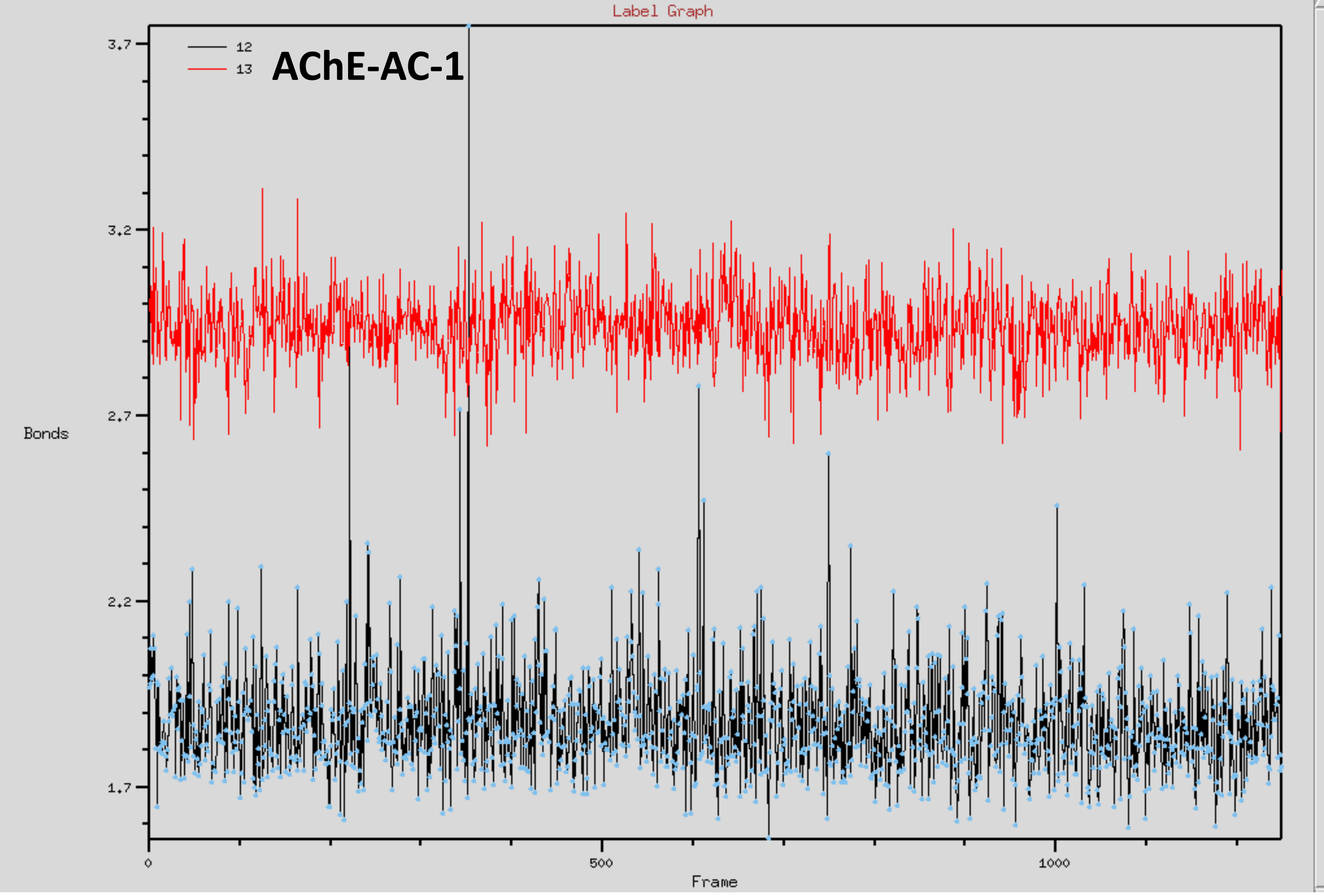

Label Graph
12
13
AChE-AC-1
3.7
3.2
2.7
2.2
1.7
Bonds
0
500
1000
Frame

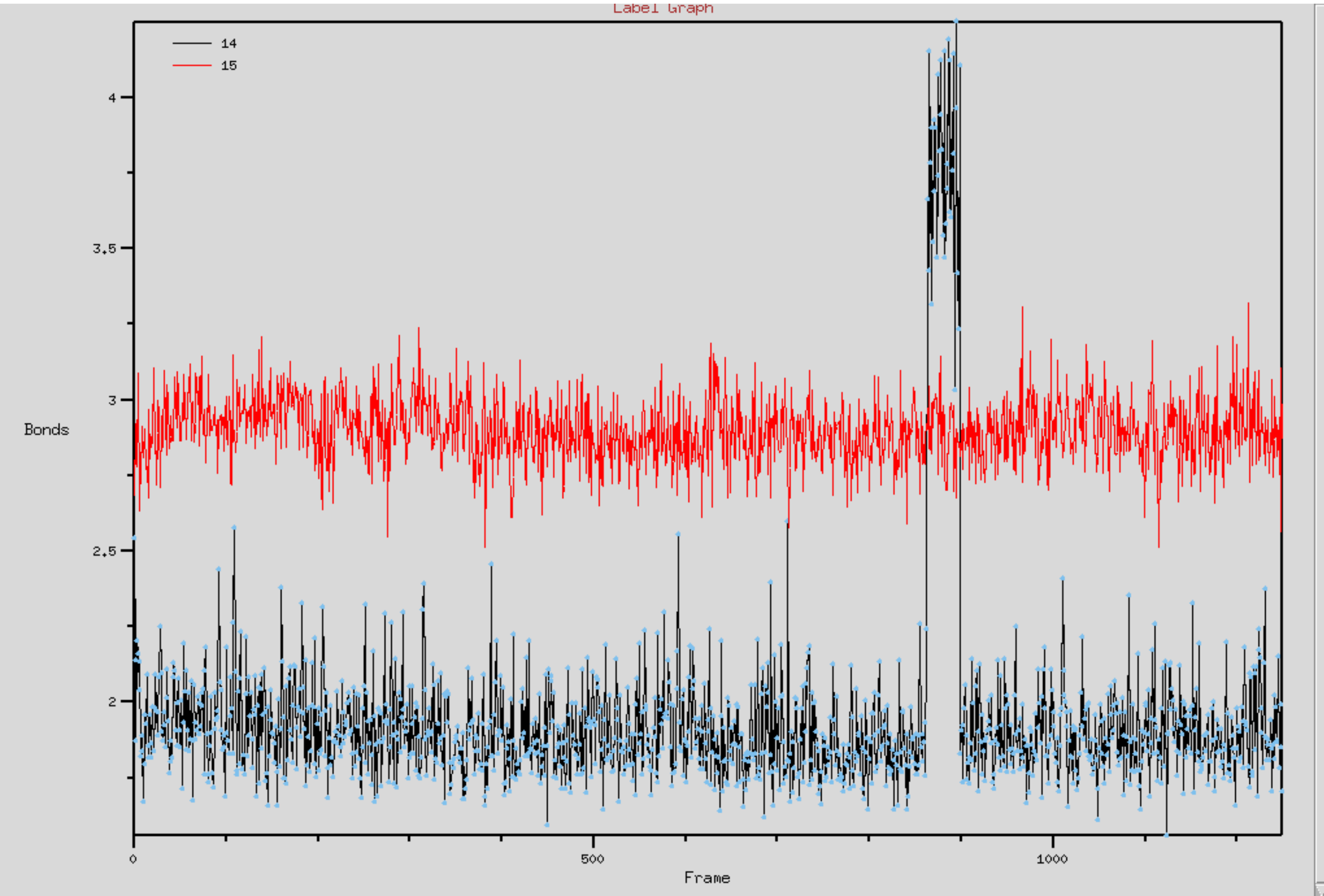

Label Graph
14
15
4
3.5
3
2.5
2
Bonds
0
500
1000
Frame

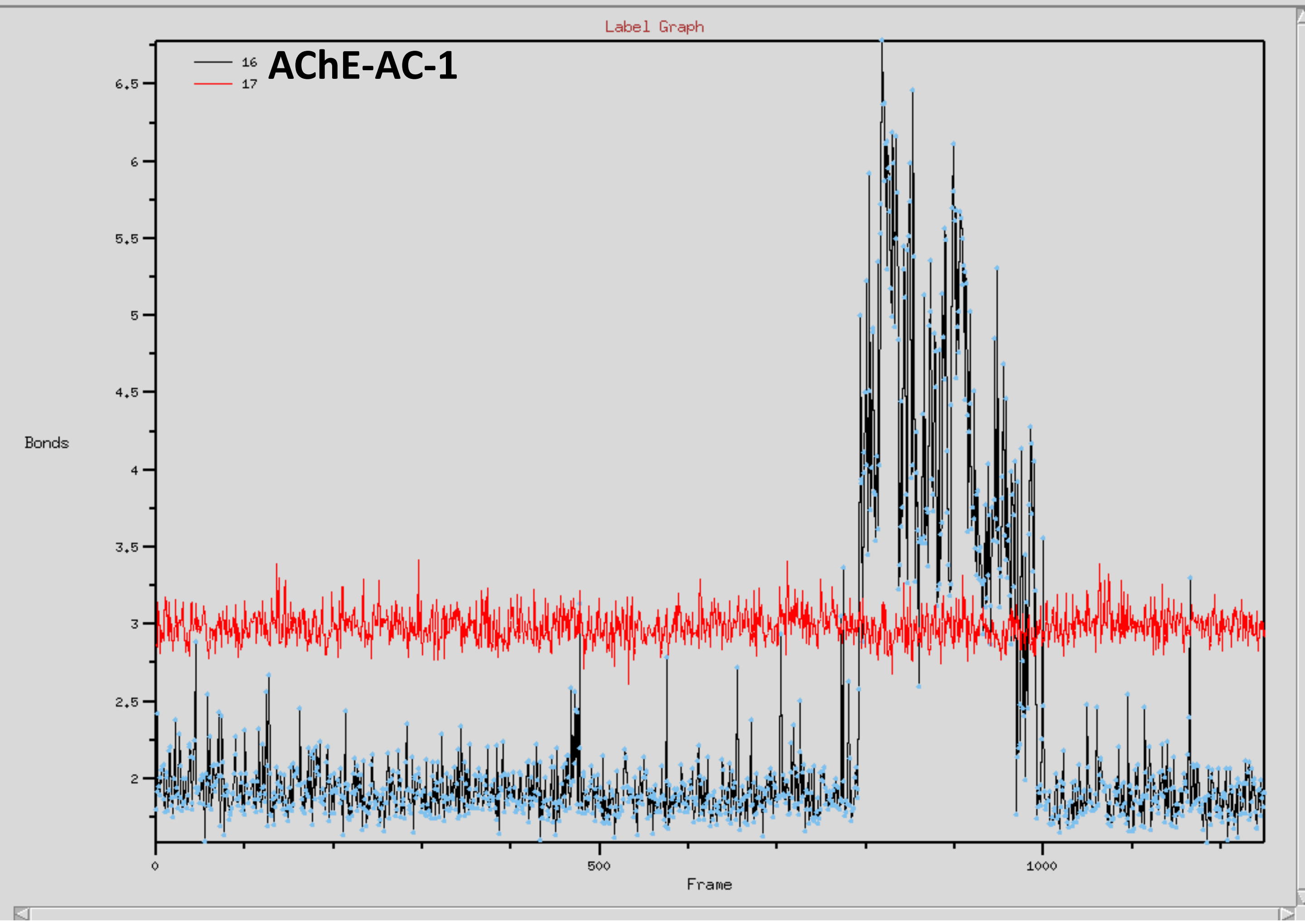
Label Graph
16
17
AChE-AC-1
Bonds
Frame
0
500
1000
6.5
6
5.5
5
4.5
4
3.5
3
2.5
2

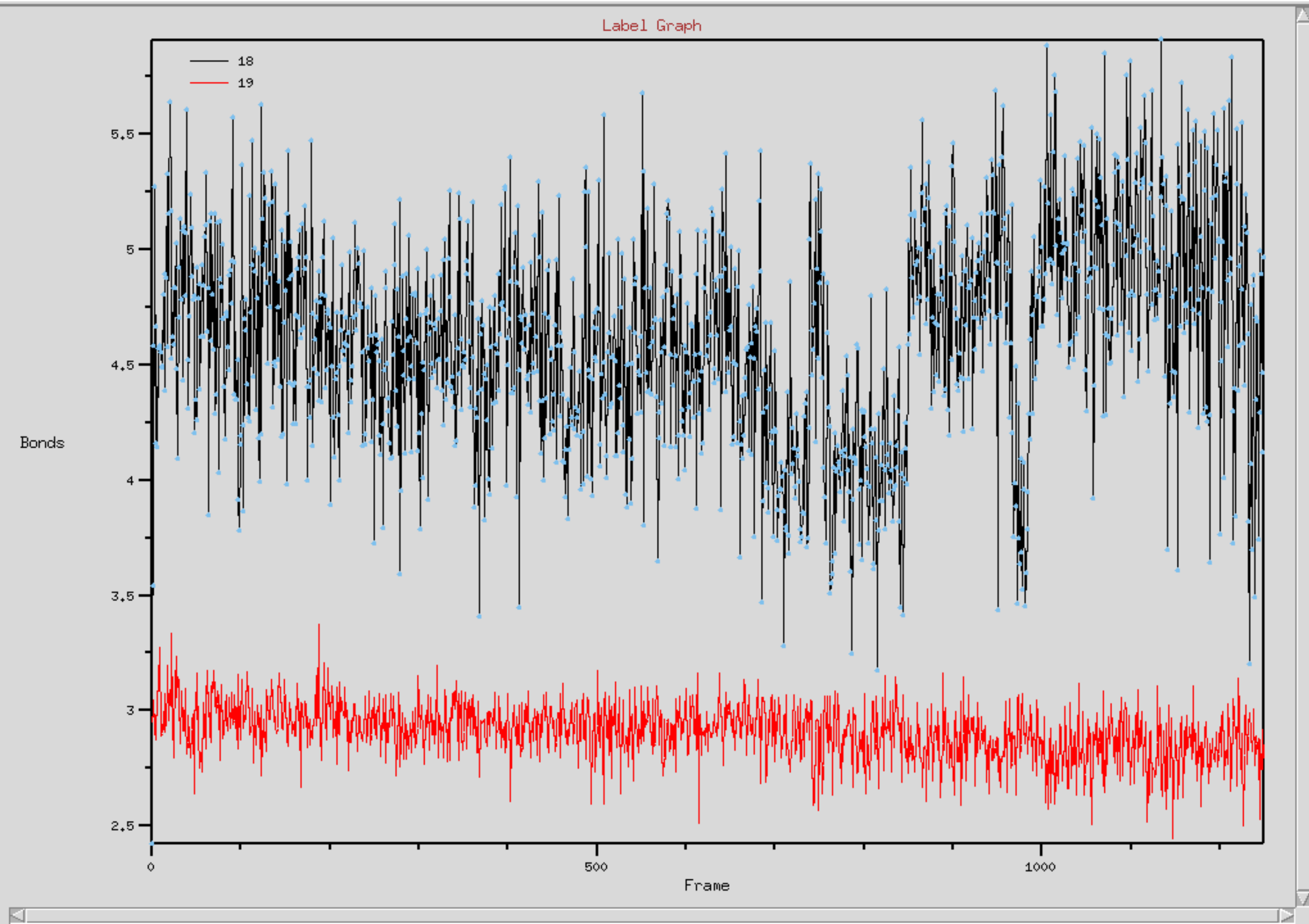
Label Graph
18
19
Bonds
Frame
0
500
1000
5.5
5
4.5
4
3.5
3
2.5

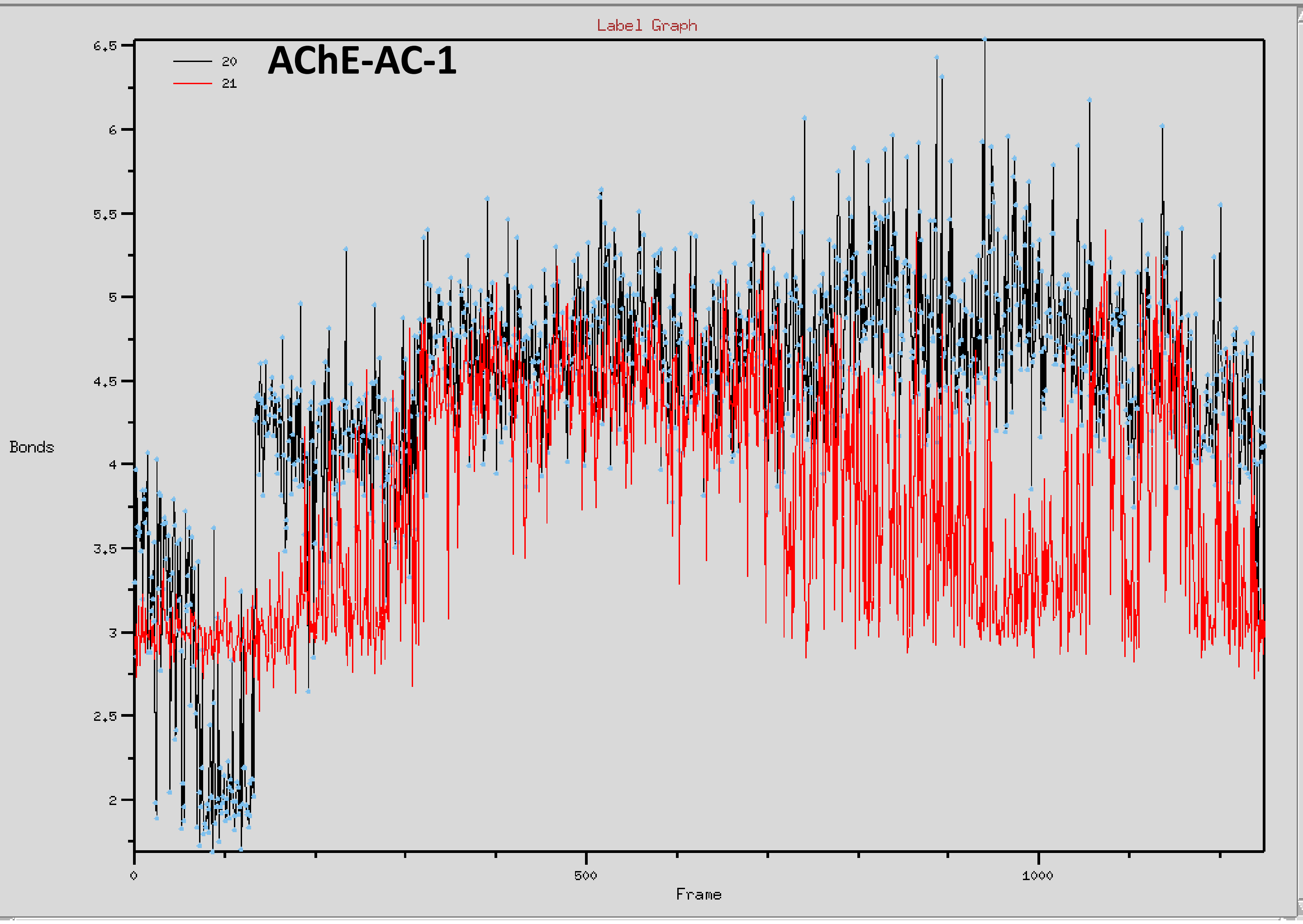
Label Graph
AChE-AC-1
20
21
Bonds
Frame
0
500
1000
6,5
6
5,5
5
4,5
4
3,5
3
2,5
2

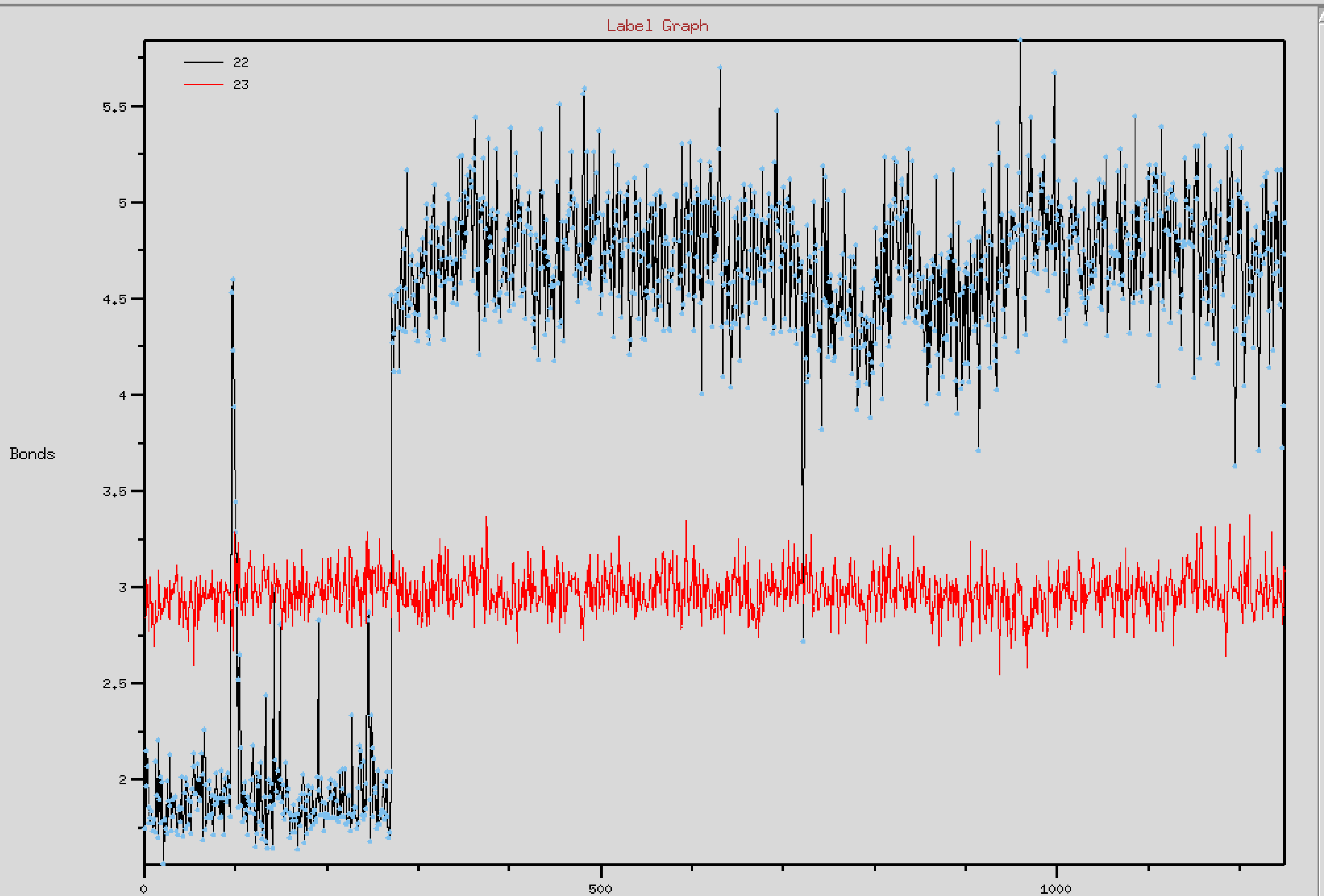
Label Graph
22
23
Bonds
0
500
1000
5,5
5
4,5
4
3,5
3
2,5
2

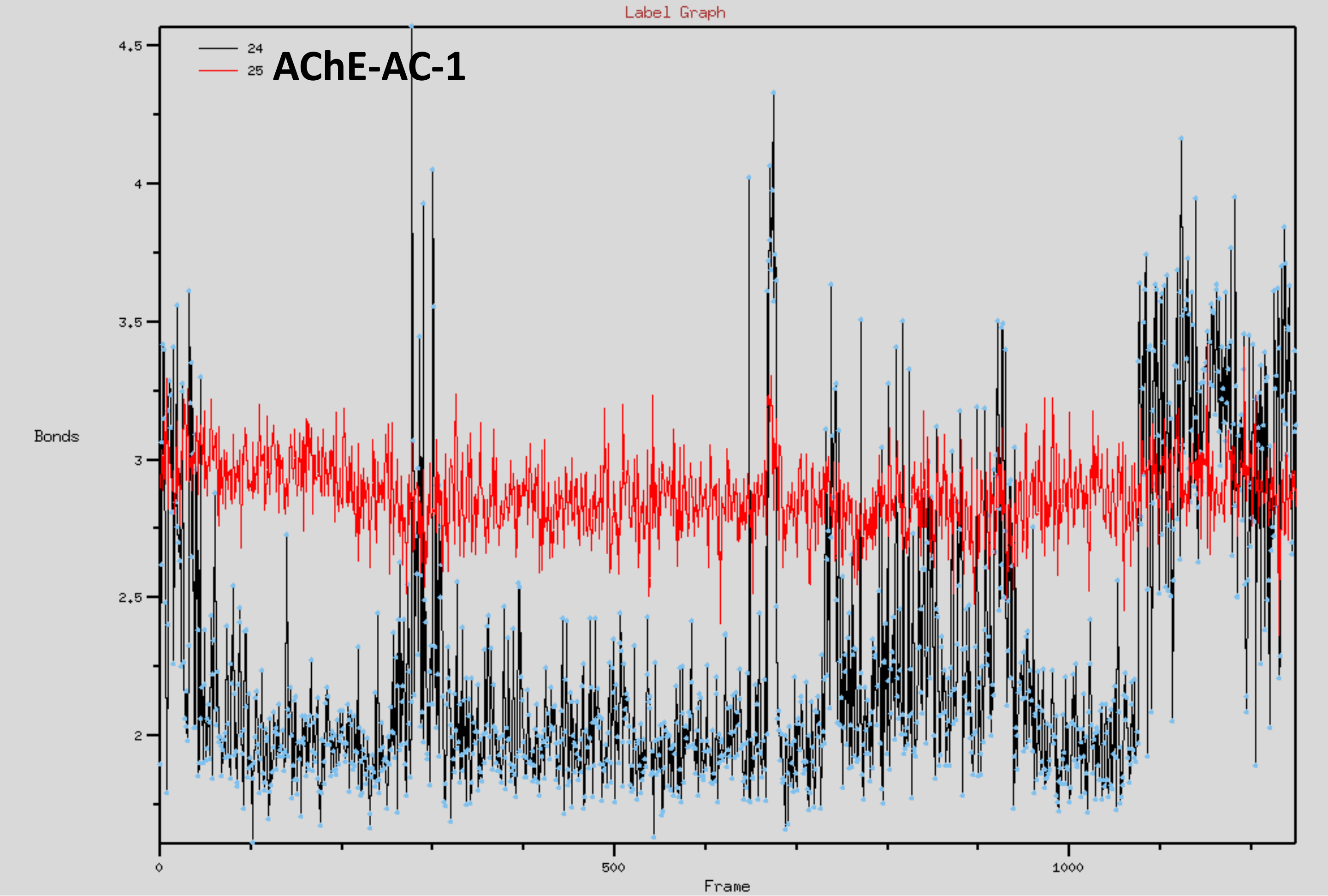
Label Graph
24
25
AChE-AC-1
Bonds
Frame
4.5
4
3.5
3
2.5
2
0
500
1000

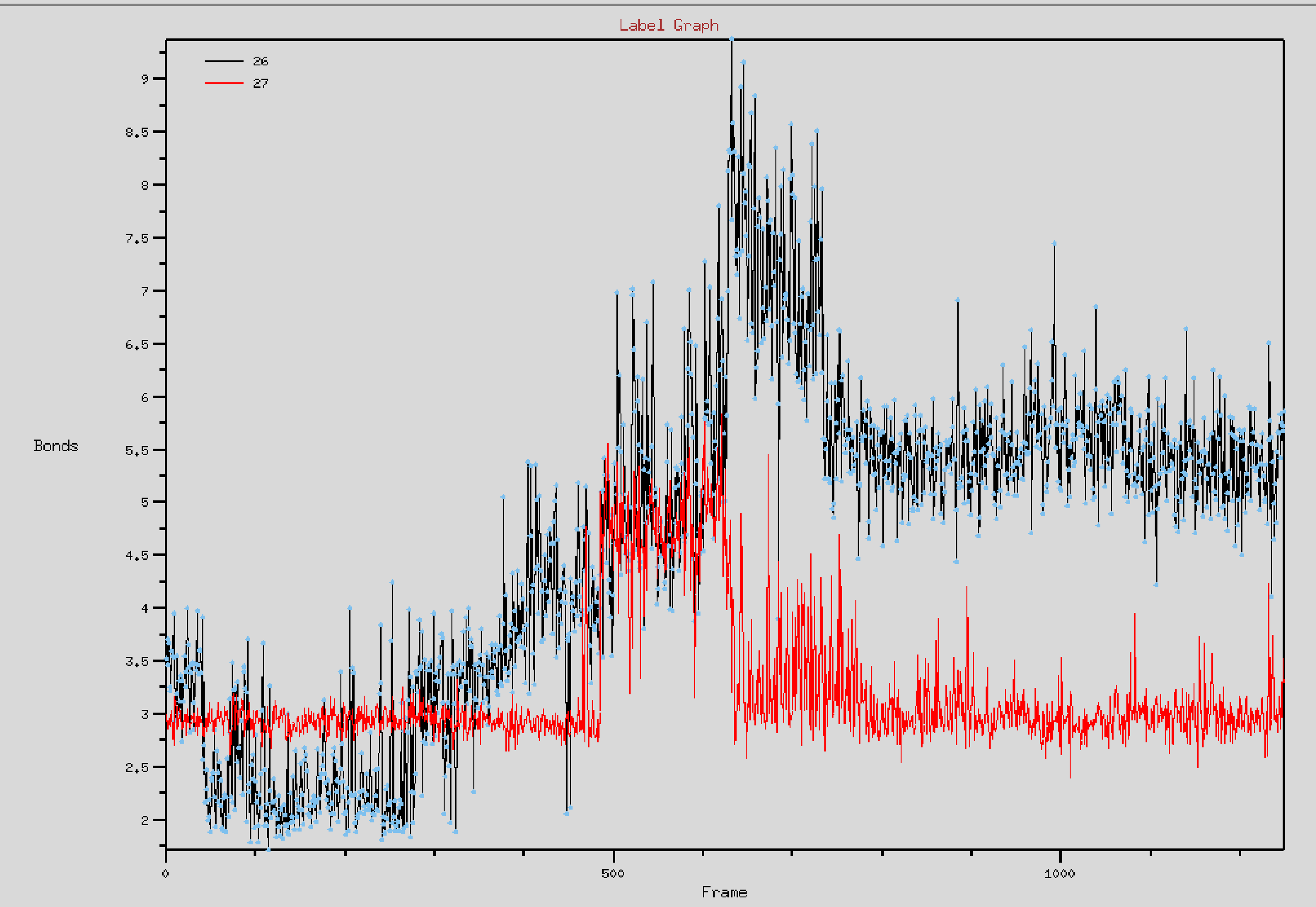
Label Graph
26
27
Bonds
Frame
9
8.5
8
7.5
7
6.5
6
5.5
5
4.5
4
3.5
3
2.5
2
0
500
1000

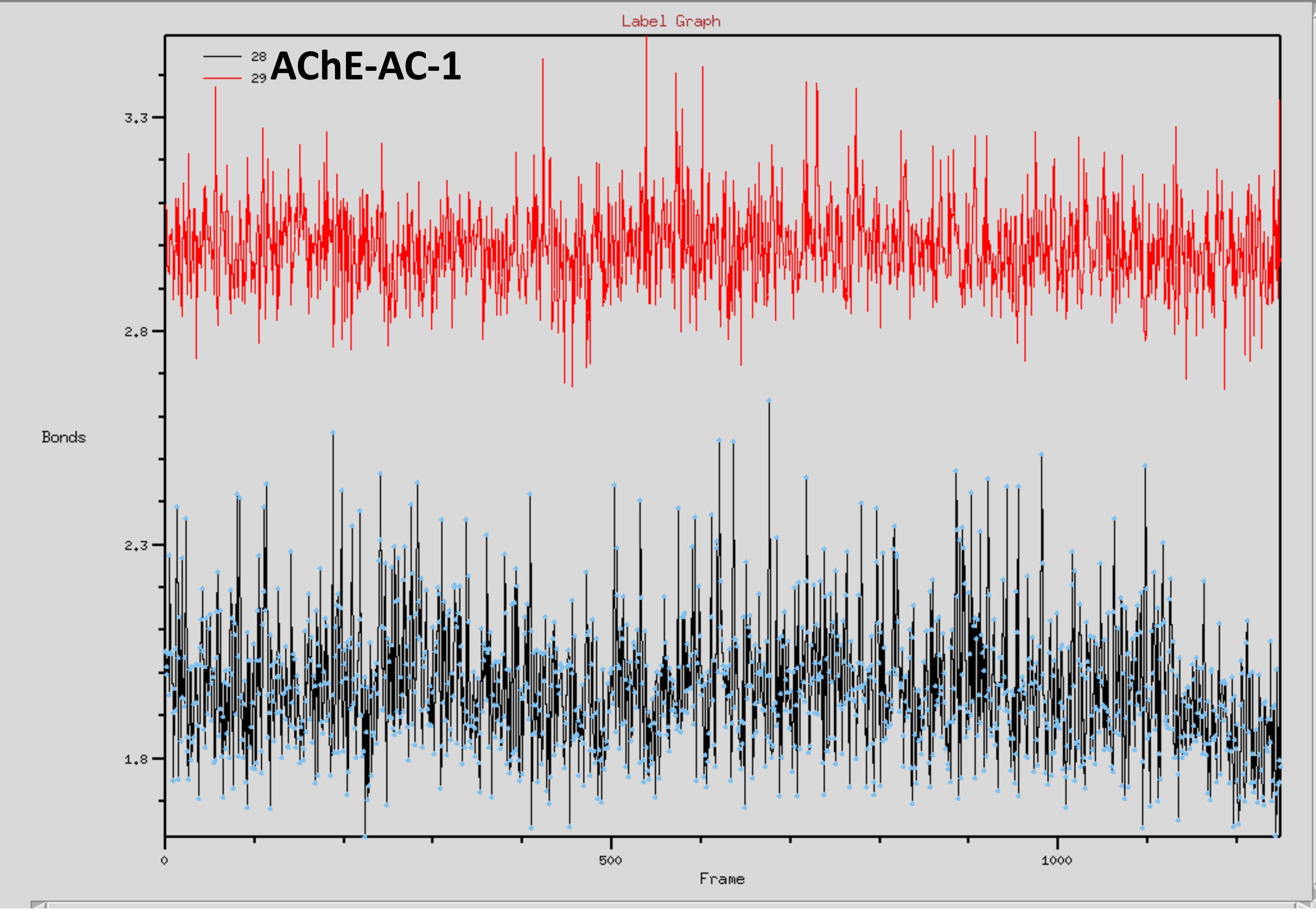

Label Graph
28
29
AChE-AC-1
3.3
2.8
2.3
1.8
Bonds
0
500
1000
Frame

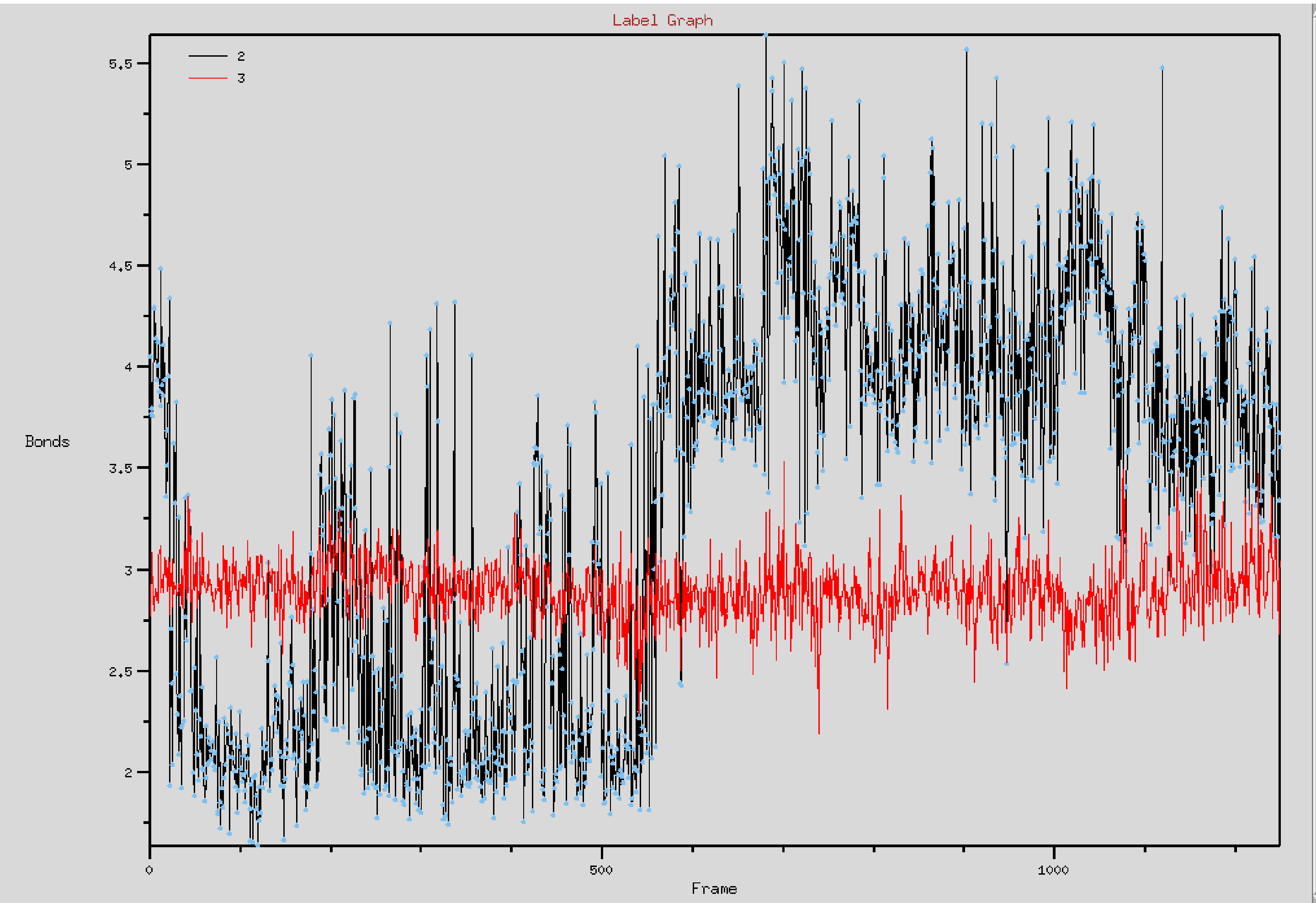

Label Graph
2
3
5.5
5
4.5
4
3.5
3
2.5
2
Bonds
0
500
1000
Frame

AChE-AC-2

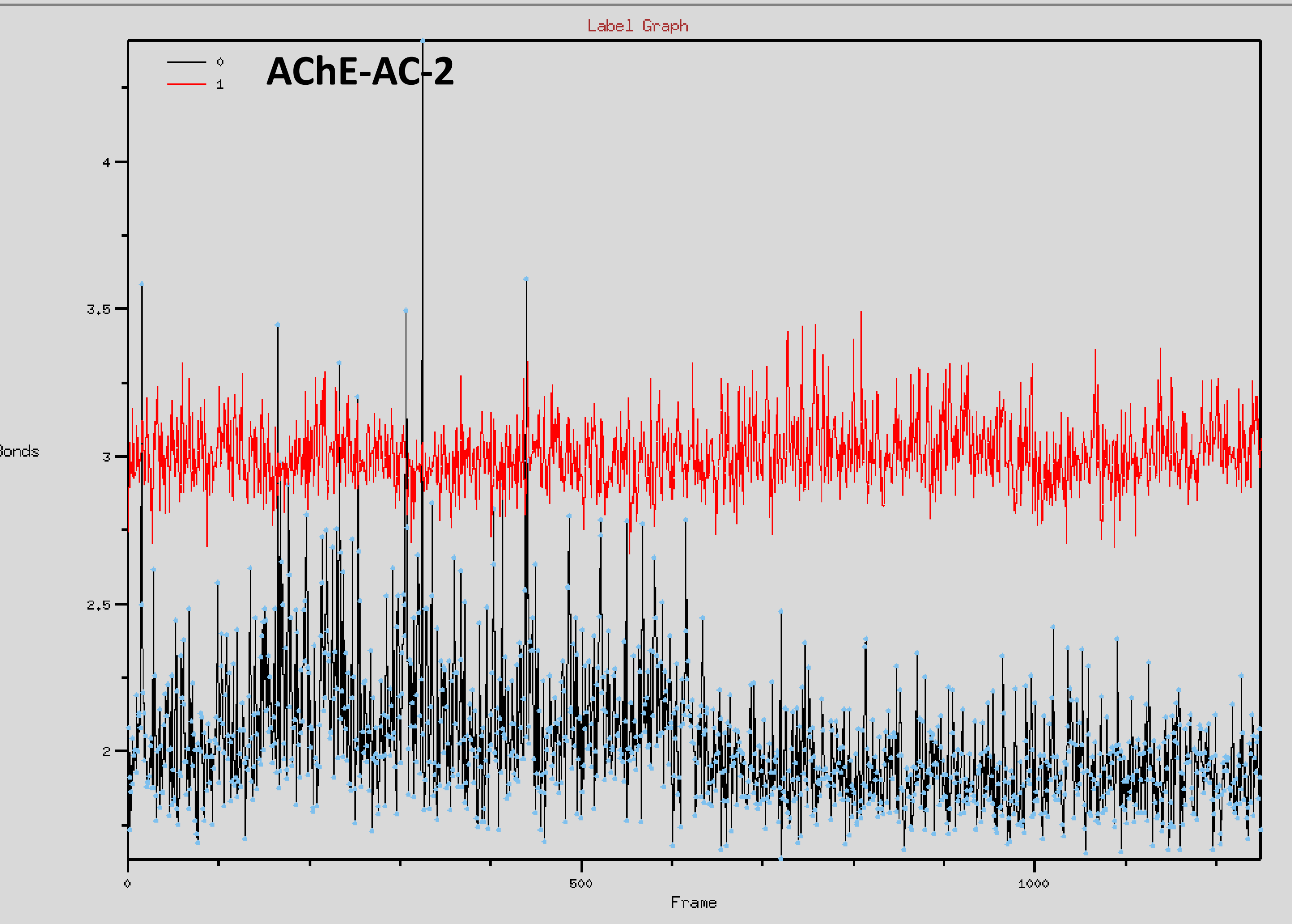
Label Graph
0
1
ACh E-AC-2
4
3.5
3
2.5
2
Bonds
0
500
1000
Frame

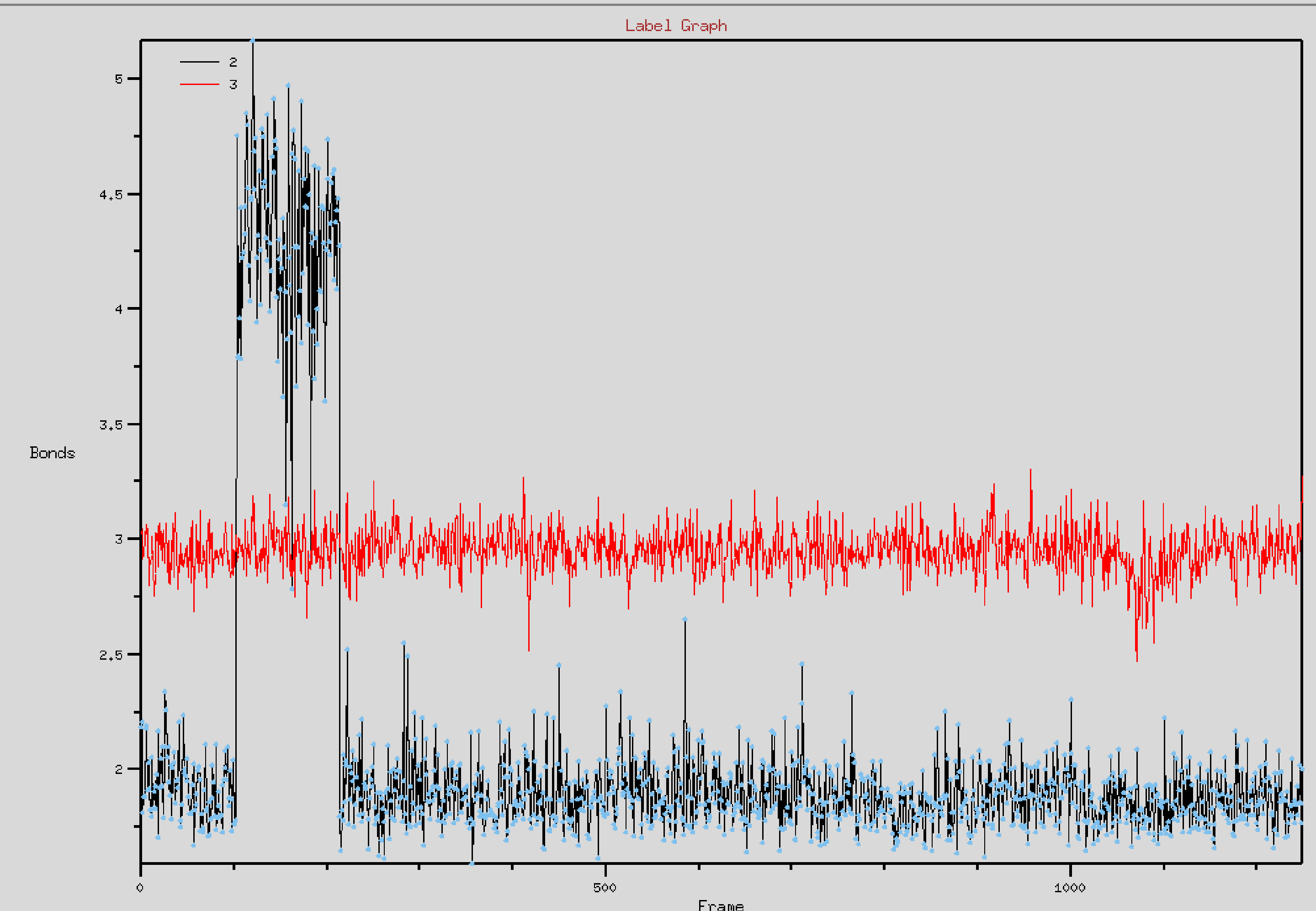
Label Graph
2
3
5
4.5
4
3.5
3
2.5
2
Bonds
0
500
1000
Frame

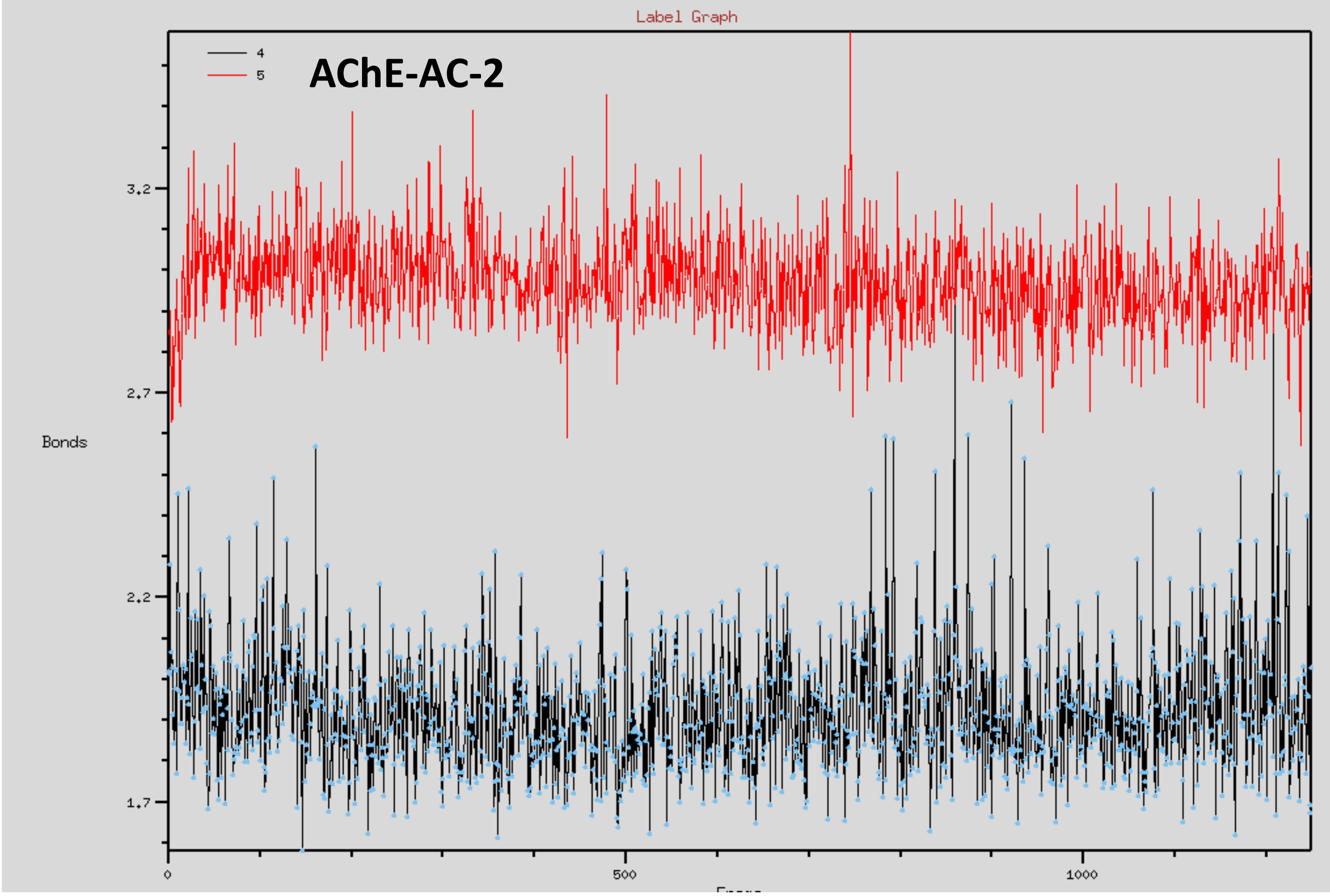
Label Graph
4
5
AChE-AC-2
Bonds
3.2
2.7
2.2
1.7
0
500
1000

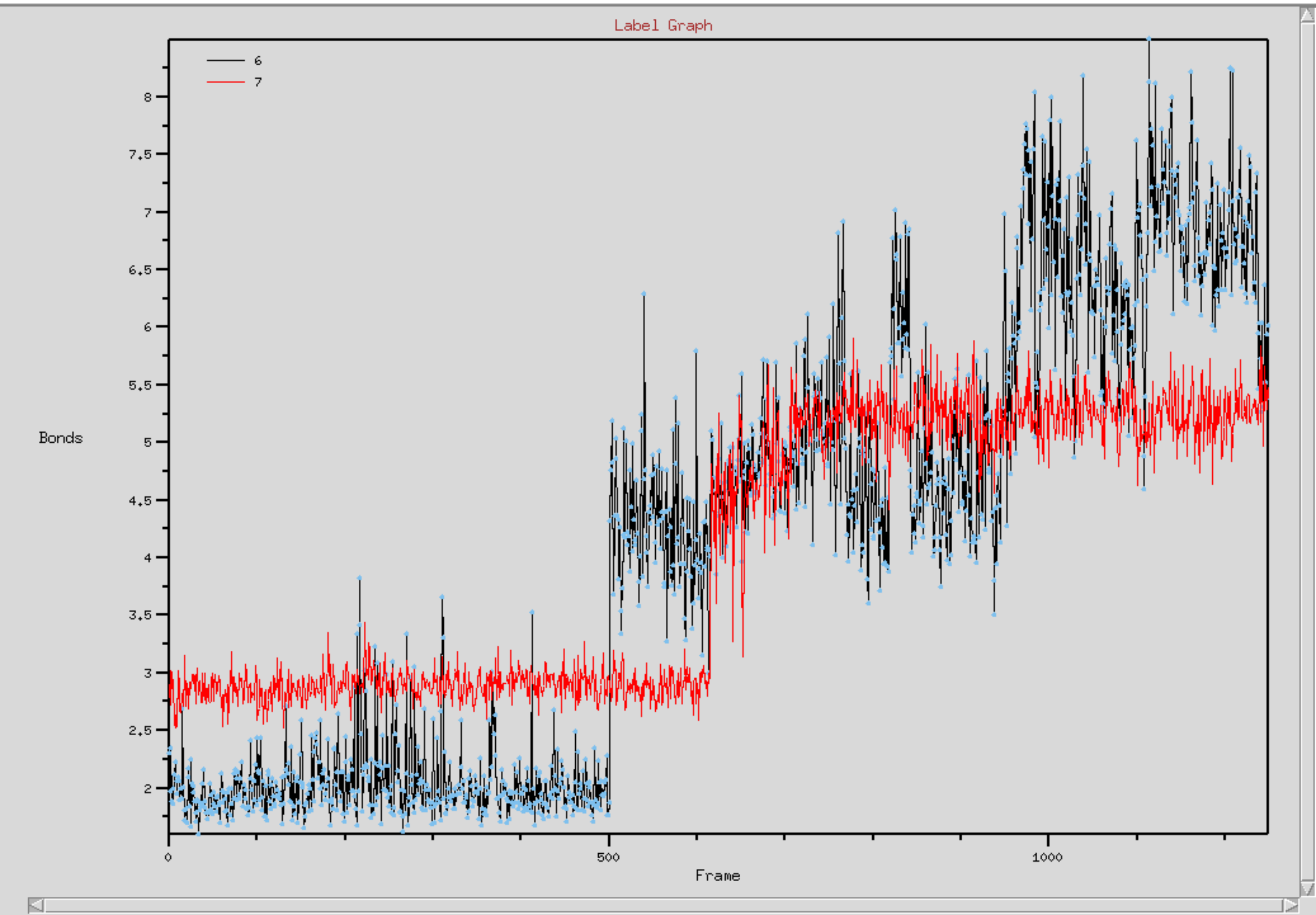
Label Graph
6
7
Bonds
8
7.5
7
6.5
6
5.5
5
4.5
4
3.5
3
2.5
2
0
500
1000
Frame

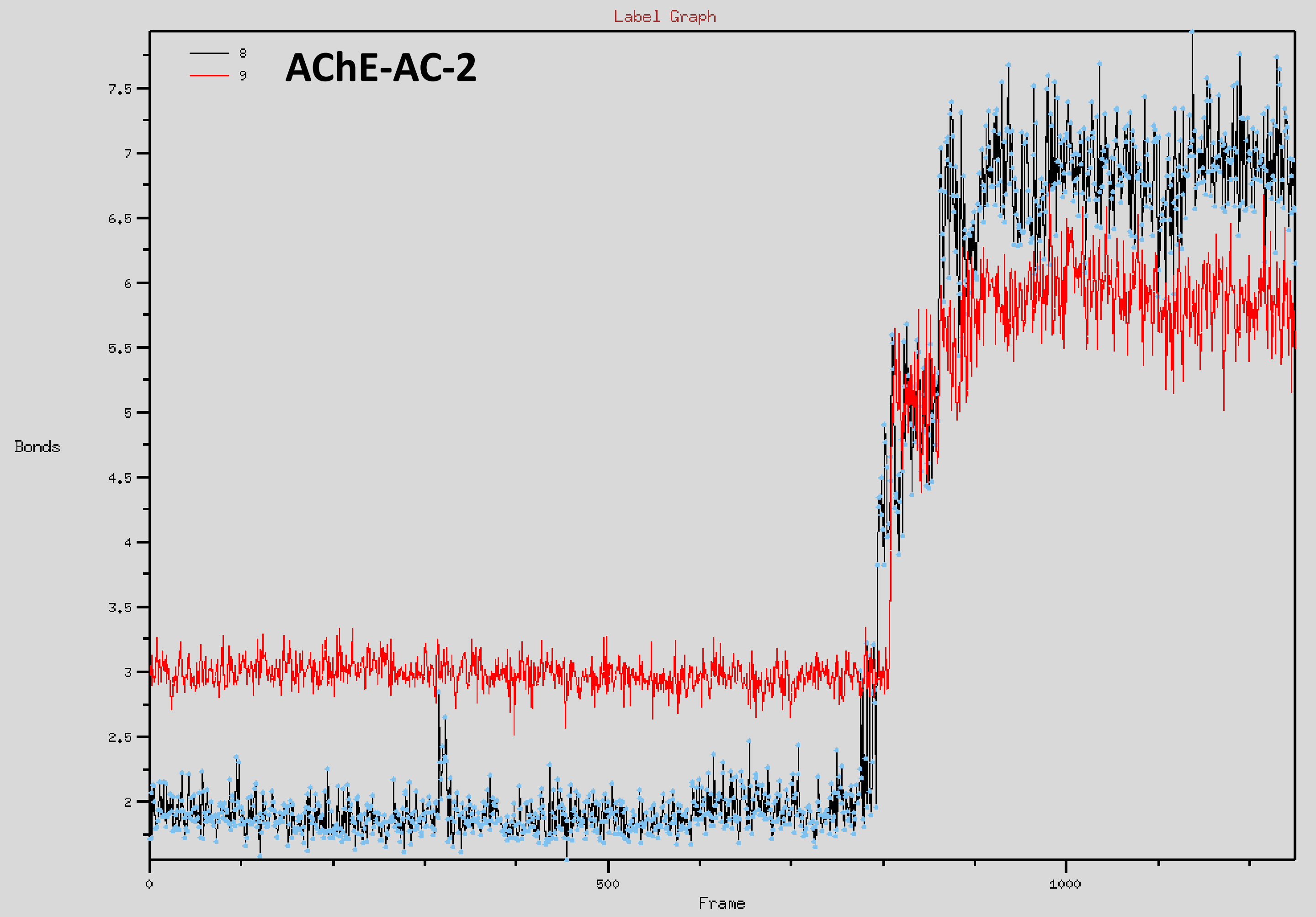

Label Graph
8
9
ACHE-AC-2
Bonds
7.5
7
6.5
6
5.5
5
4.5
4
3.5
3
2.5
2
0
500
1000
Frame

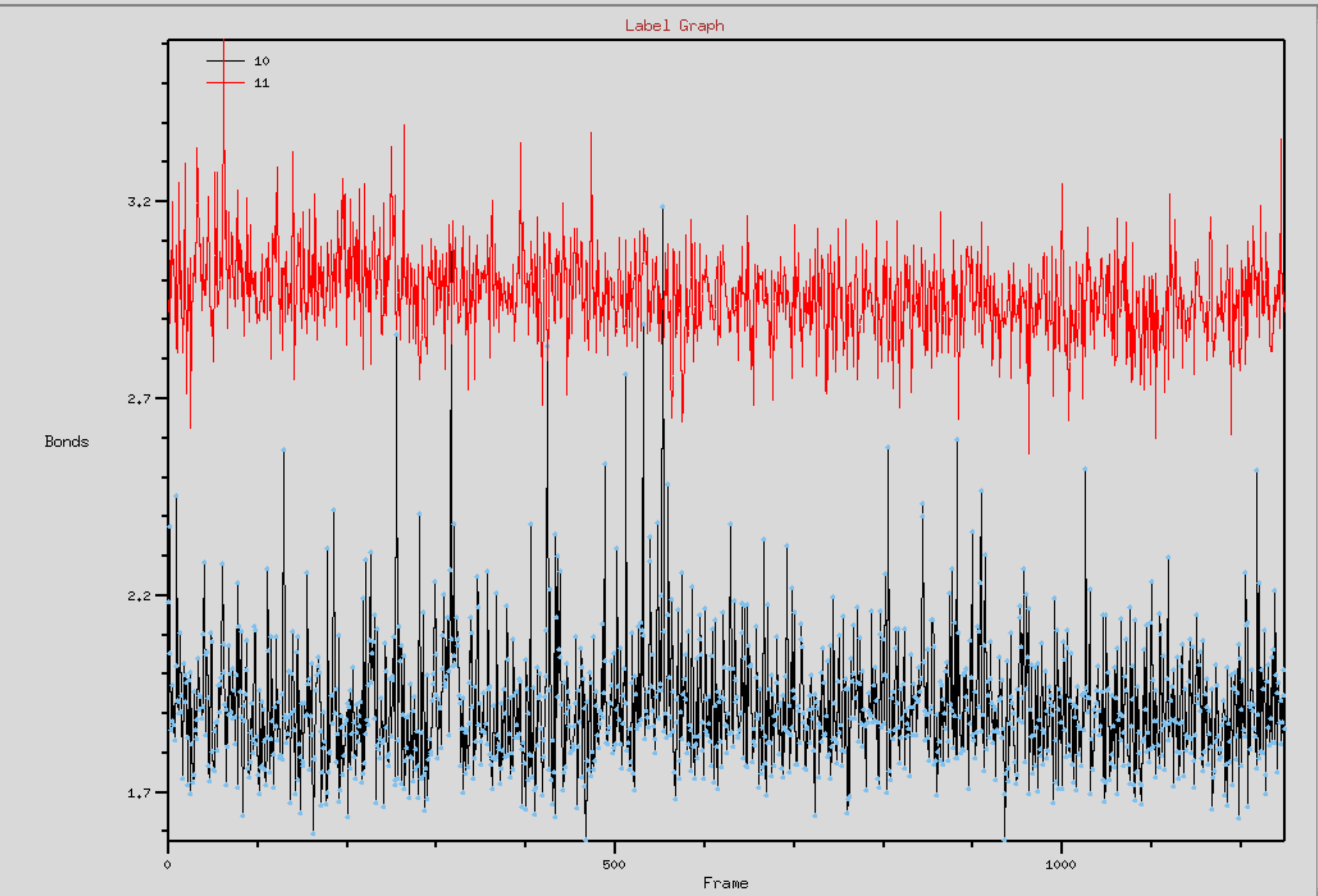

Label Graph
10
11
Bonds
3.2
2.7
2.2
1.7
0
500
1000
Frame

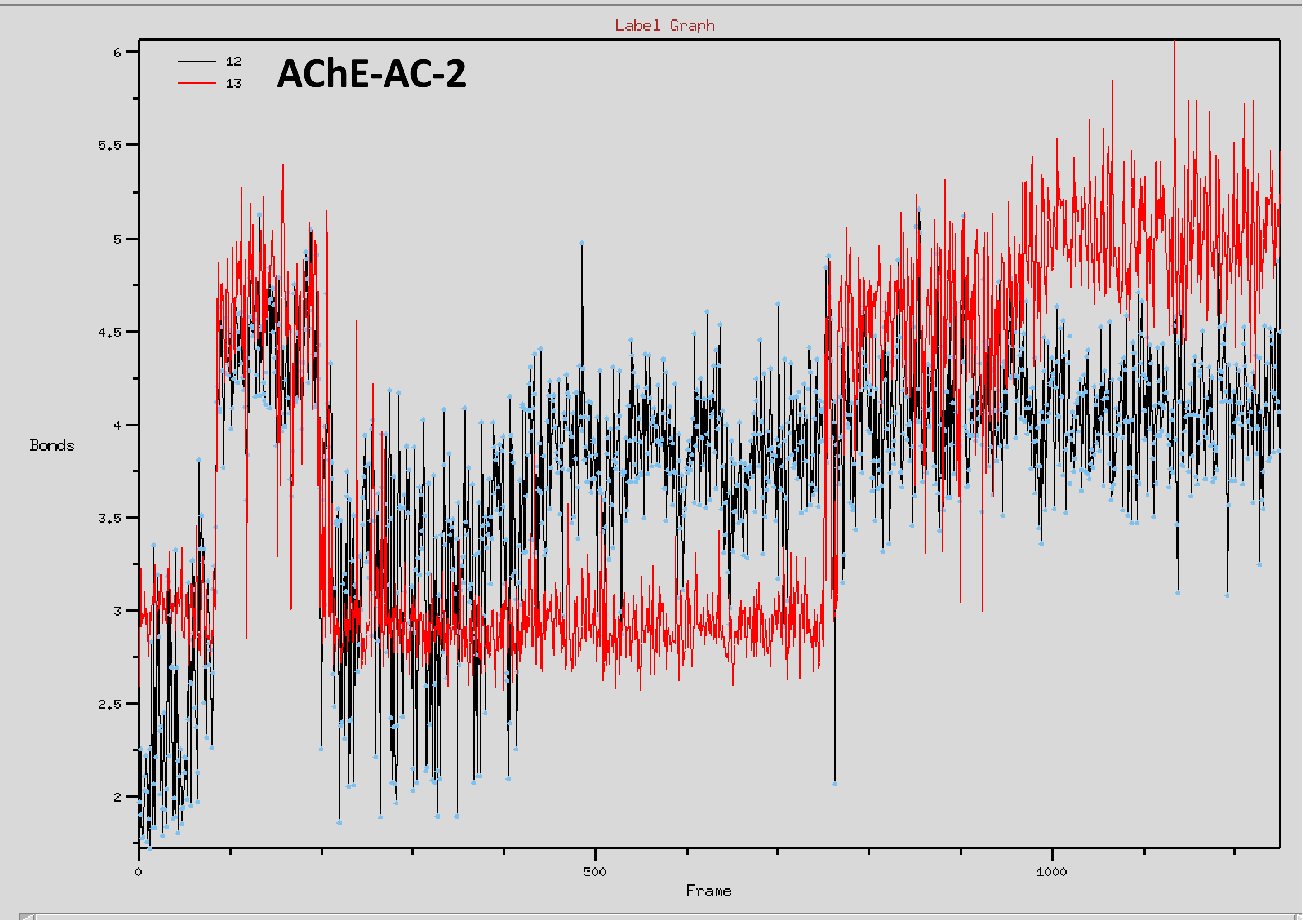
Label Graph
12
13
AChE-AC-2
Bonds
Frame
6
5.5
5
4.5
4
3.5
3
2.5
2
0
500
1000

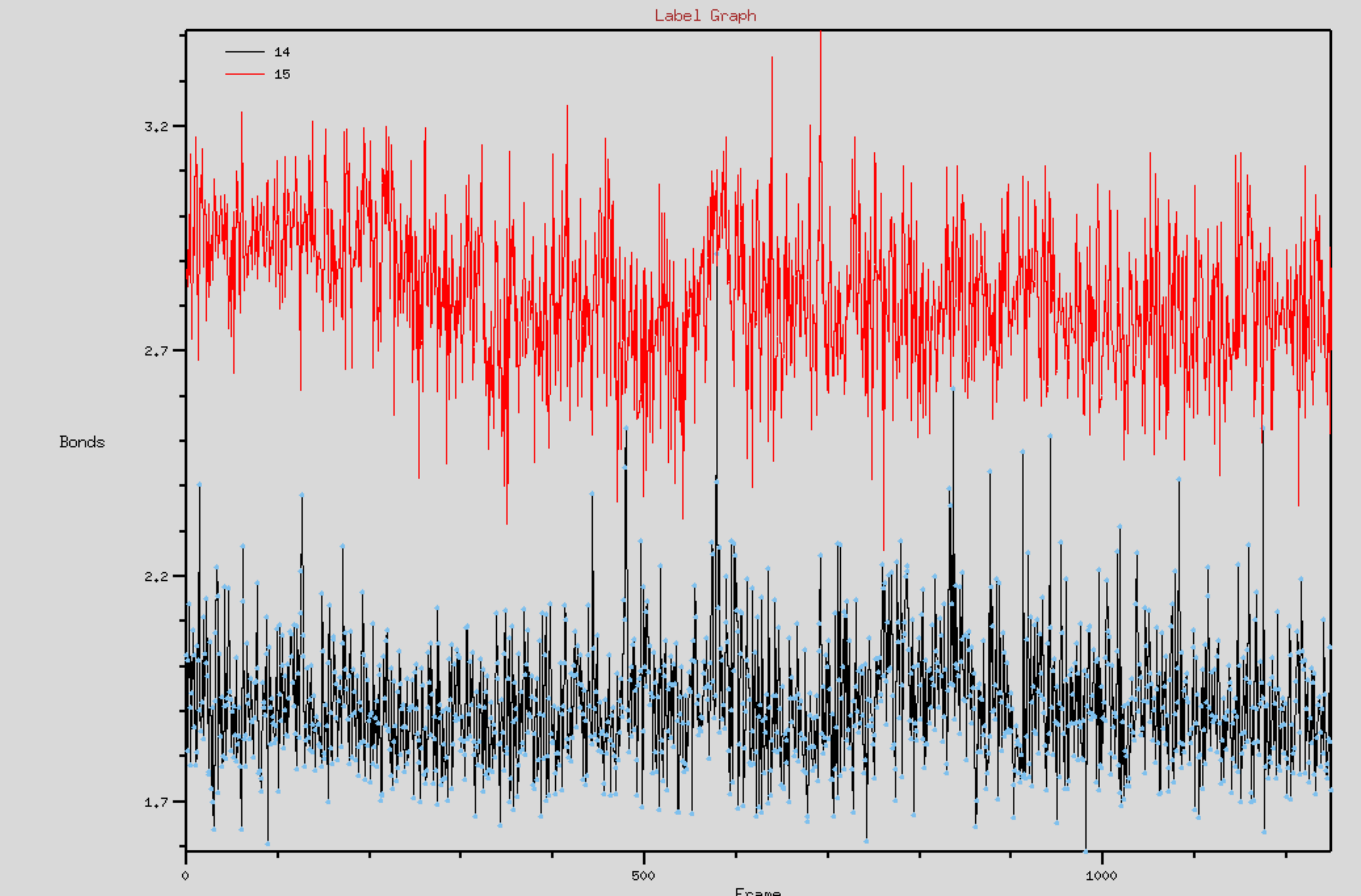
Label Graph
14
15
Bonds
Frame
3.2
2.7
2.2
1.7
0
500
1000

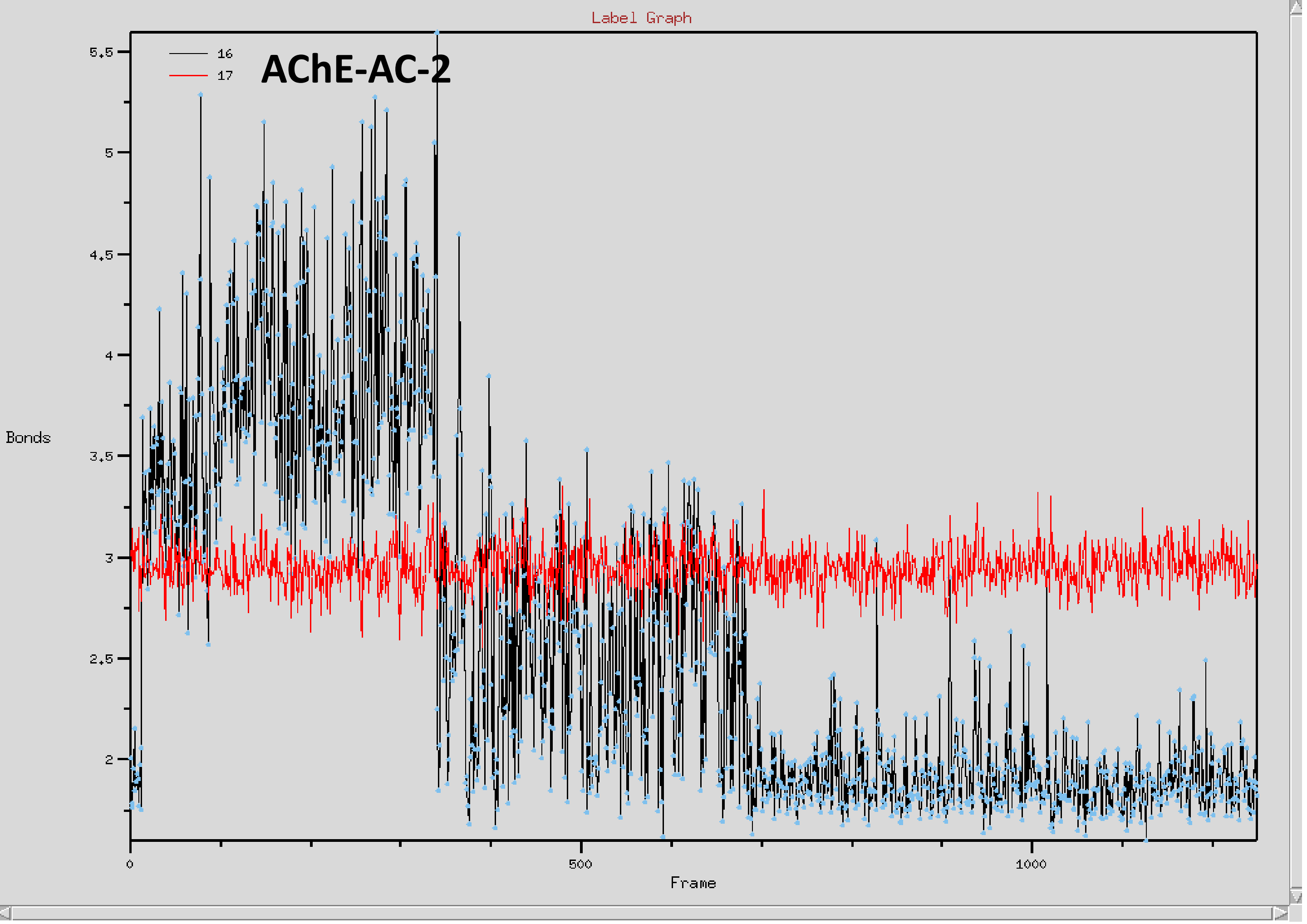
Label Graph
16
17
AChE-AC-2
Bonds
Frame
5.5
5
4.5
4
3.5
3
2.5
2
0
500
1000

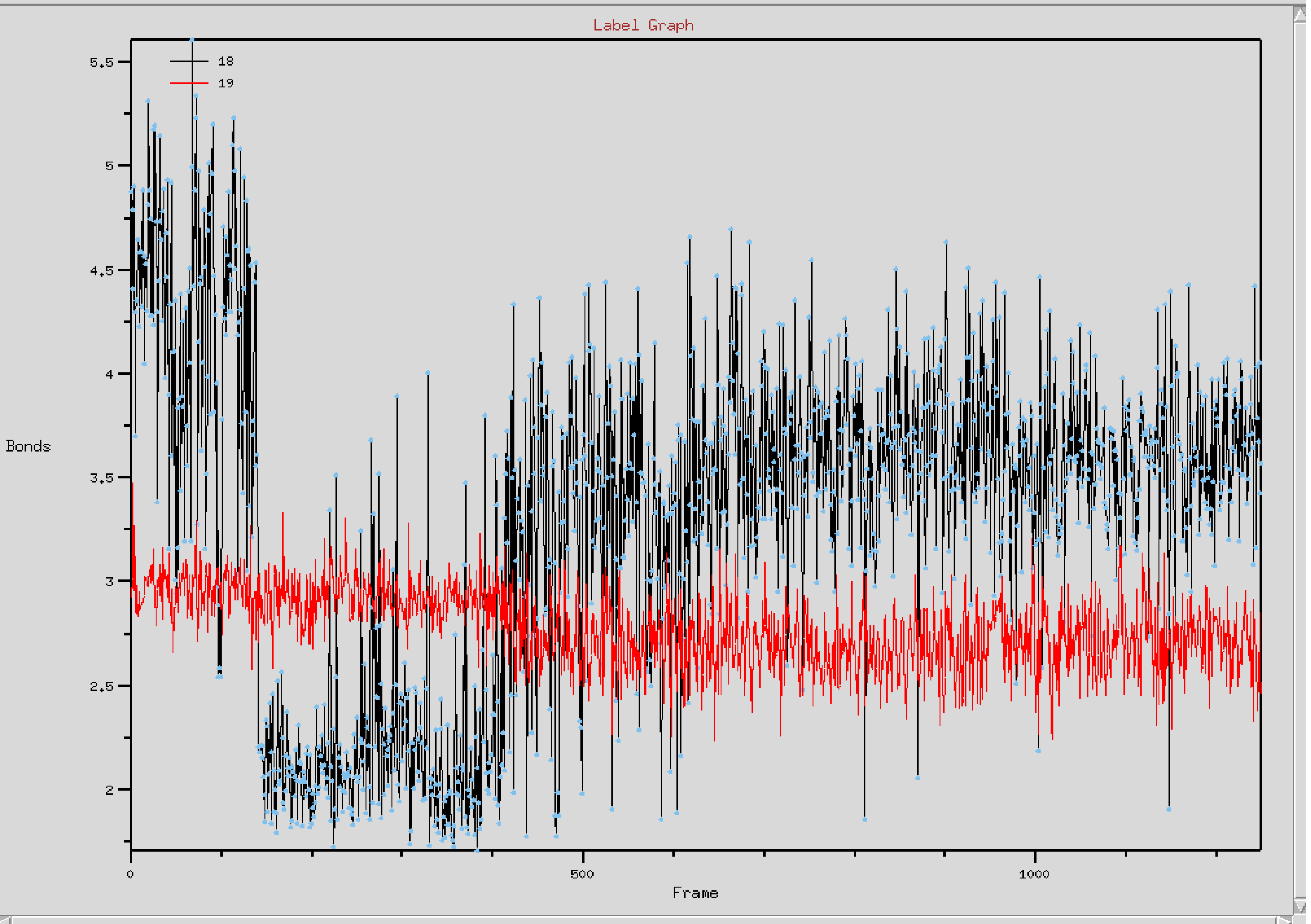
Label Graph
18
19
Bonds
Frame
5.5
5
4.5
4
3.5
3
2.5
2
0
500
1000

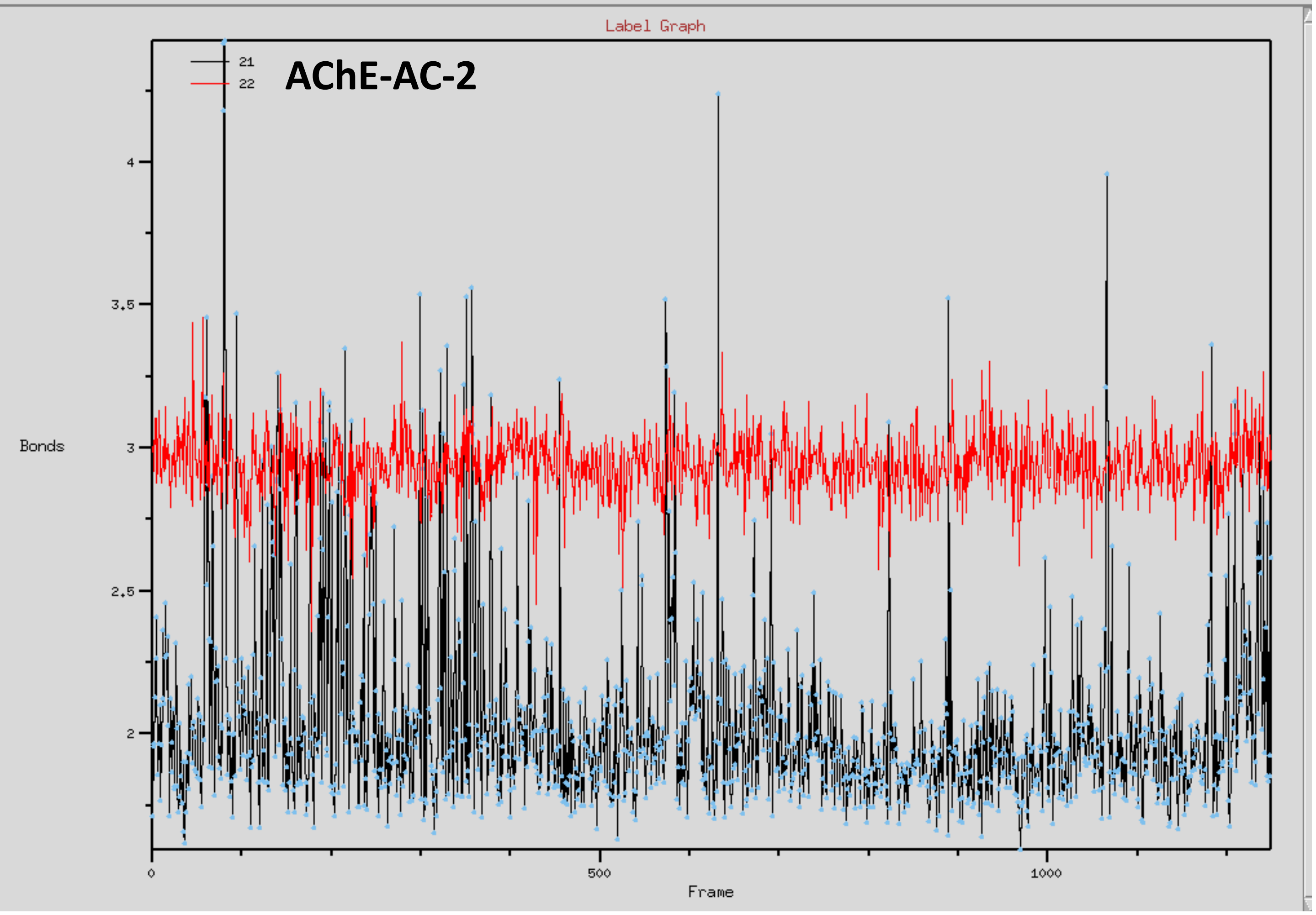
Label Graph
21
22
AChE-AC-2
Bonds
Frame
4
3.5
3
2.5
2
0
500
1000

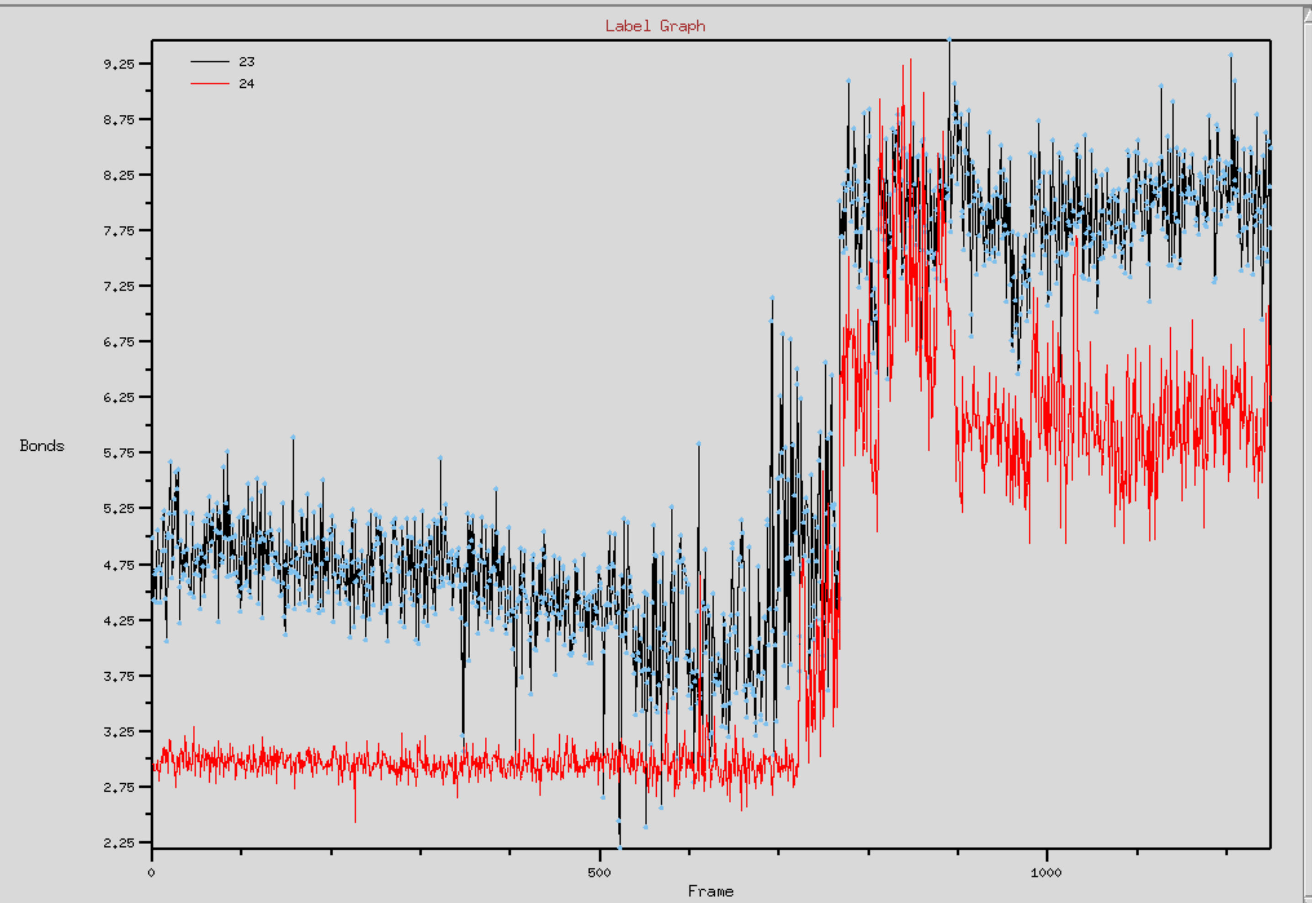
Label Graph
23
24
Bonds
Frame
9.25
8.75
8.25
7.75
7.25
6.75
6.25
5.75
5.25
4.75
4.25
3.75
3.25
2.75
2.25
0
500
1000

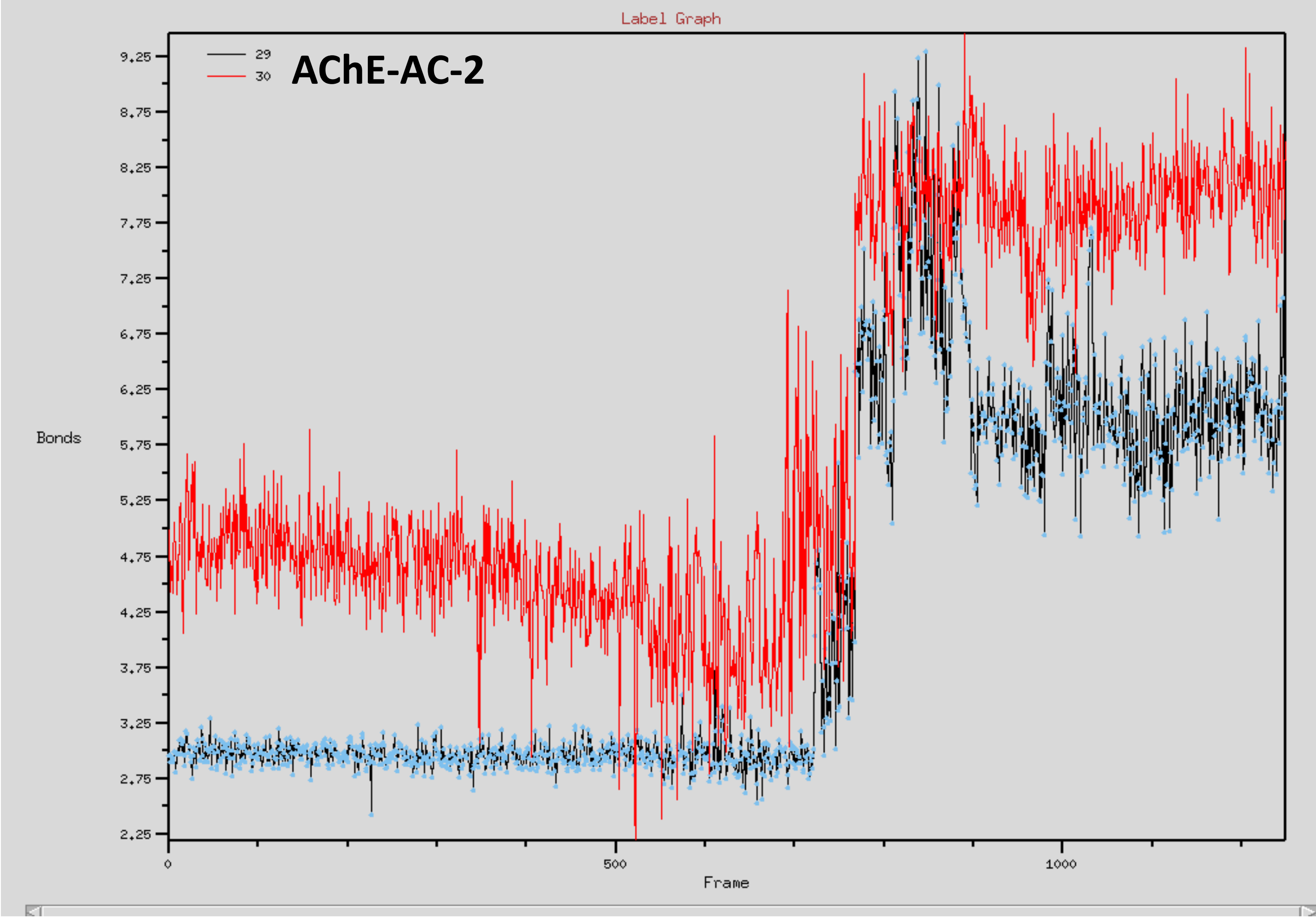
Label Graph
29
30
AChE-AC-2
9.25
8.75
8.25
7.75
7.25
6.75
6.25
5.75
5.25
4.75
4.25
3.75
3.25
2.75
2.25
Bonds
0
500
1000
Frame

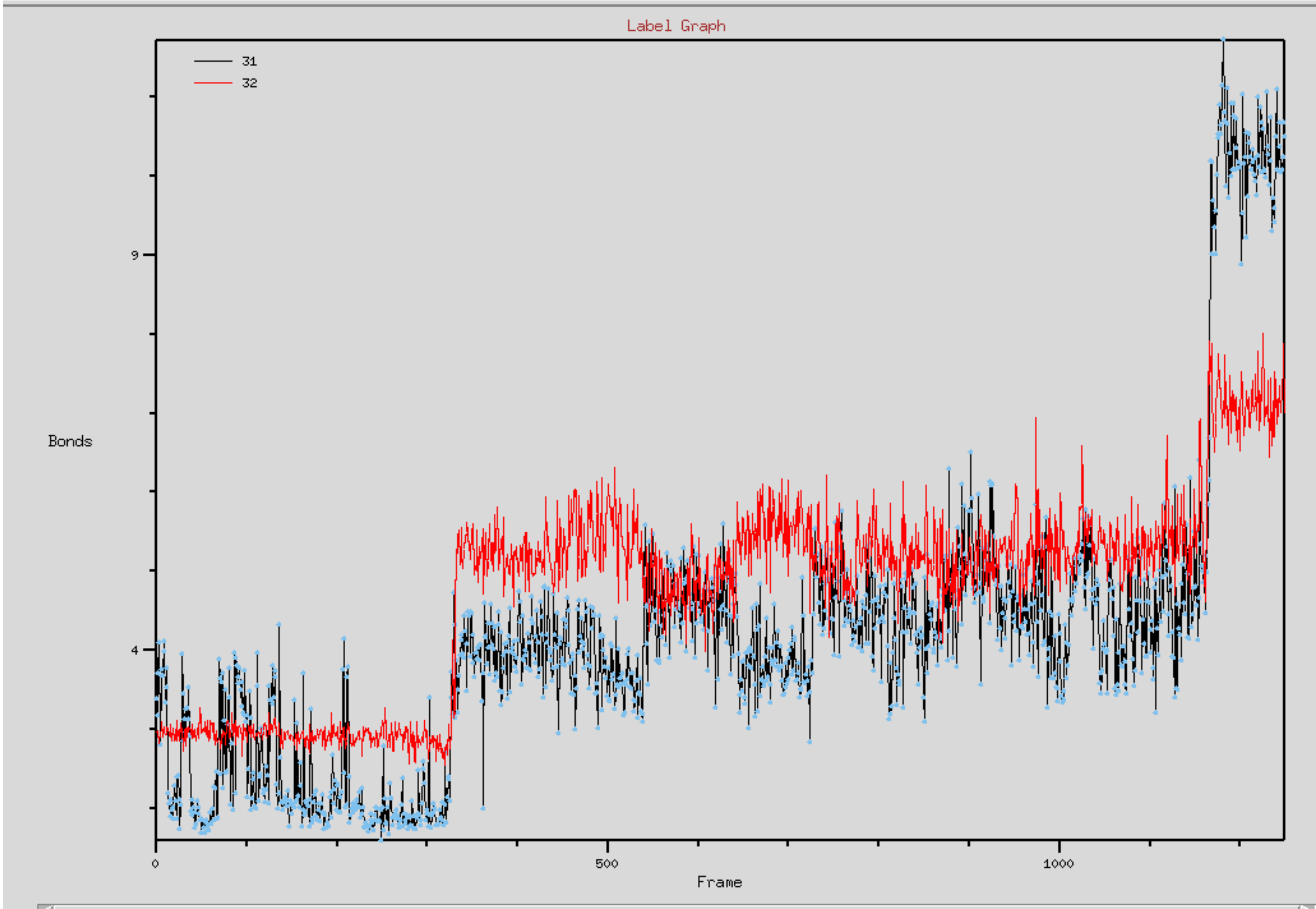
Label Graph
31
32
9
4
Bonds
0
500
1000
Frame

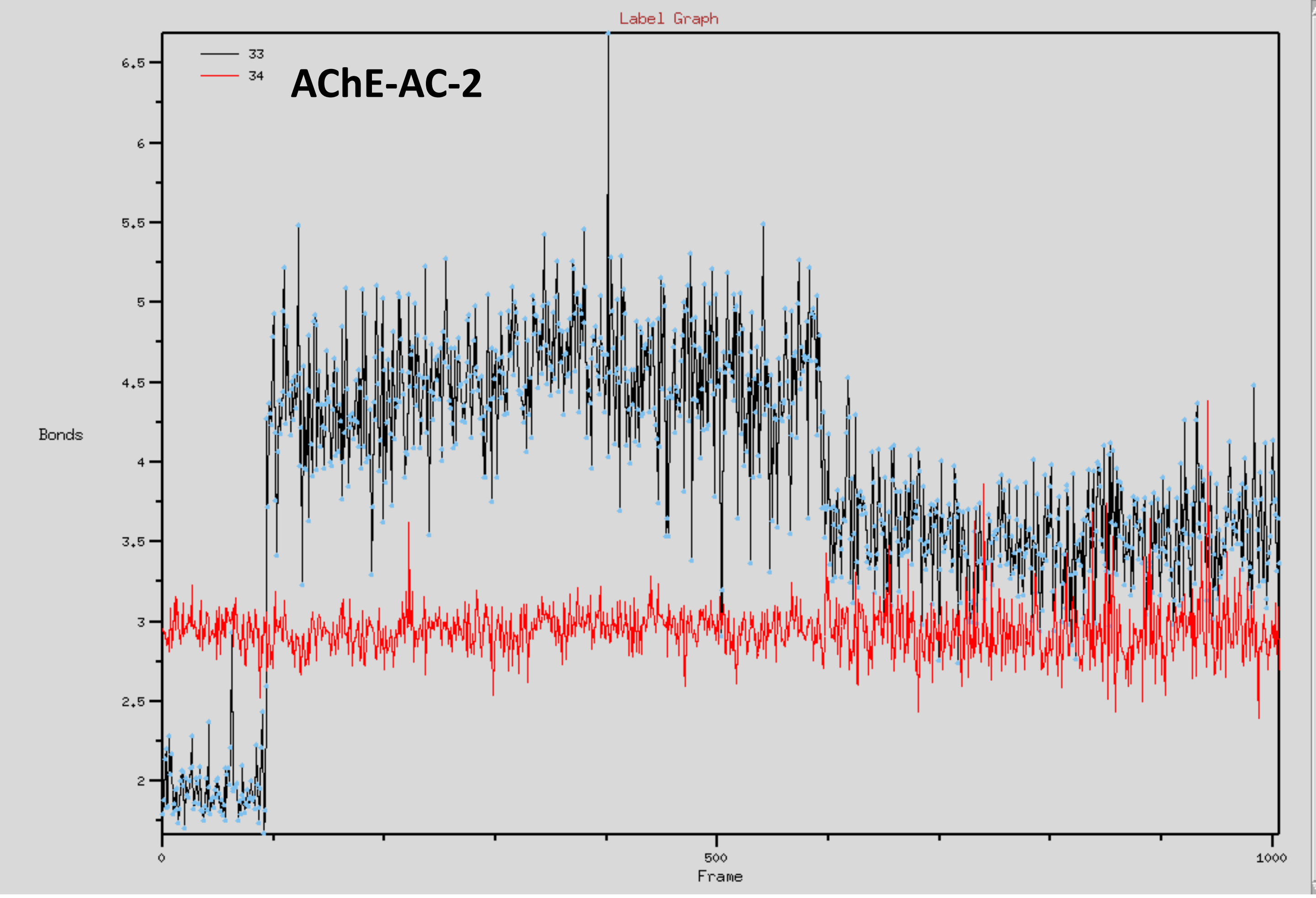
Label Graph
AChE-AC-2
33
34
Bonds
Frame
6.5
6
5.5
5
4.5
4
3.5
3
2.5
2
0
500
1000

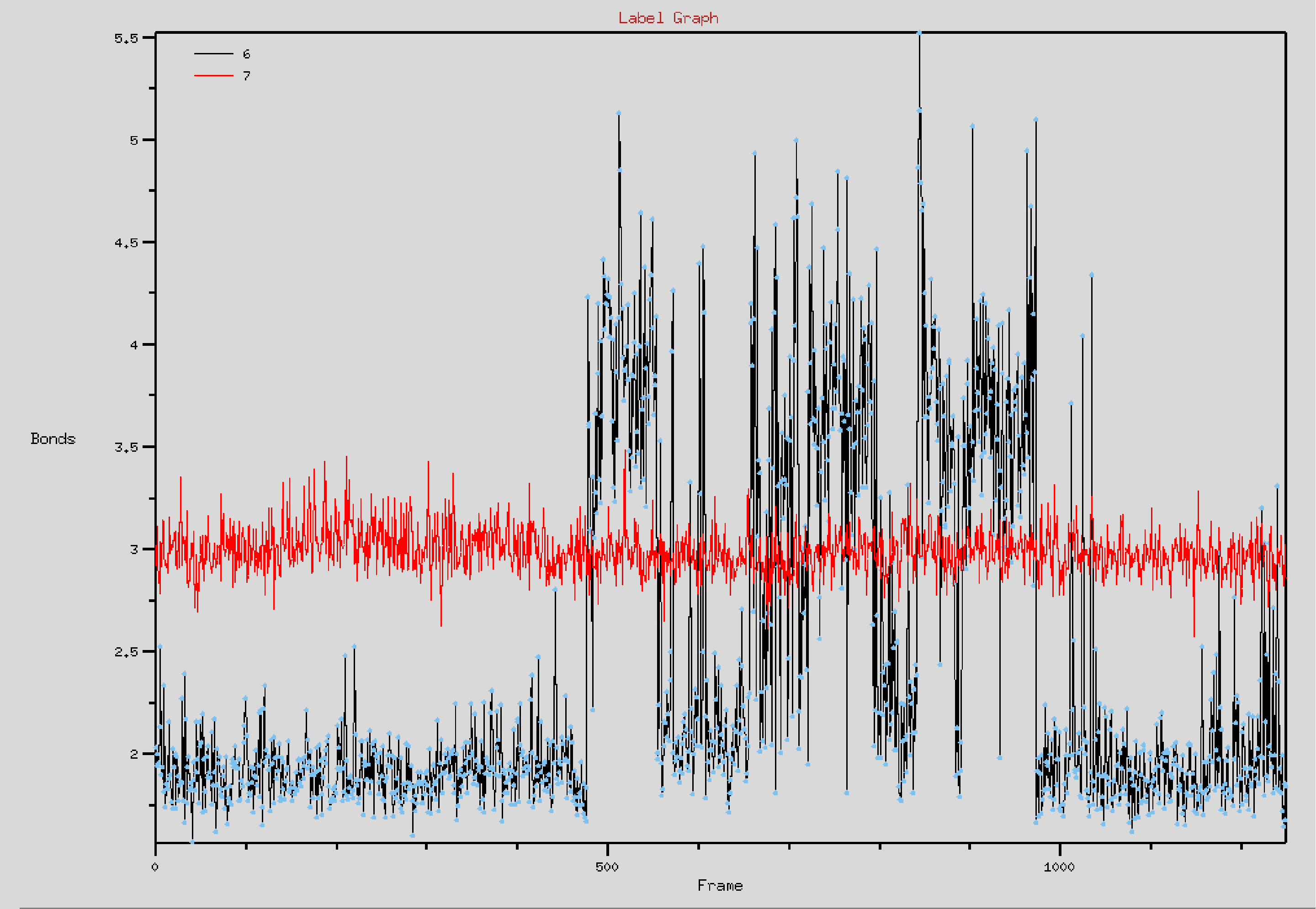
Label Graph
6
7
Bonds
Frame
5.5
5
4.5
4
3.5
3
2.5
2
0
500
1000
